# On the Stability and Degradation Pathways of Venetoclax under Stress Conditions

**DOI:** 10.3390/pharmaceutics12070639

**Published:** 2020-07-07

**Authors:** Nina Žigart, Martin Črnugelj, Janez Ilaš, Zdenko Časar

**Affiliations:** 1 Lek Pharmaceuticals d.d., Sandoz Development Center Slovenia, Analytics Department, SI-1526 Ljubljana, Slovenia; nina.zigart@novartis.com (N.Ž.); martin.crnugelj@sandoz.com (M.Č.); 2 Faculty of Pharmacy, University of Ljubljana, Aškerčeva cesta 7, SI-1000 Ljubljana, Slovenia; janez.ilas@ffa.uni-lj.si

**Keywords:** stability, stress testing, degradation, venetoclax

## Abstract

Venetoclax is an orally bioavailable, B-cell lymphoma-2 (BCL-2) selective inhibitor, used for the treatment of various types of blood cancers, such as chronic lymphocytic leukemia (CLL) and small lymphocytic lymphoma (SLL). In this study we investigated the degradation of venetoclax under various stress conditions including acidic, basic, oxidative, photolytic and thermolytic conditions. We isolated and identified six of its main degradation products produced in forced degradation studies. The structures of the isolated degradation products were determined by using nuclear magnetic resonance (NMR) spectroscopy, high resolution mass spectrometry (HRMS) and infrared (IR) spectroscopy. Additionally, one oxidation degradation product was identified with comparison to a commercially obtained venetoclax impurity. We proposed the key degradation pathways of venetoclax in solution. To the best of our knowledge, no structures of degradation products of venetoclax have been previously published. The study provides novel and primary knowledge of the stability characteristics of venetoclax under stress conditions. Venetoclax is currently the only BCL-2 protein inhibitor on the market. In addition to single agent treatment, it is effective in combinational therapy, so future drug development involving venetoclax can be expected. A better insight into the stability properties of the therapeutic can facilitate future studies involving venetoclax and aid in the search of new similar therapeutics.

## 1. Introduction

**Figure 1:**
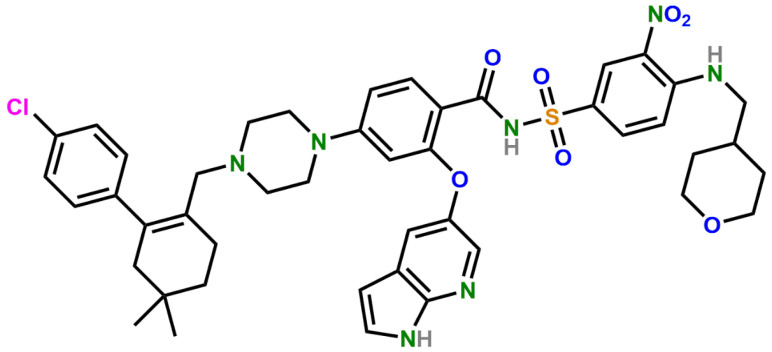
Structure of venetoclax.

Venetoclax (**1**) (Figure 1) is an orally bioavailable, B-cell lymphoma-2 (BCL-2)-selective inhibitor [1,2]. The BCL-2 protein family plays a crucial role in the process of apoptosis [3,4]. Genetically programmed apoptosis can be divided into two pathways: extrinsic and intrinsic. However, the paths are not separate, but ratherinteract. Proteins of the BCL-2 protein family regulate the intrinsic pathway of apoptosis [5,6,7]. The intrinsic pathway is triggered by cell damage. Additionally, most of the anti-cancer agents work by triggering the intrinsic pathway. In the intrinsic pathway, mitochondrial membrane permeabilization and subsequent release of mitochondrial proteins occur, which contribute to apoptotic changes into the cytosol. Both pathways—extrinsic and intrinsic—lead to the activation of caspases that cause degradation or activation of proteins, affecting physiological processes: DNA fragmentation, signaling pathway manipulation and cytoskeletal breakdown [7]. The BCL-2 protein was the first identified protein in the protein family of the same name. The discovery of the anti-apoptotic BCL-2 protein began with an observation of the t(14;18) chromosome translocation in follicular lymphoma and a suggestion of the involvement of gene *bcl-2* in B-cell malignancies with this translocation [8]. From then on, the BCL-2 protein family began to grow and numerous research studies were conducted on the topic [9].

Proteins of the BCL-2 family contain BCL-2 homology (BH) regions (BH1, BH2, BH3 and BH4 domains) [4,10]. They mainly interact via the BH3 domain [11]. Based on the structures and functions of the proteins in the BCL-2 protein family, they can be divided into three groups: pro-apoptotic effector proteins that contain three to four BH domains (BAK, BAX, BOK); pro-apoptotic BH3-only proteins (BAD, BID, BIK, BIM, BMF, HRK, NOXA, PUMA) and anti-apoptotic or pro-survival proteins (BCL-2, BCL-XL, BCL-W, MCL-1, A1/BFL-1) with all four BH domains. BH3-only proteins activate pro-apoptotic effectors that oligomerize upon activation and form pores in mitochondrial membranes, causing mitochondrial outer membrane permeabilization (MOMP) [6,7,12]. Anti-apoptotic BCL-2 proteins bind to the pro-apoptotic BCL-2 proteins and prevent their oligomerization, thus stopping MOMP [5,11,13,14,15]. The elevated concentrations of anti-apoptotic BCL-2 proteins in cancer cells protect the cancer cells from apoptosis, prolonging their lifespan. Therefore, BCL-2 inhibitors, such as venetoclax, induce apoptosis in cells with elevated BCL-2 protein levels. The development of venetoclax was a process that took over 20 years [16].

Venetoclax is used for the treatment of chronic lymphocytic leukemia (CLL), small lymphocytic lymphoma (SLL), acute myeloid leukemia (AML) and mantle cell lymphoma (MCL) as a single agent or in combinational treatment [17,18,19]. There are 175 clinical studies involving venetoclax currently active or recruiting in the U.S. National Library of Medicine Clinical Trials database [20] and numerous research studies investigating the treatment potential of venetoclax [21].

The European Medicines Agency (EMA) assessment report (EPAR) for Venclyxto^TM^ states that venetoclax exhibits sensitivity to light, oxidation and slight sensitivity to UV radiation, acid, heat and a combination of heat and moisture [22]. However, until now there were no reports in the literature which would reveal degradation pathways of venetoclax and the structure of its degradation products.

Therefore, the present study has been designed to conduct forced degradation studies on venetoclax, following International Conference on Harmonization (ICH) guidelines [23,24] and recommendations from previous research [25,26,27,28,29]. Additionally, we isolated key degradation products with preparative liquid chromatography and characterized them with high resolution mass spectrometry (HRMS), NMR and IR analyses. We proposed main degradation pathways of venetoclax under stress conditions.

Stress testing and identification of degradation products is an important part of the drug development process. It is one of the first steps to predict stability challenges and helps with the development of stability-indicating analytical methods [25]. This is crucial with newer drug substances, where there are no known impurities or degradation products available. Therefore, this study provides primary knowledge on the stability of venetoclax and sets the foundation for proper design of the solid dosage forms containing venetoclax from a stability perspective.

## 2. Materials and Methods

### 2.1. Chemicals and Reagents

Venetoclax was obtained from Selvita (Krakow, Poland) and MSN Laboratories (Hyderabad, India). Analytical standard of venetoclax *N*-oxide degradation product was obtained from Dr. Reddy’s Laboratories (Hyderabad, India). Gradient grade acetonitrile (ACN) and methanol (MeOH) were purchased from J. T. Baker, now part of Avantor^®^ (Radnor, PA, USA). Analytical grade glacial acetic acid, analytical grade EMSURE^®^ ethyl acetate (AcOEt), tetrahydrofurane (THF), trifluoroacetic acid (TFA) for spectroscopy, hydrochloric acid (HCl) Titrisol^®^ solution, sodium hydroxide (NaOH) Titrisol^®^ solution, analytical grade 30% aqueous peroxide solution, iron (III) chloride hexahydrate, FT-IR grade potassium bromide (KBr), LC-MS grade LiChropur^®^ ammonium bicarbonate, GC grade dichloromethane (DCM) and analytical grade ammonium bicarbonate were purchased from Merck KGaA (Darmstadt, Germany). Dimethyl sulfoxide (DMSO) was purchased from Honeywell (Charlotte, NC, USA). MS grade HiPerSolv CHROMANORM^®^ ACN was purchased from VWR, part of Avantor (Radnor, PA, USA). Purified water was obtained by filtrating through a Milli-Q^®^ system from Merck Millipore (Burlington, MA, USA).

### 2.2. Equipment and Software

Liquid chromatography (LC) analyses were performed on: Acquity UPLC^TM^ systems (Waters, Millford, MA, USA) equipped with binary solvent manager (BSM), sample manager (SM), temperature-controlled column compartment and photodiode array (PDA) detector; Acquity UPLC^TM^ system (Waters, Millford, MA, USA) equipped with BSM, SM, PDA detector and a high sensitivity flowcell, column manager (CM) and an additional solvent switch; and Acquity UPLC^TM^ H-Class systems (Waters, Millford, MA, USA) equipped with quaternary solvent manager (QSM), sample manager with flow-through needle (SM-FTN) and either PDA or tunable ultraviolet (TUV) optical detector. Liquid chromatography coupled with mass spectrometry (LC-MS) was performed on an Acquity UPLC^TM^ system with a Micromass^®^ Quatro Premier XE^TM^ mass spectrometer (Waters, Millford, MA, USA) equipped with BSM, SM, CM and PDA detector. High-resolution mass spectrometry was performed on Orbitrap Q Exactive^®^ mass spectrometer (Thermo Fisher Scientific, Waltham, MA, USA). Biotage^®^ Isolera^TM^ flash chromatography system (Biotage, Uppsala, Sweden) and Agilent^®^ 1200 Series system (Agilent Technologies, Santa Clara, CA, USA) with two G1361A preparative pumps, G1315D diode array detector and Rheodyne^®^ model 3725i preparative sample injector (Sigma-Aldrich by Merck, St. Louis, MO, USA) were used for separation and isolation of degradation products. All NMR measurements were carried out on a Bruker Avance^®^ III 500 MHz spectrometer (Bruker Biospin, Rheinstetten, Germany). The spectrometer was equipped with a 5 mm Broad Band Observe (BBO) Z-gradient probe. Spectra were acquired and processed using Bruker TopSpin^®^ software, version 3.1. IR spectra were acquired using a Nicolet^®^ iS^TM^50 FT-IR spectrometer (Thermo Fisher Scientific, Waltham, MA, USA). Thermal analysis was done on a DSC 3+ system (Mettler Toledo, Columbus, OH, USA).

The weighing was done on either an XP4002S precision balance, an XP205 DeltaRange^®^ analytical balance, an AX205 DeltaRange^®^ analytical balance or an MX5 microbalance (Mettler Toledo, Columbus, OH, USA). Weighing of venetoclax drug substance was done in a ventilated balance enclosure OK 15 (Iskra Pio, Šentjernej, Slovenia). pH was measured using a SevenMulti^TM^ pH meter (Mettler Toledo, Columbus, OH, USA). Evaporation of solvents in isolated products was done with an IKA RV 10 rotary evaporator (IKA-Werke, Staufen im Breisgau, Germany) equipped with an IKA HB 10 heating bath and an MD4C vacuum-pump system (Vacuubrand, Wertheim, Germany), and a TurboVap^®^ LV evaporator (Caliper Life Sciences, Waltham, MA, USA). Pipettes used were Picus automatic pipettes (Sartorius, Göttingen, Germany) and Handystep^®^ electronic repetitive pipettes (Brand, Wertheim, Germany). Ultrasonic baths used were Branson 8510 (Emerson Electric, St. Louis, MO, USA), Sonic 10 and Sonic 20 (Iskra Pio, Šentjernej, Slovenia). Degradation studies were done in a BF 720 standard incubator (Binder, Tuttlingen, Germany). Photo-stability was performed in a Suntest^®^ XLS+ xenon test instrument (Atlas Material Testing Technology part of Ametek, Mount Prospect, IL, USA).

Waters LC systems were equipped with Empower^®^ 3 chromatography data software (Waters, Millford, MA, USA), MS was run by MassLynx^®^ mass spectrometry software (Waters, Millford, MA, USA), HRMS was equipped with Xcalibur^TM^ software (Thermo Fisher Scientific, Waltham, MA, USA) and preparative HPLC system was equipped with Agilent ChemStation^®^ software (Agilent Technologies, Santa Clara, CA, USA).

### 2.3. Ultra High Performance Liquid Chromatography (UHPLC) Method Conditions

The UHPLC method was used for degradation testing. Method conditions were: sample solvent composition: ACN-DMSO-buffer (6:3:1, *v/v/v*); column: Acquity UPLC^TM^ CSH C18 (1.7 μm, 100 mm × 2.1 mm); mobile phase A:A = ammonium bicarbonate (pH 6.0; 10 mM) pH adjusted with acetic acid-ACN (9:1, *v/v*); mobile phase B:B = 100% ACN; strong needle wash: water-ACN-DMSO (5:4:1, *v/v/v*); pump flow 0.6 mL/min; injection volume 5 µL; column temperature 60 °C; autosampler temperature 5 °C; detection wavelength 220 nm; gradient: *t* = 0 min, 0% B; *t* = 3 min, 0% B; *t* = 10 min, 70% B; *t* = 12 min, 70% B; *t* = 16 min, 80% B; *t* = 18 min, 0% B; re-equilibration = 2 min.

### 2.4. MS and HRMS Method Conditions

MS conditions were: ion mode ESI+, capillary voltage 2.00 kV, cone voltage 40 V, extractor voltage 3 V, RF lens voltage 0.0 V, source temperature 110 °C, desolvation temperature 350 °C, desolvation gas flow 600 L/h, cone gas flow 60 L/h. HRMS spectra were acquired using direct injection (flow 20 µL/min). HRMS conditions were: spray voltage +3600 V, capillary temperature 350 °C, sheath gas flow rate 8 arb. units and auxiliary gas flow rate 4 arb. units.

### 2.5. Preparative Chromatography Conditions

Flash chromatography was used mainly to separate venetoclax from its degradation products. Separation was performed on Biotage^®^ SNAP 50 columns with a gradient elution using dichloromethane (DCM) as a weak solvent and MeOH as a strong solvent. Collected fractions corresponding to visible peaks under UV spectrum at 220 nm were then combined and solvents evaporated.

Preparative HPLC method was developed using a Phenomenex Luna^®^ C18 column (5 μm, 250 mm × 21.2 mm). Mobile phases were: A = 0.1% TFA in water (*v/v* %); B = ACN-A (9:1, *v/v*). The gradient was changed depending on the sample. First gradient was: 0 min, 10% B; 2 min, 10% B; 12 min, 90% B; 14 min, 100% B; 16 min, 100% B; 16.5 min, 10% B; and was used for the preparation of degradation products B1, B2 and A2. Second gradient was: 0 min, 10% B; 2 min, 10% B; 3 min, 60% B; 17 min, 90% B; 17.5 min, 100% B; 19 min, 100% B; 20 min, 10% B; and was used for the preparation of degradation products A1, A3 and A4. Flow rate was 15 mL/min and detection wavelength was 220 nm. The column temperature was not controlled. The sample was injected by hand in multiple smaller portions of approximately 0.2–0.4 mL accumulating to 2–10 mL of sample per run depending on the sample. Sample fractions were collected by hand.

### 2.6. NMR Method Conditions

NMR spectrometer was operated at 500 and 150 MHz for ^1^H and ^13^C, respectively. Samples were prepared by dissolving in DMSO-*d_6_* (D, 99.8%, Merck) and spectra were recorded at room temperature. Chemical shifts (*δ*) were expressed in ppm with reference to residual solvent signal (2.50 ppm and 39.5 ppm for ^1^H and ^13^C, respectively).

### 2.7. DSC Measurements Conditions

DSC thermograms were acquired using the differential scanning calorimeter DSC 3+ system (Mettler Toledo, Columbus, OH, USA) operating at 10 °C/min.

### 2.8. Fourier-Transform Infrared (FTIR) Measurements Conditions

FTIR spectra were acquired using Nicolet^®^ iS^TM^50 FT-IR spectrometer (Thermo Fisher Scientific, Waltham, MA, USA) using KBr disks.

### 2.9. Preparation of Sample Solutions

#### 2.9.1. Degradation Studies Samples

All of the samples were prepared in a dark room. Because of venetoclax’s low solubility in water, an initial solution of venetoclax in DMSO was prepared. DMSO was used as a co-solvent in stress testing to obtain a solution [26]. Around 250 mg of venetoclax substance was weighed in 100 mL of DMSO, resulting in a solution with a concentration of about 2.5 mg/mL. The initial solution was divided in 10 mL portions and 10 mL of the stress medium were added—aqueous solutions (1 M HCl, 1 M NaOH, 3% H_2_O_2_, 1 mg/mL ACVA). The flasks were sealed and put in a standard incubator chamber with a regulated temperature of 50 °C ± 2 °C. Where the stressor was merely room temperature or elevated temperature, 20 mL of ACN and 3.33 mL of buffer, ammonium bicarbonate (pH 6.0, 10 mM), were added to the 10 mL of initial DMSO solution of venetoclax to get an approximate UHPLC sample solvent composition (ACN-DMSO-buffer (6:3:1, *v/v/v*)).

The prepared samples were sampled at different time points (1, 2, 3, 4, 7, 8, 9, 10, 11 and 14 days) by transferring 2 mL of the sample into a 5 mL flask. HCl and NaOH stress sample solutions were neutralized by adding 1 mL of either 1 M HCl or 1 M NaOH, then 0.5 mL of DMSO was added and the flask was topped off with ACN. Where the stress solution added was H_2_O_2_ or ACVA, there was no acid/base neutralization. DMSO (0.5 mL) was added to the initial 2 mL of stress sample solution and the flask was filled with ACN. The samples that were on room temperature and 50 °C, without an additional stress medium, were diluted with the UHPLC solvent.

#### 2.9.2. Samples for Isolation of Degradation Products

Venetoclax drug substance (1 g) was weighed and dissolved in 100 mL of DMSO, then 50 mL of 4 M HCl was added to the solution. In the same manner, 1 g of venetoclax drug substance was dissolved in 100 mL of DMSO, then 50 mL of 4 M NaOH was added to the solution. The prepared samples were incubated in a standard incubator chamber set at 50 °C ± 2 °C for 7 days. The samples were neutralized with 50 mL of either 4 M NaOH or 4 M HCl. Then, 50 mL of ACN was added. A small amount of sample was taken for analysis. The sample solvent was then evaporated with a rotary evaporator and residual DMSO was removed with distillation. The sample was analyzed with UHPLC to assess the effect of distillation. In another instance, DMSO was removed by multiple liquid–liquid extractions with AcOEt and DCM. The extracted samples were analyzed with LC-MS. Dry samples were later used for Flash chromatography. Collected samples from Flash chromatography were used for preparative HPLC. Samples were reconstituted in the solvent for preparative HPLC (THF-0.1% (*v/v* %) TFA in water (1:1, *v/v*)). Samples were always reconstituted directly before isolation to reduce the oxidation which could lead from exposure to THF.

#### 2.9.3. Preparation of Isolated Degradation Products for Analyses

After samples from preparative chromatography were collected, the solvent was removed by evaporation to obtain dry degradation products. For NMR analyses, degradation products were dissolved in DMSO-*d_6_*. The solutions were then used for HRMS. DMSO was partially evaporated with a rotary evaporator and samples were diluted with MeOH to achieve the concentration of 1 µg/mL. For thermal analysis, dry samples were weighed in aluminum sample pans and encapsulated. To measure the IR spectra, the samples were mixed with KBr and pressed into tablets for analysis.

## 3. Results and Discussion

### 3.1. Forced Degradation Studies

**Figure 2:**
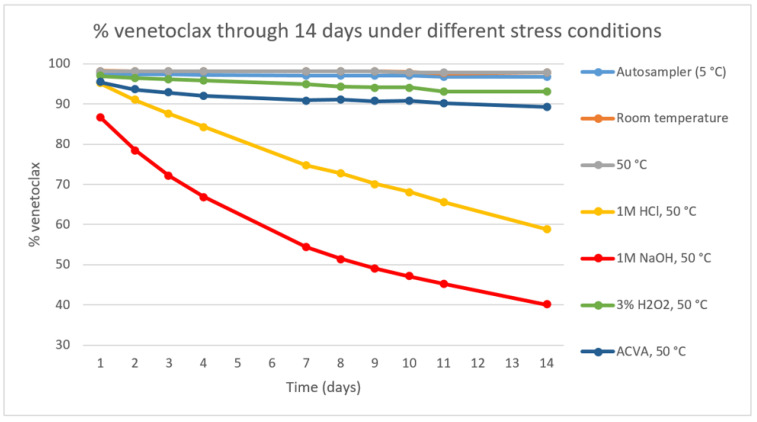
Line chart representing the percentage of venetoclax in the stress samples under various stress conditions over 14 days. The content was determined with an UHPLC method.

We conducted forced degradation studies based on the ICH guidelines [23,24]. The primary stress conditions we chose were 0.1 M HCl, 1 M HCl, 0.1 M NaOH, 1 M NaOH, 0.3% H_2_O_2_, 3% H_2_O_2_, FeCl_3_ and 22 h SUNTEST. The SUNTEST irradiance was 250 W/m^2^ at wavelengths of 300-800 nm, corresponding to approximately 1.28 mio lux hours. The temperature was kept under 30 °C in the test chamber throughout the test, with the black standard surface maximum temperature set to 40 °C. All the stress testing, except for the SUNTEST, was conducted in an incubator chamber at 50 °C ± 2 °C. After an initial assessment of the stability at the mentioned conditions, a degradation study was conducted lasting 14 days where the substance in DMSO solution was exposed to room temperature, elevated temperature of 50 °C, 1 M HCl at 50 °C, 1 M NaOH at 50 °C, 3% H_2_O_2_ at 50 °C and ACVA at 50 °C. Venetoclax has shown to be sensitive to acidic and basic conditions in solution and partially sensitive to oxidation. The elevated temperature of 50 °C as a sole stressor did not result in a significant degradation (Figure 2).

A degradation of 5–20% [29] or 10–20% [26] can be found as a recommendation for stress testing, to produce relevant degradation products. A degradation >5% was achieved in acidic, basic and oxidative conditions, and a degradation of at least 10% was achieved with added HCl, NaOH and ACVA (Table 1).

We focused on the major primary degradants, which are usually those that occur in at least 10% of the total degradation [26]. For that reason, the amounts of the degradation products in samples were divided with the amount of degraded venetoclax. For isolation and characterization, we focused on stress conditions where at least 10% of venetoclax degradation occurred in a maximum of one week. A limit of one week was chosen since degradation studies were carried out at an elevated temperature of 50 °C, which is a significantly higher temperature than the ones expected in a pharmaceutical product lifetime. Degradation rate is much faster at an elevated temperature and products noticed may be produced at a significantly lower rate at normal storage temperature, if at all. Therefore, a limit of seven days was put in place to focus on the most significant degradation products. This resulted in four key HCl degradation products marked as A1, A2, A3 and A4, and three key NaOH degradation products marked as B1, B2 and B3. We used an UHPLC method to determine the amounts of degradation products, not taking into account the difference in response factors between venetoclax and degradation products. Therefore, the amounts serve merely as an orientation and a deciding factor in selection of the degradation products of interest.

Additionally, a known metabolite of venetoclax, piperazine *N*-oxide [30], which might be a potential oxidative degradation product, was obtained from commercial sources and compared to the impurity profile obtained with oxidative stress degradation conditions.

#### 3.1.1. Degradation of Venetoclax in Acidic Conditions

**Figure 3:**
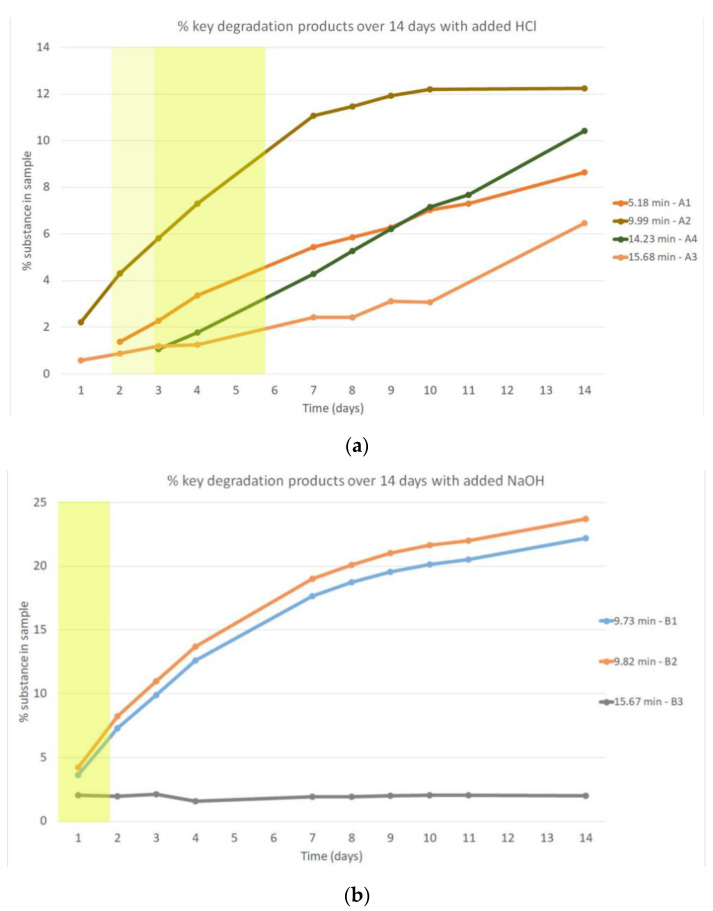
(**a**) Key degradation products of venetoclax in acidic conditions (1 M HCl, 50 °C) represented with a line chart depicting their increase in sample over time. A zone where a total degradation of 5–20% was obtained is marked with a yellow rectangle. A zone where a total degradation of 10–20% was obtained is marked with a darker yellow rectangle. (**b**) Key degradation products of venetoclax in basic conditions (1 M NaOH, 50 °C) represented with a line chart depicting their percentage in sample over time. A zone where a total degradation of 10–20% was obtained is marked with a darker yellow rectangle.

Degradation in an acidic environment using 1 M HCl occurred rapidly, achieving >5% degradation in two days and >10% degradation in three days. Four degradation products formed in an amount of at least 10% of the total degradation, where the 10–20% of total venetoclax degradation occurred (Figure 3a). This means there was at least 1% of the degradation product present in the sample (where total venetoclax degradation reached 10%) or there was at least 2% of the degradation product present in the sample (where total venetoclax degradation reached 20%). Additionally, we could notice rising and falling concentrations of certain minor degradation products (Figure S1a).

#### 3.1.2. Degradation of Venetoclax in Basic Conditions

Degradation in a basic environment using 1 M NaOH occurred the fastest, with >10% degradation (13.4%) in one day. After two days in basic conditions at 50 °C, the degradation exceeded 20%. A high number of degradation products had formed in 14 days, but mostly in low concentrations (Figure S1b). A steep rise of two degradation products B1 (*t*_R_ = 9.7 min) and B2 (*t*_R_ = 9.8 min) was observed with a steady increase over time. Another key degradation product B3 (*t*_R_ = 15.7 min) was formed in one day, the concentration of which was quite steady throughout the study, but the concentration relative to total degradation fell over time (Figure 3b).

#### 3.1.3. Degradation of Venetoclax in Oxidative Conditions

Degradation in oxidative conditions using H_2_O_2_ and ACVA did not provide 10% of venetoclax degradation in seven days. However, a rise of a potentially relevant degradation product was noticed on level of 2.57% area in UHPLC analysis with an addition of H_2_O_2_ in seven days (Figure S2), while the level of this degradation product was significantly lower in venetoclax solutions with added ACVA (0.22% area in seven days). Under oxidative conditions, venetoclax *N*-oxidation could be expected, since venetoclax contains a piperazine moiety. Such a product has been previously identified as a metabolic product [30]. Therefore, *N*-oxide venetoclax was procured from commercial sources and compared to the degradation product in venetoclax stress solution with added H_2_O_2_.

### 3.2. Characterization of Observed Key Degradation Products of Venetoclax

As was pointed out previously (see Section 3.1), we focused on stress conditions where at least 10% of total degradation occurred in a maximum of one week. This resulted in four key HCl degradation products and three key NaOH degradation products (Figure 4). The products were isolated using Flash and HPLC preparative chromatography as described in the Materials and Methods section (see Section 2.5). Their structure was determined by using NMR spectroscopy, HRMS and IR spectroscopy. In addition, the melting point of the isolated crystalline substance was determined (Table S1, Figures S3–S62, Appendix A).

**Scheme 1:**
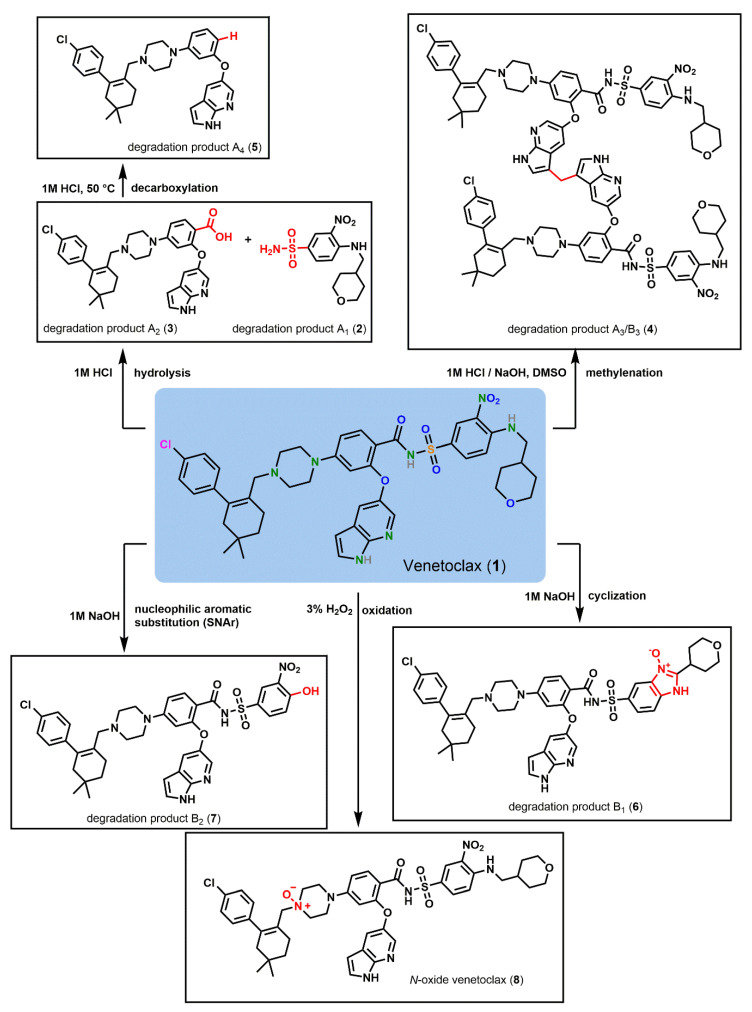
Proposed degradation pathways of venetoclax and structures of key degradation products.

The ^1^H NMR spectrum of degradation product A1 (**2**) shows NH_2_ group, one NH proton, three aromatic protons, five CH_2_ groups and one CH group; and ^13^C NMR spectrum of A1 shows six aromatic and six aliphatic carbons. A comparison of ^1^H NMR spectra of A1 and venetoclax suggests that A1 corresponds to benzenesulfonamide part of venetoclax. HRMS spectrum of A1 (Figure S17) shows a (M + H)^+^ peak at 316.0958 and a (M+Na)^+^ peak at 338.0776. (2M+Na)^+^ is also present at 653.1660. This indicates an exact mass of 315.0885, which correlates with a molecular formula of C_12_H_17_N_3_O_5_S and the proposed structure (Scheme 1, Figures S9–S17).

HRMS spectrum of degradation product A2 (**3**) shows a (M + H)^+^ peak at 571.2458, indicating an exact mass of 570.2385. The three most probable molecular formulas were C_31_H_33_ClN_7_O_2_, C_25_H_39_ClN_6_O_5_S and C_33_H_35_ClN_4_O_3_. The even nominal mass indicates an even number of nitrogen atoms. Structuring from the Cl part of the venetoclax molecule, the molecular formula C_33_H_35_ClN_4_O_3_ seemed to be most promising. The ^1^H NMR spectrum of A2 shows one NH and OH proton, 11 aromatic protons, 8 CH_2_ groups, and dimethylmethylenic group; and ^13^C NMR spectrum of A2 shows one carboxylic carbon, 19 aromatic, 2 vinylic carbons and 11 aliphatic carbons. A comparison of ^1^H NMR spectra of A2 and venetoclax suggests that benzenesulfonamide part of venetoclax (that corresponds to A1) is not present in A2. This resulted in the proposed structure of A2 (Scheme 1, Figures S18–S25).

The ^1^H and ^13^C NMR spectra of degradation product A3 (**4**) show a single set of signals that are in accordance with the proposed symmetric dimeric structure. The ^1^H NMR spectrum shows 6 NH protons, 26 aromatic protons, 27 CH_2_ groups, two CH groups and two dimethylmethylenic groups; and ^13^C NMR spectrum of A3 shows two carbonyl carbon, 50 aromatic, 4 vinylic carbons and 35 aliphatic carbons. A comparison of ^1^H NMR spectra of A3 and venetoclax showed that an additional CH_2_ group (3-CH2) is present in A3, whereas H-3 on the 1*H*-pyrrolo[2,3-*b*]pyridine ring is missing in A3. Key HMBC correlations from 3-CH_2_ to C-2, as well as from 3-CH_2_ to C-3a, confirm that 3-CH_2_ group forms a bridge between two 1*H*-pyrrolo[2,3-*b*]pyridine rings. The HRMS spectrum of A3 shows a monoisotopic (M + H)^+^ peak at 1747.6434 which indicates an exact mass of 1746.6361. This best correlates with a molecular formula of C_91_H_100_Cl_2_N_14_O_14_S_2_, which suits the structure determined by NMR. The most abundant peak in HRMS spectrum is (M+2H)^2+^ at 875.3234 which conforms with the proposed structure (Scheme 1, Figures S26–S33).

The ^1^H NMR spectrum of degradation product A4 (**5**) shows one NH and one NH^+^ proton, 12 aromatic protons, 8 CH_2_ groups and dimethylmethylenic group; and ^13^C NMR spectrum of A4 shows 19 aromatic, 2 vinylic carbons and 11 aliphatic carbons. Comparison of ^1^H NMR spectra of A4 and A3 suggests that the carboxylic group is not present in A4. This is further supported by the appearance of additional aromatic proton H-13 in ^1^H NMR spectrum of A4 as well as by the absence of carbonyl carbon C-14 in the ^13^C NMR spectrum of A4. The HRMS spectrum of A4 shows a (M + H)^+^ peak at 527.2543 and a (M+2H)^2+^ peak at 264.1307, indicating an exact mass of 526.2470. The possible molecular formulas were C_27_H_35_ClN_6_O_3_ and C_32_H_35_ClN_4_O. Judging by the number of N and O atoms in that particular part of the molecule, C_32_H_35_ClN_4_O was the most probable molecular formula, which coincided with the NMR results (Scheme 1, Figures S34–S41).

HRMS spectrum of degradation product B1 (**6**) shows a (M + H)^+^ peak at 850.3132 and a (M+2H)^2+^ at 425.6607, indicating an exact mass of 849.3059. Taking into account that the structure originates from venetoclax, the most suitable molecular formula was C_45_H_48_ClN_7_O_6_S, which indicates a loss of H_2_O from the venetoclax molecule (C_45_H_50_ClN_7_O_7_S). This was confirmed with an NMR analysis. The ^1^H NMR spectrum of B1 shows 2 NH protons, 14 aromatic protons, 12 CH_2_ groups, one CH group and dimethylmethylenic group; and ^13^C NMR spectrum of B1 shows one carbonyl carbon, 26 aromatic, 2 vinylic carbons and 16 aliphatic carbons. Comparison of ^1^H NMR spectra of B1 and venetoclax showed that NH-22 proton, as well as CH_2_-23 protons, were not present in B1. In addition, ^13^C NMR spectrum of B1 revealed that C (23) carbon is located in the aromatic region. The proposed structure was further supported by key HMBC correlation from H-25/28 to C-23, the (^1^H, ^15^N)-HMBC correlation between H-17 and N-OH nitrogen atoms, and the correlation between H-24 and N-22 nitrogen atoms (Scheme 1, Figures S42–S50).

The HRMS spectrum of degradation product B2 (**7**) shows a (M + H)^+^ peak at 771.2351, resulting in an exact mass of 770.2278. The best suited molecular formula was C_39_H_39_ClN_6_O_7_S, indicating a loss of C_6_H_11_N. Cl was still present in the molecule, which indicated changes at the other part of the venetoclax structure. A loss of tetrahydropyran moiety through amine hydrolysis would result in an exact mass of 769.2449 (C_39_H_40_ClN_7_O_6_S; -C_6_H_10_O). Proposing substitution instead of hydrolysis resulted in a structure of an exact mass of 770.2289, which correlated with the initial proposed molecular formula. The ^1^H NMR spectrum of B2 shows 2 NH protons, 14 aromatic protons, 8 CH_2_ groups and dimethylmethylenic group; and ^13^C NMR spectrum of B2 shows one carbonyl carbon, 25 aromatic, 2 vinylic carbons and 11 aliphatic carbons. The comparison of ^1^H NMR spectra of B2 and venetoclax clearly showed that tetrahydropyran ring was not present in B2 (Scheme 1, Figures S51–S58).

Degradation product B3 (**4**) was confirmed by UHPLC-UV and UHPLC-MS methods (Figures S59–S62). B3 was compared with degradation product A3 of known structure (see above). Degradation products A3 and B3 elute at the same retention time and have the same UV spectra. The MS spectra of both A3 and B3 show the (M+2H)^2+^ peak at 875. Based on these results and the behavior of the peak throughout the analytical method development, it was concluded that the structure of B3 is the same as the structure of A3.

Additionally, degradation product that occurred in the venetoclax stress sample with added H_2_O_2_ was compared to the commercially bought *N*-oxide venetoclax impurity (**8**). The *N*-oxide impurity eluted at the same retention time as the H_2_O_2_ degradation product. The compounds share the same UV and MS spectra (Figures S63–S65). The structure can be seen in Scheme 1.

### 3.3. Degradation Pathways of Venetoclax

Venetoclax has proven to be labile under acidic and basic conditions in solution (Scheme 1).

One of the key degradation pathways is *N*-acylsulfonamide moiety hydrolysis [31,32,33,34], where venetoclax degrades into a sulfonamide product, A1 (**2**), and a carboxylic acid degradation product, A2 (**3**). Based on the literature data, *N*-acylsulfonamide hydrolysis is possible in both acidic as well as alkaline conditions, but is more prevalent in acidic conditions [31]. In the case of venetoclax, we observed this degradation predominantly in acidic conditions. The carboxylic acid degradation product (**3**) can further undergo decarboxylation [35], resulting in degradation product A4 (**5**). The rate of the decarboxylation reaction usually varies depending on the pH, temperature and solvent ionic strength [36] and can occur in various solvents [37]. We observed that up to 4.3% of (**5**) was formed at 50 °C in seven days, which indicates that the decarboxylation reaction is rather slow for the venetoclax fragment (**3**) at 50 °C in DMSO/H_2_O mixture.

**Scheme 2:**
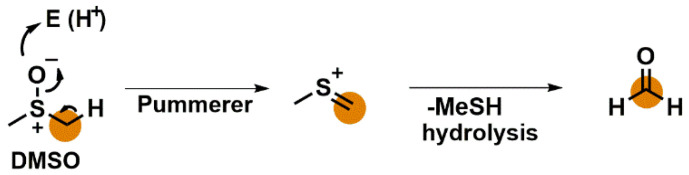
DMSO reaction resulting in a methyl(methylene)sulfonium species and formaldehyde.

Another degradation product, formed in acidic and basic conditions was degradation product A3/B3 (**4**). It is a dimer, connecting two venetoclax molecules through a methylene bridge, the origin of which is probably DMSO used in the solvent [38,39]. DMSO can undergo a Pummerer-type reaction, resulting in a methyl(methylene)sulfonium species. The hydrolysis of methyl(methylene)sulfonium species would result in formaldehyde formation (Scheme 2) [38].

**Scheme 3:**
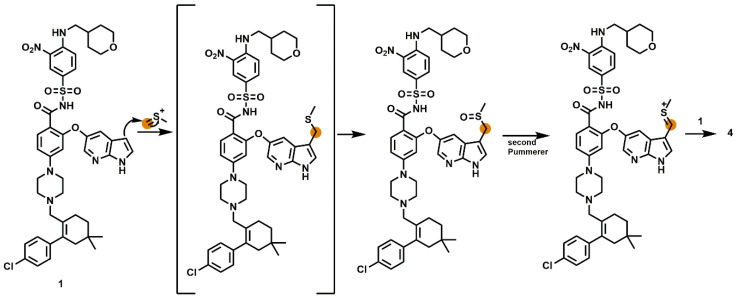
Dimerization of venetoclax through a reaction with a methyl(methylene)sulfonium species originating from DMSO.

**Scheme 4:**
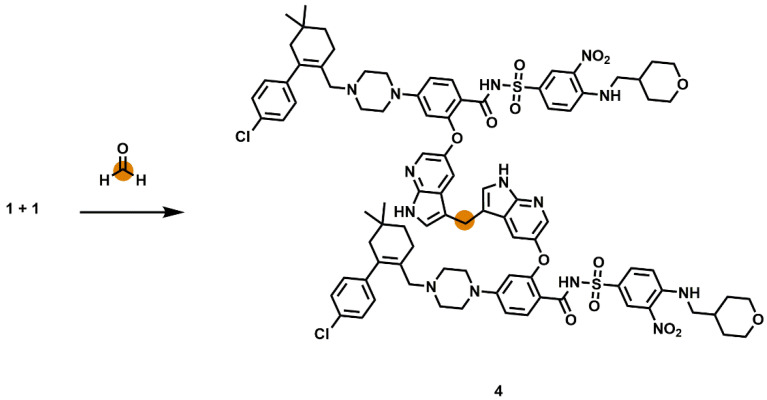
Dimerization of venetoclax with formaldehyde originating from DMSO.

Venetoclax can react with the methyl(methylene)sulfonium species, after which a second Pummerer-type reaction and a subsequent reaction with another venetoclax molecule would result in the dimer (**4**) (Scheme 3). Alternatively, venetoclax could dimerize through a reaction with formaldehyde (Scheme 4) [38,39,40]. A similar, solvent free reaction with formaldehyde was observed with an addition of a Lewis base, calcium oxide [41]. The same product could be expected in the presence of MeOH [42].

Most synthetic processes for venetoclax are done in part in the presence of DMSO and/or MeOH, so some residual solvents can be expected in the drug substance [43]. The degradation product A3 (**4**) can therefore form, though this is likely to occur in lower concentrations as a consequence of the presence of residual solvents in venetoclax. In addition, various dimers could form in a similar way, connecting venetoclax molecules with other degradation products or connecting degradation products among themselves. Degradation product A3 (**4**) can be expected in higher concentrations, compared to other dimers that could form in a similar manner, since venetoclax concentration is much higher than the concentration of its degradants. The observation of this degradation pathway is of paramount importance for design of solid dosage forms containing venetoclax. It implies that excipients based on polyether compounds such as polyethylene glycol (PEG), polyethylene oxide (PEO) and poloxamer should be avoided in excipient selection, since polyether compounds are susceptible to degradation by molecular oxygen and form small molecular weight organic impurities such as formaldehyde to a high extent [44,45,46]. Therefore, the use of polyether compounds together with venetoclax in the final dosage form could lead to the formation of dimeric degradation products.

**Scheme 5:**
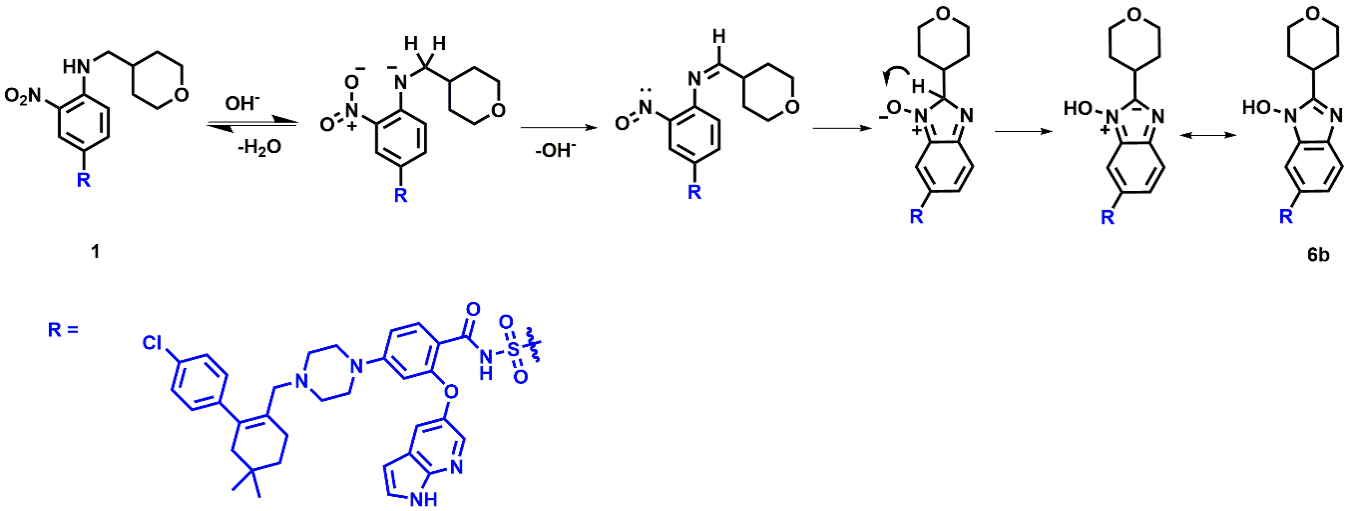
Mechanism of cyclization that results in formation of degradation product B1.

**Scheme 6:**
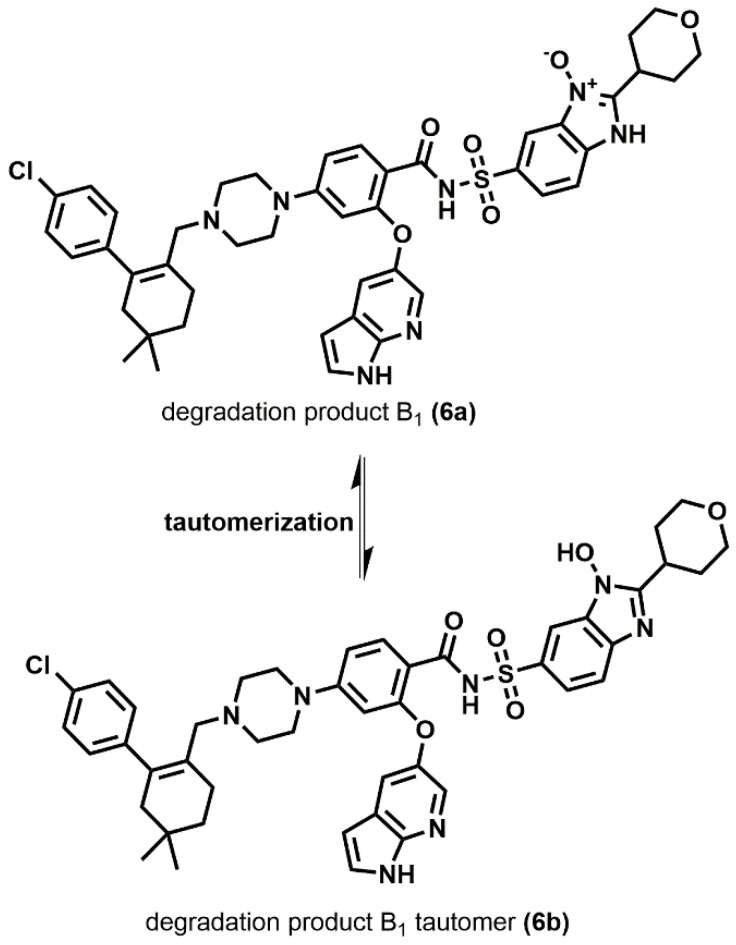
Tautomerization of degradation product B1.

In addition to dimerization, two more key degradation pathways were discovered in basic conditions. The first key degradation pathway in basic conditions is the cyclization of a *o*-nitroaniline moiety, which produces benzoimidazole *N*-oxide, degradation product B1 (**6**) [47,48,49,50,51,52,53,54,55,56,57]. The proposed mechanism for cyclization can be seen in Scheme 5. First, the nitrogen atom from the amino group is deprotonated by a hydroxide anion and water is eliminated. Lastly, cyclization involving the nitroso group and azomethine moiety occurs [51,54,56]. The product can form a tautomer (Scheme 6) [49,54]. The reaction is likely solvent-dependent. The reaction mechanism was previously observed with the addition of various organic solvents such as 1,4-dioxane [51,52,53], alcohols [53,54], DMF or DMSO [53,54]. Similar cyclization was observed under photolytic conditions on other molecules [47], therefore, in the case of venetoclax, this product could also form when exposed to photolytic conditions.

The second key degradation pathway in basic conditions is a nucleophilic aromatic substitution (S_N_Ar) of the 2-nitro-substituted benzene with the hydroxyl group from NaOH [50,54,58,59,60], resulting in degradation product B2 (**7**).

## 4. Conclusions

Forced degradation studies of venetoclax were performed under various conditions (acidic, basic, oxidative and thermolitic), revealing that venetoclax is sensitive to acidic and basic conditions in solution under an elevated temperature. For the first time, six major degradation products were isolated and their structure was determined using NMR spectroscopy, HRMS and IR spectroscopy. Major degradation pathways were proposed. Gained novel knowledge on degradation pathways of venetoclax under stress conditions can aid in future final dosage form design and studies involving venetoclax. Additionally, the degradation pathways can be applied to compounds containing the same functional groups in their molecular structures.

## Figures and Tables

**Figure 4:**
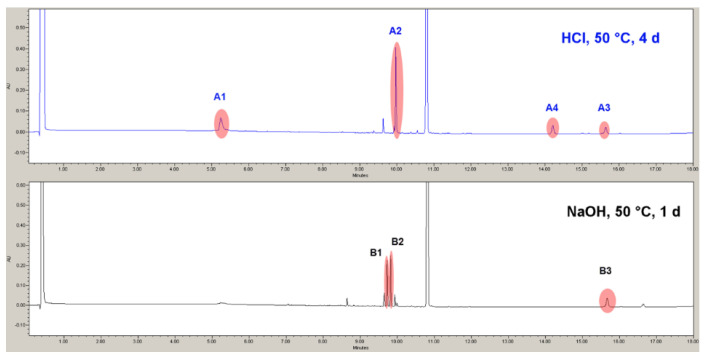
Chromatograms of venetoclax stress samples with added HCl (top) or NaOH (bottom) where 10–20% of total degradation occurred. Selected potential primary degradation products are marked with red ovals and labelled A1–A4 for acid degradation products and B1–B3 for base degradation products. For chromatographic conditions, see Section 2.3. UHPLC Method Conditions.

**Table 1:** Representation of conditions where degradation thresholds were achieved.

Stress Condition	5% Degradation	10% Degradation	20% Degradation
1 M HCl, 50 °C	2 days	3 days	7 days
1 M NaOH, 50 °C	1 day	1 day	2 days
3% H_2_O_2_, 50 °C	7 days	not achieved	not achieved
ACVA, 50 °C	2 days	14 days	not achieved

## Supplementary Materials

The following are available online at https://www.mdpi.com/1999-4923/12/7/639/s1, Figure S1: Line charts depicting the percentage of degradation products in 14 day degradation testing, Figure S2: Chromatogram of venetoclax stress sample with added H_2_O_2_, Figures S3–S8: NMR, IR and HRMS of venetoclax, Figures S9–S58: NMR, IR and HRMS of degradation products A1, A2, A3/B3, A4, B1 and B2, Figures S59–S62: Degradation product B3 information—comparison of A3 and B3, Figures S63–S65: Degradation product *N*-oxide information—comparison with a commercially bought *N*-oxide venetoclax impurity, Table S1: ^13^C and ^1^H NMR spectroscopic data for venetoclax and its degradants.

## Appendix A. Identification of Degradation Products

### Appendix A.1. Degradation Product A1

3-nitro-4-(((tetrahydro-2*H*-pyran-4-yl)methyl)amino)benzenesulfonamide (**2**): crystalline solid. Mp: onset 190.3 °C, peak 191.0 °C. ^1^H NMR (500 MHz, DMSO-*d_6_*): *δ* (ppm) 8.75 (t, *J* = 6.0 Hz, 1H), 8.47 (d, *J* = 2.3 Hz, 1H), 7.82 (dd, *J* = 2.3, 9.2 Hz, 1H), 7.32 (br s, 2H), 7.30 (d, *J* = 9.2 Hz, 1H), 3.85 (m, 2H), 3.35 (m, 2H), 3.26 (m, 2H), 1.90 (m, 1H), 1.61 (m, 2H), 1.26 (m, 2H); ^13^C NMR (125 MHz, DMSO-*d_6_*): *δ* (ppm) 146.7, 132.7, 130.0, 129.4, 124.7, 115.7, 66.6, 47.8, 33.9, 30.1; IR (KBr, cm^−1^): 3371, 3311, 3223, 2959, 2923, 2850, 1619, 1569, 1518, 1356, 1339, 1319, 1216, 1167; HRMS (ESI): *m*/*z* (M + H)^+^ calculated for C_12_H_17_N_3_O_5_S: 316.0962; found: 316.0958.

### Appendix A.2. Degradation Product A2

2-((1*H*-pyrrolo[2,3-*b*]pyridin-5-yl)oxy)-4-(4-((4′-chloro-5,5-dimethyl-3,4,5,6-tetrahydro-[1,1′-biphenyl]-2-yl)methyl)piperazin-1-yl)benzoic acid (**3**): amorphous solid; ^1^H NMR (500 MHz, DMSO-*d_6_*): *δ* (ppm) 11.64 (s, 1H), 9.34 (br s, 1H), 7.98 (d, *J* = 2.6 Hz, 1H), 7.78 (d, *J* = 8.9, 1H), 7.47 (m, 1H), 7.38–7.41 (m, 3H), 7.09 (m, 2H), 6.77 (dd, *J* = 2.5, 8.9 Hz, 1H), 6.41 (d, *J* = 2.5, 1H), 6.38 (dd, *J* = 1.9, 3.3 Hz, 1H), 3.74 (br s, 2H), 3.59 (s, 2H), 3.28 (br s, 2H), 3.06 (br s, 2H), 2.78 (br s, 2H), 2.20 (br m, 2H), 2.02 (s, 2H), 1.46 (m, 2H), 0.95 (s, 6H); ^13^C NMR (125 MHz, DMSO-*d_6_*): *δ* (ppm) 165.9, 158.4, 153.4, 148.3, 144.9, 141.7, 140.4, 134.6, 133.5, 131.8, 129.8, 128.7, 127.6, 121.7, 119.8, 116.3, 113.0, 109.5, 105.6, 99.9, 58.0, 50.6, 46.6, 43.8, 34.3, 28.7, 27.9, 24.8; IR (KBr, cm^−1^): 3600–2400 (stretch), 2957, 2925, 2868, 1675, 1610, 1489, 1432, 1401, 1201, 1136, 832, 721; HRMS (ESI): *m*/*z* (M + H)^+^ calculated for C_33_H_35_ClN_4_O_3_: 571.2470; found: 571.2458.

### Appendix A.3. Degradation Product A3/B3

2,2′-((methylenebis(1*H*-pyrrolo[2,3-*b*]pyridine-1,5-diyl))bis(oxy))bis(4-(4-((4′-chloro-5,5-dimethyl-3,4,5,6-tetrahydro-[1,1′-biphenyl]-2-yl)methyl)piperazin-1-yl)-*N*-((3-nitro-4-(((tetrahydro-2*H*-pyran-4-yl)methyl)amino)phenyl)sulfonyl)benzamide) (**4**): amorphous solid; ^1^H NMR (500 MHz, DMSO-*d_6_*): *δ* (ppm) 11.64 (br s, 2H), 11.43 (d, *J* = 2.5 Hz, 2H), 8.62 (t, *J* = 6.0 Hz, 2H), 8.58 (d, *J* = 2.3 Hz, 2H), 7.97 (d, *J* = 2.6 Hz, 2H), 7.84 (dd, *J* = 2.3, 9.4 Hz, 2H), 7.66 (d, *J* = 2.6 Hz, 2H), 7.51 (d, *J* = 9.0 Hz, 2H), 7.37 (m, 4H), 7.34 (d, *J* = 2.5 Hz, 2H), 7.13 (d, *J* = 9.4 Hz, 2H), 7.07 (m, 4H), 6.69 (dd, *J* = 2.3, 9.0 Hz, 2H), 6.19 (d, *J* = 2.3 Hz, 2H), 4.03 (s, 2H), 3.82 (m, 4H), 3.62 (br s, 4H), 3.56 (s, 4H), 3.28 (m, 4H), 3.23 (m, 4H), 3.20 (br s, 4H), 3.00 (br s, 4H), 2.74 (br s, 4H), 2.18 (br m, 4H), 2.00 (s, 4H), 1.86 (m, 2H), 1.58 (m, 4H), 1.44 (m, 4H), 1.23 (m, 4H), 0.93 (s, 12H); ^13^C NMR (125 MHz, DMSO-*d_6_*): *δ* (ppm) 163.5, 158.1, 153.5, 147.5, 145.9, 145.5, 141.6 (br s), 140.3, 135.2, 133.9, 132.2, 131.8, 129.8, 129.6, 128.7, 127.9, 125.3, 124.3, 121.8 (br s), 119.5, 117.3, 115.1, 113.2, 112.6, 109.0, 102.3, 66.6, 58.1, 50.5, 47.9, 46.6, 43.8, 34.2, 33.9, 30.1, 28.7, 27.8, 24.8, 20.8; IR (KBr, cm^−1^): 3600–3000 (stretch), 2925, 2854, 1677, 1615, 1521, 1487, 1433, 1347, 1267, 1241, 1201, 1173, 1134, 1104, 834, 721; HRMS (ESI): *m*/*z* (M + H)^+^ calculated for C_91_H_100_Cl_2_N_14_O_14_S_2_: 1747.6435; found: 1747.6434.

### Appendix A.4. Degradation Product A4

5-(3-(4-((4′-chloro-5,5-dimethyl-3,4,5,6-tetrahydro-[1,1′-biphenyl]-2-yl)methyl)piperazin-1-yl)phenoxy)-1*H*-pyrrolo[2,3-b]pyridine (**5**): ^1^H NMR (500 MHz, DMSO-*d_6_*): *δ* (ppm) 11.72 (s, 1H), 9.25 (br s, 1H), 8.03 (d, *J* = 2.6 Hz, 1H), 7.63 (dd, *J* = 0.5, 2.6 Hz, 1H), 7.52 (m, 1H), 7.43 (m, 2H), 7.18 (m, 1H), 7.13 (m, 2H), 6.65 (dd, *J* = 2.0, 8.1 Hz, 1H), 6.57 (m, 1H), 6.42 (dd, *J* = 1.9, 3.4 Hz, 1H), 6.34 (dd, *J* = 2.0, 8.1 Hz, 1H), 3.65 (br m, 2H), 3.64 (s, 2H), 3.33 (br m, 2H), 3.01 (br m, 2H), 2.83 (br m, 2H), 2.23 (br m, 2H), 2.05 (s, 2H), 1.49 (t, *J* = 6.3 Hz, 2H), 0.97 (s, 6H); ^13^C NMR (125 MHz, DMSO-*d_6_*): *δ* (ppm)159.7, 151.0, 146.4, 145.5, 141.7, 140.3, 135.8, 131.8, 130.3, 129.8, 128.7, 127.7, 121.7, 120.0, 118.8, 110.0, 108.1, 104.7, 99.9, 57.9, 50.8, 46.5, 45.0, 34.2, 28.7, 27.8, 24.8; IR (KBr, cm^−1^): 3600–3000 (stretch), 2959, 2926, 2869, 1676, 1606, 1489, 1457, 1402, 1334, 1250, 1203, 1137, 1091, 1048, 834, 722; HRMS (ESI): *m*/*z* (M + H)^+^ calculated for C_32_H_35_ClN_4_O: 527.2572; found: 527.2543.

### Appendix A.5. Degradation Product B1

5-(*N*-(2-((1*H*-pyrrolo[2,3-*b*]pyridin-5-yl)oxy)-4-(4-((4′-chloro-5,5-dimethyl-3,4,5,6-tetrahydro-[1,1′-biphenyl]-2-yl)methyl)piperazin-1-yl)benzoyl)sulfamoyl)-2-(tetrahydro-2*H*-pyran-4-yl)-1*H*-benzo[d]imidazole 3-oxide (**6a**); tautomer: 2-((1*H*-pyrrolo[2,3-*b*]pyridin-5-yl)oxy)-4-(4-((4′-chloro-5,5-dimethyl-3,4,5,6-tetrahydro-[1,1′-biphenyl]-2-yl)methyl)piperazin-1-yl)-*N*-((1-hydroxy-2-(tetrahydro-2*H*-pyran-4-yl)-1*H*-benzo[d]imidazol-6-yl)sulfonyl)benzamide (**6b**): amorphous solid; ^1^H NMR (500 MHz, DMSO-*d_6_*): *δ* (ppm) 11.77 (s, 1H), 11.68 (s, 1H), 8.07–8.08 (m, 2H), 7.67 (m, 2H), 7.62 (d, *J* = 2.5 Hz, 1H), 7.54 (m, 1H), 7.47 (d, *J* = 8.9 Hz, 1H), 7.38 (m, 2H), 7.07 (m, 2H), 6.68 (dd, *J* = 2.3, 8.9 Hz, 1H), 6.43 (dd, *J* = 1.9, 3.4 Hz, 1H), 6.22 (d, *J* = 2.3 Hz, 1H), 3.96 (m, 2H), 3.64 (br s, 2H), 3.56 (s, 2H), 3.50 (m, 2H), 3.37 (m, 1H), 3.26 (br s, 2H), 3.02 (br s, 2H), 2.74 (br s, 2H), 2.18 (br m, 2H), 2.00 (s, 2H), 1.82–1.90 (m, 4H), 1.44 (m, 2H), 0.93 (s, 6H); ^13^C NMR (125 MHz, DMSO-*d_6_*): *δ* (ppm) 163.3, 158.1, 157.1, 153.5, 146.1, 145.6, 141.6, 140.3, 135.4, 132.9, 132.1, 131.7, 131.1, 129.7, 128.7, 127.9, 121.7, 120.6, 119.9, 119.1, 118.5, 113.5, 110.0, 109.0, 102.6, 100.1, 66.5, 58.0, 50.5, 46.6, 43.8, 34.2, 31.9, 29.9, 28.6, 27.8, 24.7; IR (KBr, cm^−1^): 3600–2400 (stretch), 3311, 2958, 2926, 2860, 1675, 1609, 1533, 1432, 1405, 1349, 1261, 1202, 1171, 1137, 1103, 1070, 831, 722; HRMS (ESI): *m*/*z* (M + H)^+^ calculated for C_45_H_48_ClN_7_O_6_S: 850.3148; found: 850.3132.

### Appendix A.6. Degradation Product B2

2-((1*H*-pyrrolo[2,3-*b*]pyridin-5-yl)oxy)-4-(4-((4′-chloro-5,5-dimethyl-3,4,5,6-tetrahydro-[1,1′-biphenyl]-2-yl)methyl)piperazin-1-yl)-*N*-((4-hydroxy-3-nitrophenyl)sulfonyl)benzamide (**7**): ^1^H NMR (500 MHz, DMSO-*d_6_*): *δ* (ppm) 11.77 (br s, 1H), 11.74 (s, 1H), 8.39 (d, *J* = 2.4 Hz, 1H), 8.06 (d, *J* = 2.6 Hz, 1H), 7.98 (dd, *J* = 2.4, 8.9 Hz, 1H), 7.60 (d, *J* = 2.6 Hz, 1H), 7.50–7.53 (m, 2H), 7.39 (m, 2H), 7.23 (d, *J* = 8.9 Hz, 1H), 7.07 (m, 2H), 6.71 (dd, *J* = 2.4, 9.0 Hz, 1H), 6.4dei2 (dd, *J* = 1.9, 3.4 Hz, 1H), 6.23 (d, *J* = 2.4 Hz, 1H), 3.64 (br s, 2H), 3.57 (s, 2H), 3.25 (br s, 2H), 3.01 (br s, 2H), 2.75 (br s, 2H), 2.17 (br m, 2H), 2.00 (s, 2H), 1.44 (m, 2H), 0.94 (s, 6H); ^13^C NMR (125 MHz, DMSO-*d_6_*): *δ* (ppm) 163.5, 158.1, 156.1, 153.6, 146.1, 145.6, 141.6, 140.3, 136.2, 135.4, 133.9, 132.3, 131.8, 129.8, 129.3, 128.7, 127.9, 126.1, 121.7, 119.9, 119.6, 118.3, 113.2, 109.1, 102.6, 100.0, 58.0, 50.5, 46.6, 43.8, 34.2, 28.7, 27.8, 24.8; IR (KBr, cm^−1^): 3600–3000 (stretch), 2956, 2924, 2867, 1675, 1609, 1533, 1488, 1432, 1405, 1349, 1261, 1203, 1171, 1137, 1103, 1070, 905, 831, 722; HRMS (ESI): *m*/*z* (M + H)^+^ calculated for C_39_H_39_ClN_6_O_7_S: 771.2362; found: 771.2351.
