# Supplementary material for: On the Stability and Degradation Pathways of Venetoclax under Stress Conditions
Source: Pharmaceutics. 2020 Jul 7;12(7):639. doi: 10.3390/pharmaceutics12070639 (PMC7407384; doi:10.3390/pharmaceutics12070639)
Supplement: Supplementary file 1 [file pharmaceutics-12-00639-s001.pdf]

Supplementary Materials: On the Stability and Degradation Pathways of Venetoclax under Stress Conditions

Nina Žigart, Martin Črnugelj, Janez Ilaš and Zdenko Časar

List of supplementary material

Page	Content
3	**Figure S1** – Line charts depicting the percentage of degradation products in 14 day degradation testing
4	**Figure S2** – Chromatogram of the venetoclax stress sample with added 3% H_2O_2 at 50 °C for 7 days
5-7	**Table S1** – ^{13}C and ^{1}H NMR spectroscopic data for venetoclax and its degradants
8-13	**Venetoclax information**
8	**Figure S3** – Venetoclax with NMR assignations
9	**Figure S4** – ^{1}H NMR spectrum of venetoclax
10	**Figure S5** – ^{13}C NMR spectrum of venetoclax
11	**Figure S6** – (^{1}H, ^{1}H)-COSY spectrum of venetoclax
12	**Figure S7** – (^{1}H, ^{13}C)-HSQC spectrum of venetoclax
13	**Figure S8** – (^{1}H, ^{13}C)-HMBC spectrum of venetoclax
14-22	**Degradation product A1 information**
14	**Figure S9** – Degradation product A1 with NMR assignations
15	**Figure S10** – ^{1}H NMR spectrum of A1
16	**Figure S11** – ^{13}C NMR spectrum of A1
17	**Figure S12** – (^{1}H, ^{1}H)-COSY spectrum of A1
18	**Figure S13** – (^{1}H, ^{13}C)-HSQC spectrum of A1
19	**Figure S14** – (^{1}H, ^{13}C)-HMBC spectrum of A1
20	**Figure S15** – IR spectrum of A1
21	**Figure S16** – DSC curve for A1
22	**Figure S17** – HRMS spectrum of A1
23-30	**Degradation product A2 information**
23	**Figure S18** – Degradation product A2 with NMR assignations
24	**Figure S19** – ^{1}H NMR spectrum of A2
25	**Figure S20** – ^{13}C NMR spectrum of A2
26	**Figure S21** – (^{1}H, ^{1}H)-COSY spectrum of A2
27	**Figure S22** – (^{1}H, ^{13}C)-HSQC spectrum of A2
28	**Figure S23** – (^{1}H, ^{13}C)-HMBC spectrum of A2
29	**Figure S24** – IR spectrum of A2
30	**Figure S25** – HRMS spectrum of A2
31-38	**Degradation product A3/B3 information**
31	**Figure S26** – Degradation product A3 with NMR assignations
32	**Figure S27** – ^{1}H NMR spectrum of A3
33	**Figure S28** – ^{13}C NMR spectrum of A3

34	**Figure S29 –** (^{1}H, ^{1}H)-COSY spectrum of A3
35	**Figure S30 –** (^{1}H, ^{13}C)-HSQC spectrum of A3
36	**Figure S31 –** (^{1}H, ^{13}C)-HMBC spectrum of A3
37	**Figure S32 –** IR spectrum of A3
38	**Figure S33 –** HRMS spectrum of A3
39-46	**Degradation product A4 information**
39	**Figure S34 –** Degradation product A4 with NMR assignations
40	**Figure S35 –** ^{1}H NMR spectrum of A4
41	**Figure S36 –** ^{13}C NMR spectrum of A4
42	**Figure S37 –** (^{1}H, ^{1}H)-COSY spectrum of A4
43	**Figure S38 –** (^{1}H, ^{13}C)-HSQC spectrum of A4
44	**Figure S39 –** (^{1}H, ^{13}C)-HMBC spectrum of A4
45	**Figure S40 –** IR spectrum of A4
46	**Figure S41 –** HRMS spectrum of A4
47-55	**Degradation product B1 information**
47	**Figure S42 –** Degradation product B1 with NMR assignations
48	**Figure S43 –** ^{1}H NMR spectrum of B1
49	**Figure S44 –** ^{13}C NMR spectrum of B1
50	**Figure S45 –** (^{1}H, ^{1}H)-COSY spectrum of B1
51	**Figure S46 –** (^{1}H, ^{13}C)-HSQC spectrum of B1
52	**Figure S47 –** (^{1}H, ^{13}C)-HMBC spectrum of B1
53	**Figure S48 –** (^{1}H, ^{15}N)-HMBC spectrum of B1
54	**Figure S49 –** IR spectrum of B1
55	**Figure S50 –** HRMS spectrum of B1
56-63	**Degradation product B2 information**
56	**Figure S51 –** Degradation product B2 with NMR assignations
57	**Figure S52 –** ^{1}H NMR spectrum of B2
58	**Figure S53 –** ^{13}C NMR spectrum of B2
59	**Figure S54 –** (^{1}H, ^{1}H)-COSY spectrum of B2
60	**Figure S55 –** (^{1}H, ^{13}C)-HSQC spectrum of B2
61	**Figure S56 –** (^{1}H, ^{13}C)-HMBC spectrum of B2
62	**Figure S57 –** IR spectrum of B2
63	**Figure S58 –** HRMS spectrum of B2
64-67	**Degradation product B3 information**
64	**Figure S59 –** Overlay chromatogram of a stress sample of venetoclax with added 1M NaOH after 1 day at 50 °C and degradation product A3
65	**Figure S60 –** Chromatograms and UV spectra of degradation product B3 and degradation product A3
66	**Figure S61 –** Chromatograms of a stress sample of venetoclax with added 1M NaOH after 1 day at 50 °C and degradation product A3 obtained with UV and MS detector
67	**Figure S62 –** MS spectra of degradation product A3 and degradation product B3
68-70	**Degradation product *N*-oxide information**
68	**Figure S63 –** Overlay chromatogram of a stress sample of venetoclax with added 3% H_2O_2 after 7 days at 50 °C and commercially obtained *N*-oxide venetoclax impurity
69	**Figure S64 –** Chromatograms and UV spectra of degradation product *N*-oxide in stress sample of venetoclax with added H_2O_2 and commercially obtained *N*-oxide venetoclax
70	**Figure S65 -** MS spectra of a commercially obtained *N*-oxide venetoclax and degradation product *N*-oxide in stress sample of venetoclax with added H_2O_2

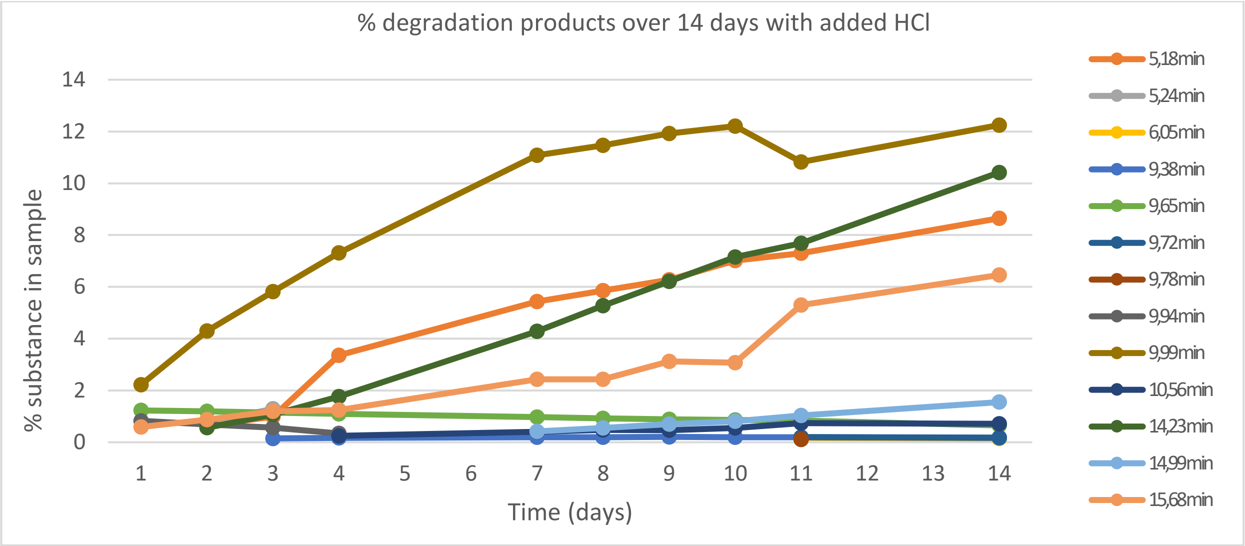

(a)

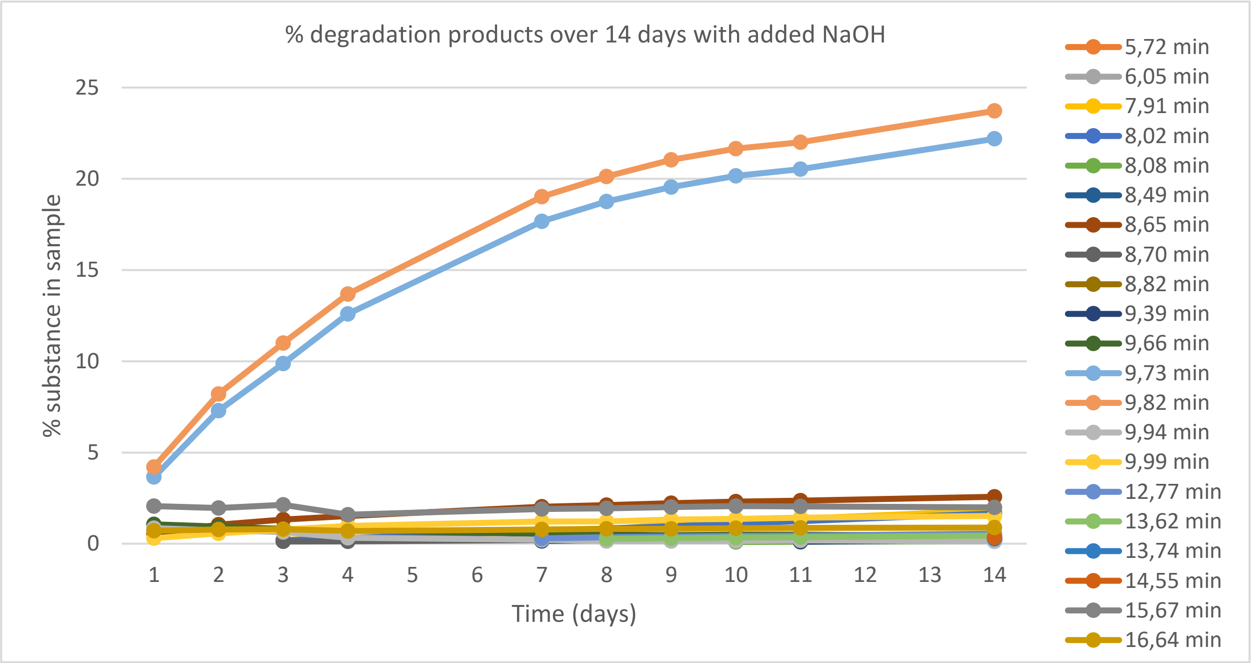

(b)

Figure S1. – Line charts depicting the percentage of degradation products in 14 day degradation testing. a) Line chart representing the percentage of degradation products in the venetoclax stress sample with added HCl (1 M) as a stress medium at 50 °C. Degradation products are named by their approximate retention times.

b) Line chart representing the percentage of degradation products in the venetoclax stress sample with added NaOH (1 M) as a stress medium at 50 °C. Degradation products are named by their approximate retention times.

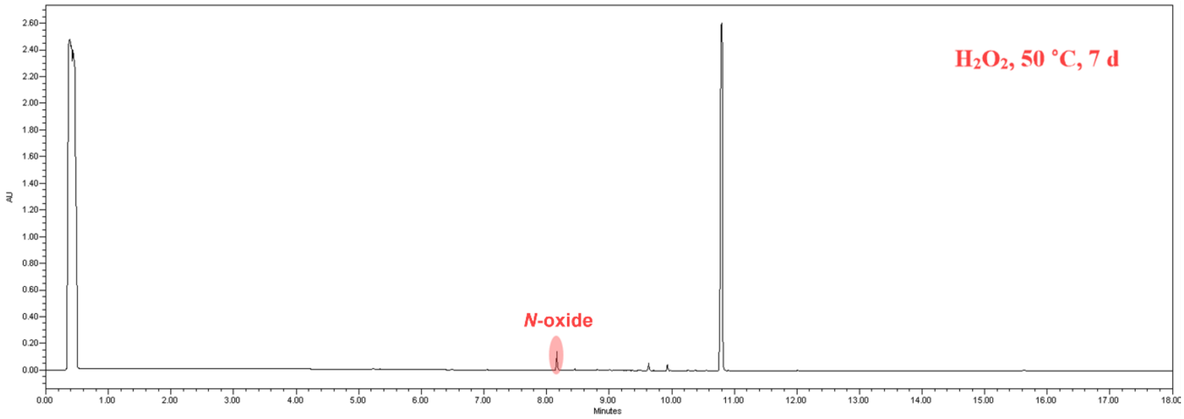

Figure S2. – Chromatogram of the venetoclax stress sample with added 3% H_2O_2 at 50 °C for 7 days. The rise of one degradation product was noticeable, which was later identified as an *N*-oxide venetoclax, where the piperazine moiety is oxidized.

Table S1 – ^{13}C and ^{1}H NMR spectroscopic data for venetoclax and its degradants (125 and 500 MHz, in DMSO-d_6). Chemical shifts (δ) are expressed in ppm with reference to residual solvent signal (2.50 ppm and 39.5 ppm for ^{1}H and ^{13}C, respectively).

Atom No.	Venetoclax		VENE-B1		VENE-B2		VENE-A1		VENE-A2		VENE-A3		VENE-A4	
	^{13}C shift [ppm] & peak multiplicity	^{1}H shift [ppm], peak multiplicity & coupling constants [*J*]	^{13}C shift [ppm] & peak multiplicity	^{1}H shift [ppm], peak multiplicity & coupling constants [*J*]	^{13}C shift [ppm] & peak multiplicity	^{1}H shift [ppm], peak multiplicity & coupling constants [*J*]	^{13}C shift [ppm] & peak multiplicity	^{1}H shift [ppm], peak multiplicity & coupling constants [*J*]	^{13}C shift [ppm] & peak multiplicity	^{1}H shift [ppm], peak multiplicity & coupling constants [*J*]	^{13}C shift [ppm] & peak multiplicity	^{1}H shift [ppm], peak multiplicity & coupling constants [*J*]	^{13}C shift [ppm] & peak multiplicity	^{1}H shift [ppm], peak multiplicity & coupling constants [*J*]
2	127.7 s	7.49 – 7.51 m	127.9 s	7.54 m	127.9 s	7.50 – 7.53 m			127.6 s	7.47 m	125.3 s	7.34 (d) 2.5	127.7 s	7.52 m
3	100.0 s	6.38 (dd) 1.8, 3.0	100.1 s	6.43 (dd) 1.9, 3.4	100.0 s	6.42 (dd) 1.9, 3.4			99.9 s	6.38 (dd) 1.9, 3.3	112.6 s		99.9 s	6.42 (dd) 1.9, 3.4
3a	119.8 s		119.9 s		119.9 s				119.8 s		119.5 s		120.0 s	
4	117.9 s	7.54 (d) 2.5	118.5 s	7.62 (d) 2.5	118.3 s	7.60 (d) 2.6			116.3 s	7.38 – 7.41 m	117.3 s	7.66 (d) 2.6	118.8 s	7.63 (dd) 0.5, 2.6
5	146.5 s		146.1 s		146.1 s				148.3 s		145.5 s		146.4 s	
6	135.3 s	8.05 (d) 2.5	135.4 s	8.07 – 8.08 m	135.4 s	8.06 (d) 2.6			134.6 s	7.98 (d) 2.6	135.2 s	7.97 (d) 2.6	135.8 s	8.03 (d) 2.6
7a	145.4 s		145.6 s		145.6 s				144.9 s		145.9 s		145.5 s	
8	157.8 s		158.1 s		158.1 s				158.4 s		158.1 s		159.7 s	
9	102.4 s	6.18 (d) 1.6	102.6 s	6.22 (d) 2.3	102.6 s	6.23 (d) 2.4			105.6 s	6.41 (d) 2.5	102.3 s	6.19 (d) 2.3	104.7 s	6.57 m
10	154.5 s		153.5 s		153.6 s				153.4 s		153.5 s		151.0 s	
11	108.7 s	6.66 (dd) 1.6, 9.2	109.0 s	6.68 (dd) 2.3, 8.9	109.1 s	6.71 (dd) 2.4, 9.0			109.5 s	6.77 (dd) 2.5, 8.9	109.0 s	6.69 (dd) 2.3, 9.0	110.0 s	6.65 (dd) 2.0, 8.1

12	132.1 s	7.49 – 7.51 m	132.1 s	7.47 (d) 8.9	132.3 s	7.50 – 7.53 m			133.5 s	7.78 (d) 8.9	132.2 s	7.51 (d) 9.0	130.3 s	7.18 m
13	112.6 s		113.5 s		113.2 s				113.0 s		113.2 s		108.1 s	6.34 (dd) 2.0, 8.1
14	163.8 s		163.3 s		163.5 s				165.9 s		163.5 s			
16	124.9 s		131.1 s		129.3 s		130.0 s				124.3 s			
17	127.7 s	8.57 (d) 2.2	110.0 s	8.07 – 8.08 m	126.1 s	8.39 (d) 2.4	124.7 s	8.47 (d) 2.3			127.9 s	8.58 (d) 2.3		
18	129.5 s		132.9 s		136.2 s		129.4 s				129.6 s			
19	147.3 s		140.3 s		156.1 s		146.7 s				147.5 s			
20	115.0 s	7.10 (d) 9.3	119.1 s	7.67 m	119.6 s	7.23 (d) 8.9	115.7 s	7.30 (d) 9.2			115.1 s	7.13 (d) 9.4		
21	133.9 s	7.81 (dd) 1.8, 9.3	120.6 s	7.67 m	133.9 s	7.98 (dd) 2.4, 8.9	132.7 s	7.82 (dd) 2.3, 9.2			133.9 s	7.84 (dd) 2.3, 9.4		
23	47.9 s	3.29 m	157.1 s				47.8 s	3.35 m			47.9 s	3.28 m		
24	33.8 s	1.88 m	31.9 s	3.37 m			33.9 s	1.90 m			33.9 s	1.86 m		
25, 28	30.2 s	1.25 m, 1.60 m	29.9 s	1.82 – 1.90 m			66.6 s	1.26 m, 1.61 m			30.1 s	1.23 m, 1.58 m		
26, 27	66.6 s	3.25 m, 3.84 m	66.5 s	3.50 m, 3.96 m			30.1 s	3.26 m, 3.85 m,			66.6 s	3.23 m, 3.82 m,		
2′, 6′	46.5 s	3.06 br.s	43.8 s	3.02 br.s, 3.64 br.s	43.8 s	3.01 br.s, 3.64 br.s				3.06 br.s, 3.74 br.s	43.8 s	3.00 br.s, 3.62 br.s	45.0 s	3.01 br.m, 3.65 br.m
3′, 5′	52.0 s	2.19 br.s	50.5 s	2.74 br.s, 3.26 br.s	50.5 s	2.75 br.s, 3.25 br.s				2.78 br.s, 3.28 br.s	50.5 s	2.74 br.s, 3.20 br.s	50.8 s	2.83 br.m, 3.33 br.m
7′	59.6 s	2.74 s	58.0 s	3.56 s	58.0 s	3.57 s			58.0 s	3.59 s	58.1 s	3.56 s	57.9 s	3.64 s
8′	128.5 s		121.7 s		121.7 s						121.8 br.s		121.7 s	
9′	134.6 s		141.6 s		141.6 s				141.7 s		141.6 br.s		141.7 s	
10′	46.3 s	1.93 s	46.6 s	2.00 s	46.6 s	2.00 s			46.6 s	2.02 s	46.6 s	2.00 s	46.5 s	2.05 s
11′	28.8 s		28.6 s		28.7 s				28.7 s		28.7 s		28.7 s	
12'	34.8 s	1.36 (t) 6.2	34.2 s	1.44 m	34.2 s	1.44 m			34.3 s	1.46 m	34.2 s	1.44 m	34.2 s	1.49 (t) 6.3
13′	25.1 s	2.12 br.m	24.7 s	2.18 br.m	24.8 s	2.17 br.m			24.8 s	2.20 br.m	24.8 s	2.18 br.m	24.8 s	2.23 br.m

1″	141.9 s		140.3 s		140.3 s				140.4 s		140.3 s		140.3 s	
2″, 6″	130.0 s	7.02 m	129.7 s	7.07 m	129.8 s	7.07 m			129.8 s	7.09 m	129.8 s	7.07 m	129.8 s	7.13 m
3″, 5″	128.1 s	7.32 m	128.7 s	7.38 m	128.7 s	7.39 m			128.7 s	7.38 – 7.41 m	128.7 s	7.37 m	128.7 s	7.43 m
4″	130.8 s		131.7 s		131.8 s				131.8 s		131.8 s		131.8 s	
3-CH_2											20.8 s	4.03 s		
11′-$(CH_3)_2$	27.9 s	0.90 s	27.8 s	0.93 s	27.8 s	0.94 s			27.9 s	0.95 s	27.8 s	0.93 s	27.8 s	0.97 s
1-NH		11.70 s		11.77 s		11.74 s				11.64 s		11.64 br.s		11.72 s
15-NH		11.32 br. s		11.68 s		11.77 br.s						11.43 (d) 2.5		
22-NH		8.61 (t) 5.8						8.75 (t) 6.0				8.62 (t) 6.0		
14 -OH										9.34 br. s				
NH^+(4′)														9.25 br.s
15-NH_2								7.32 (br.s)						

Venetoclax information

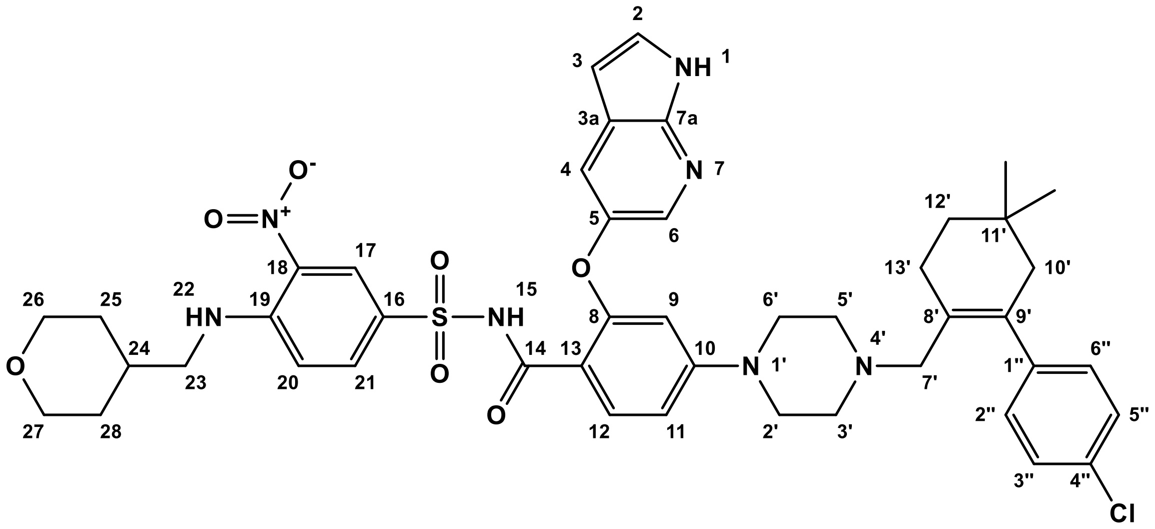

Figure S3. – Venetoclax with NMR assignations.

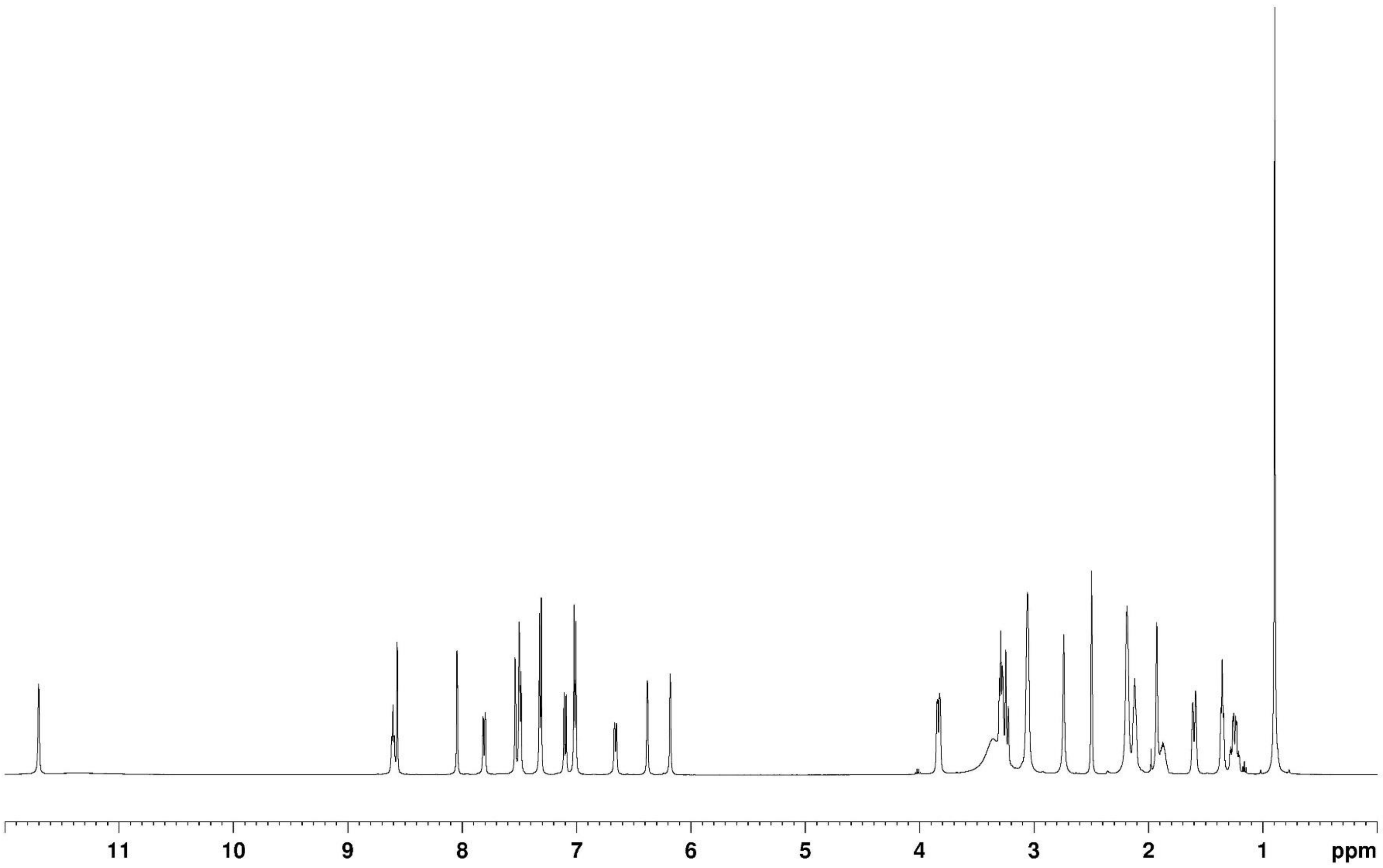

Figure S4 – ^{1}H NMR spectrum of **venetoclax**

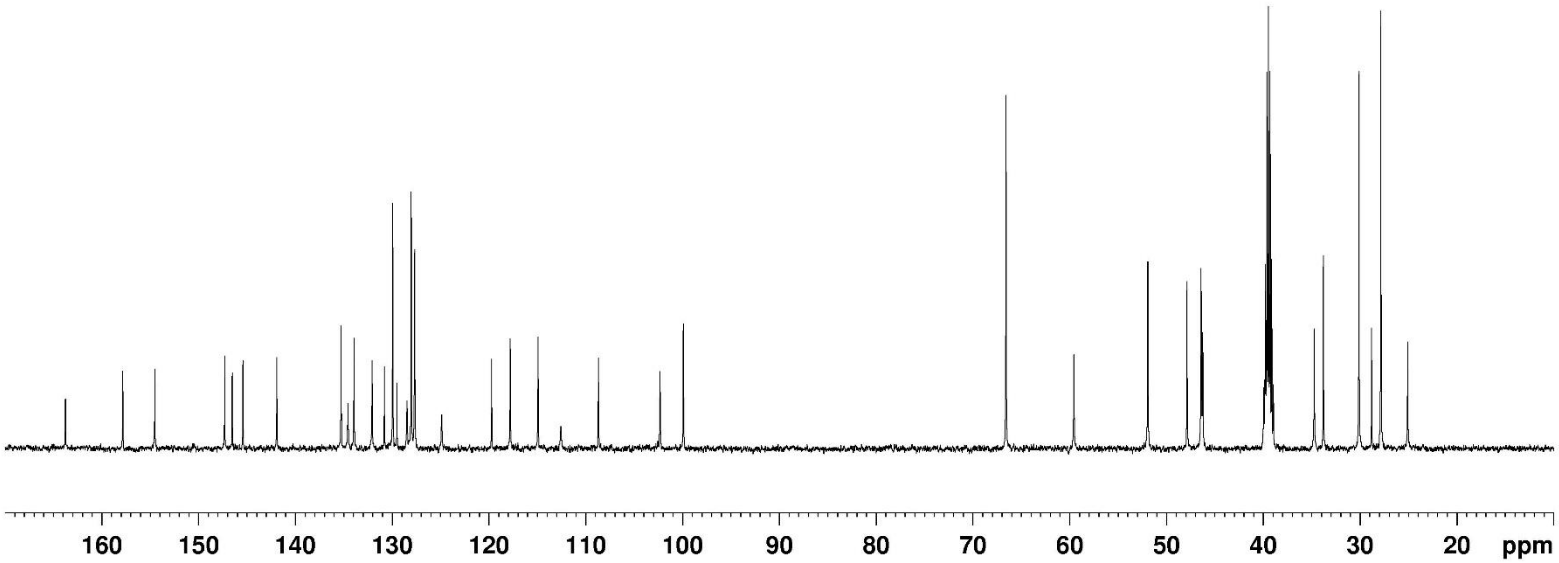

Figure S5 – ^{13}C NMR spectrum of **venetoclax**

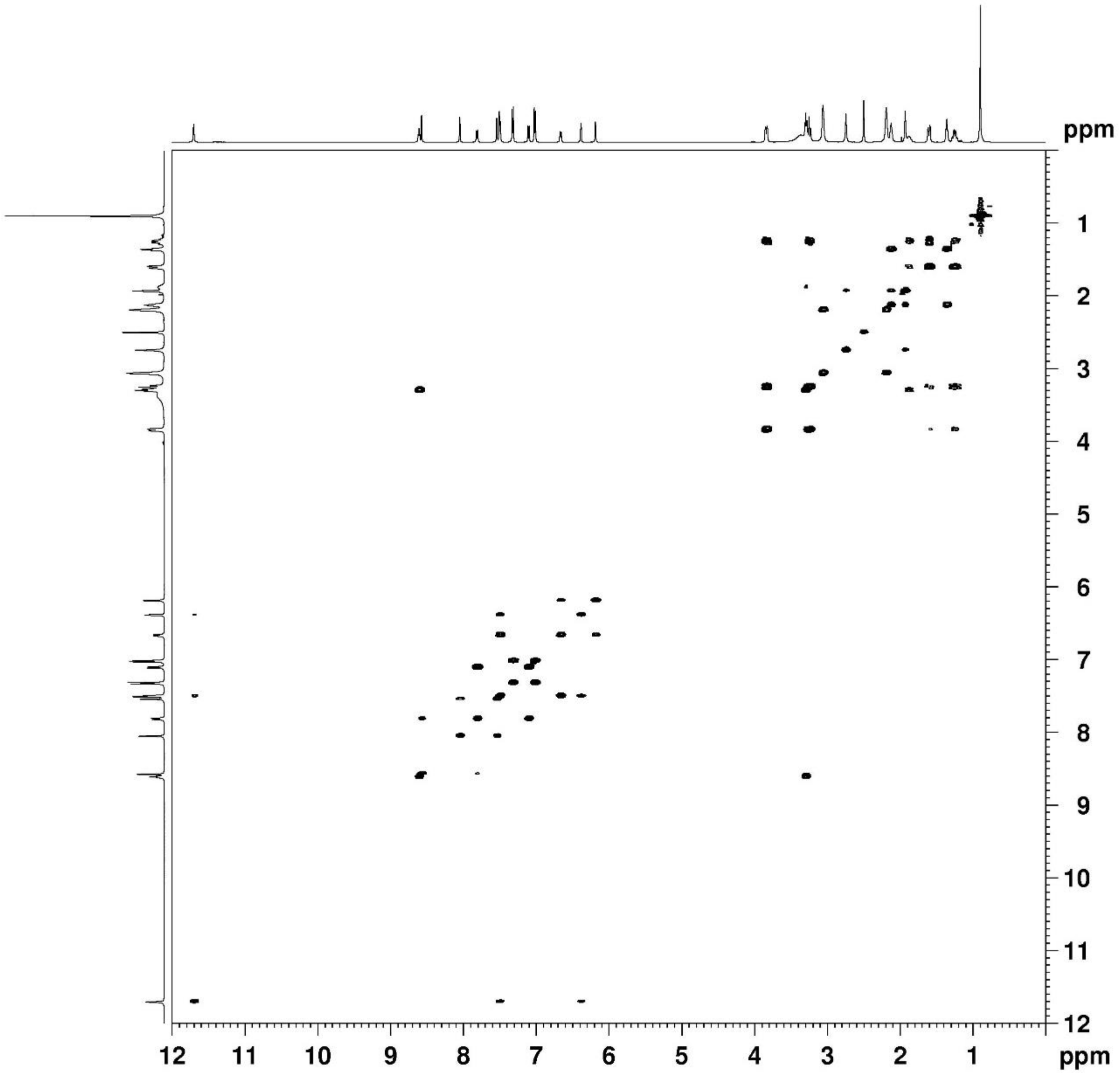

Figure S6 – (^{1}H, ^{1}H)-COSY spectrum of **venetoclax**

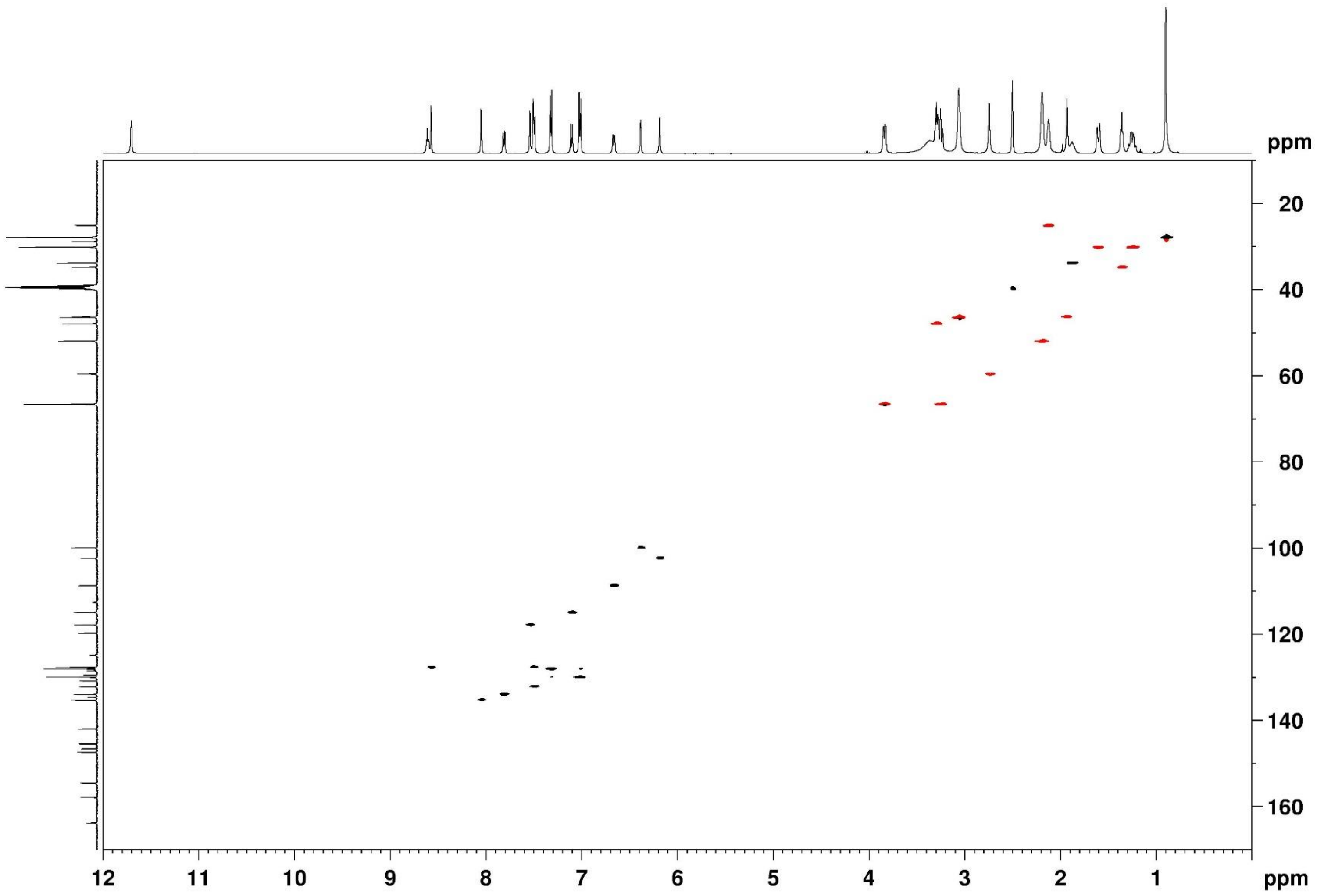

Figure S7 – (^{1}H, ^{13}C)-HSQC spectrum of **venetoclax**

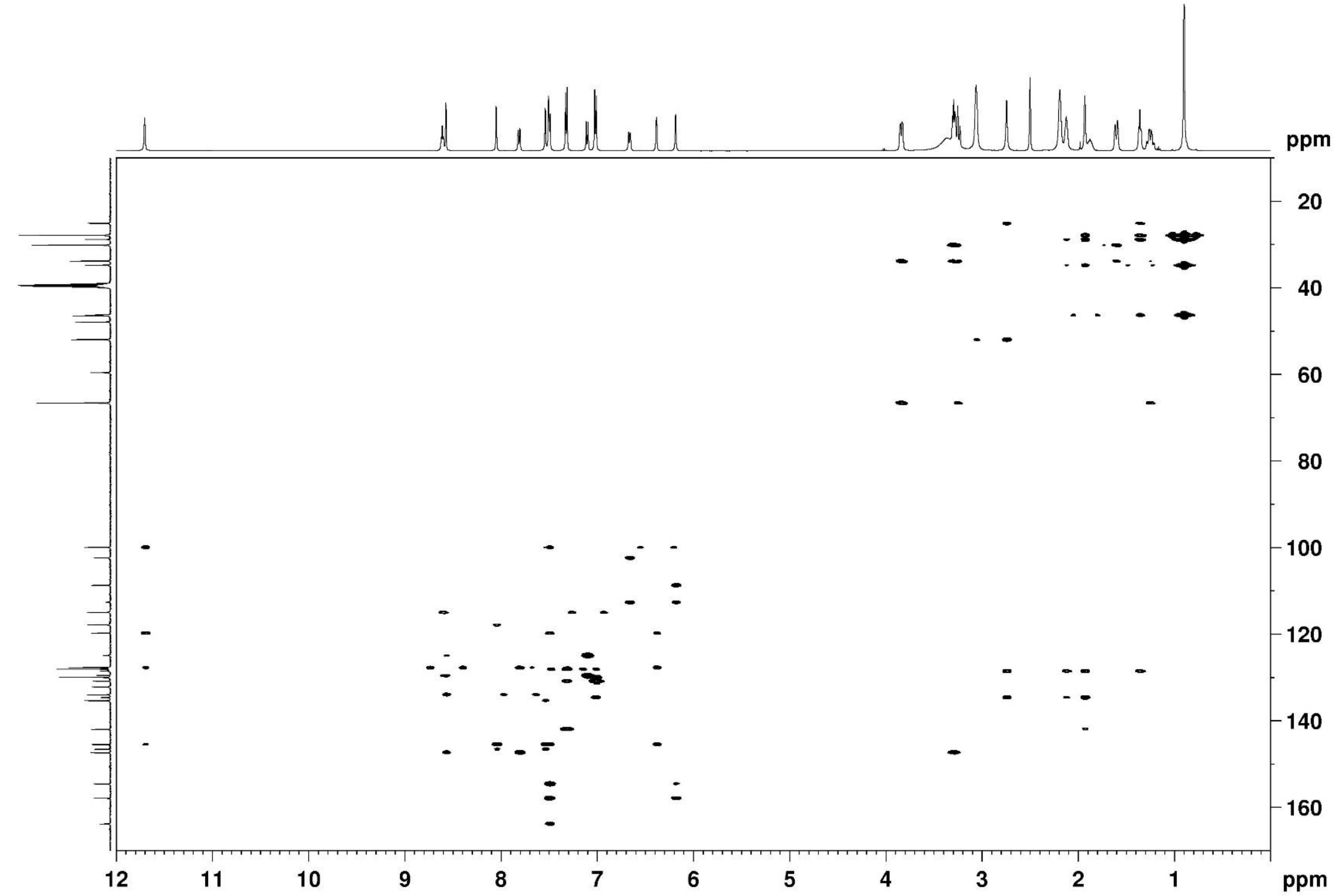

Figure S8 – (^{1}H, ^{13}C)-HMBC spectrum of **venetoclax**

Degradation product A1 information

3-nitro-4-(((tetrahydro-2*H*-pyran-4-yl)methyl)amino)benzenesulfonamide: crystalline solid; m.p. 190.3 °C; HRMS $[M+H]^+$: calculated 316.0962, found 316.0958.

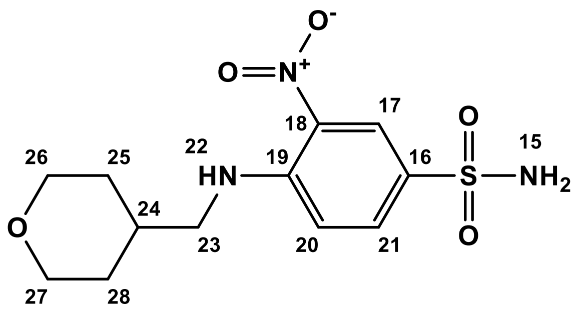

Figure S9 – Degradation product A1 with NMR assignations

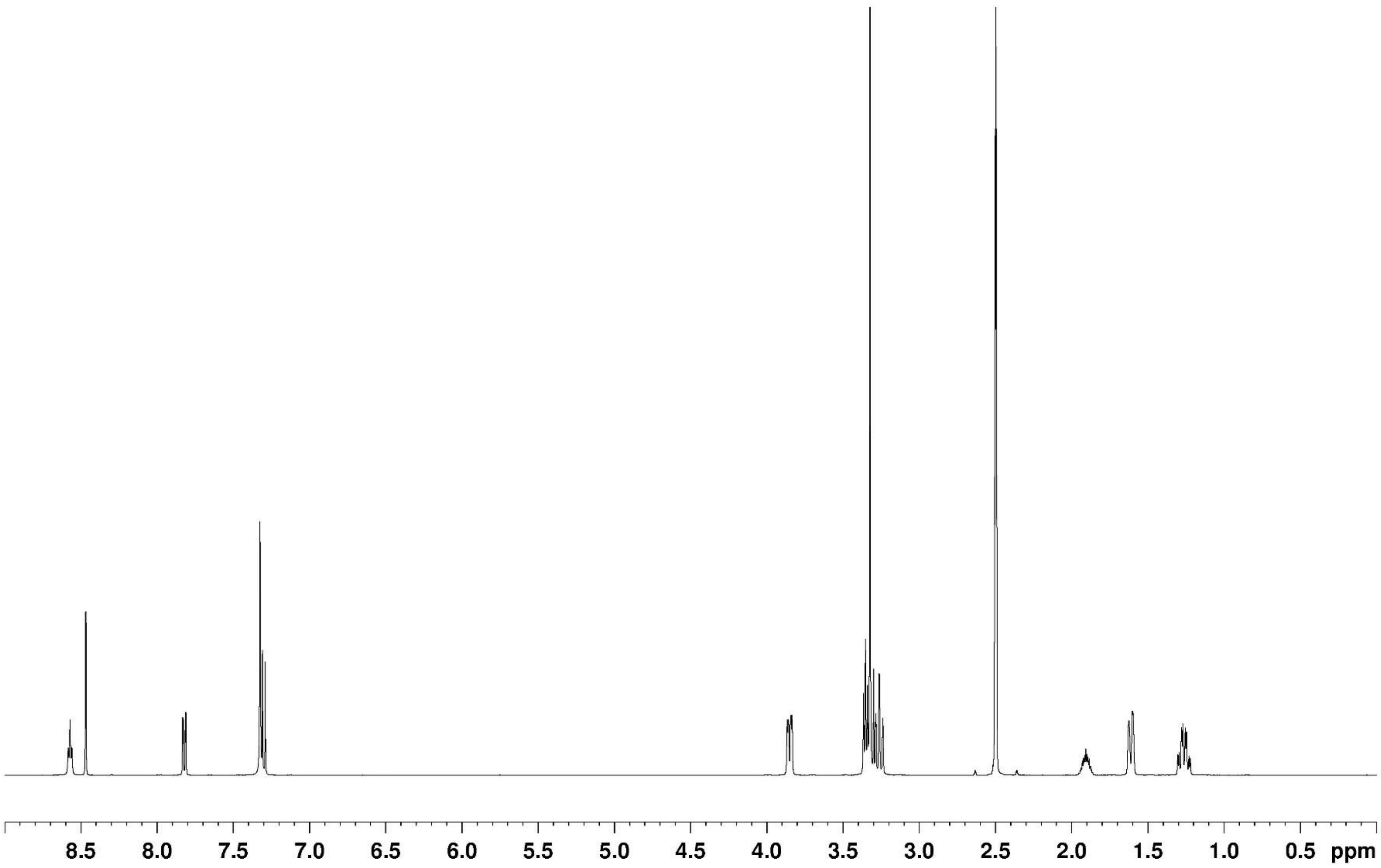

Figure S10 – ^{1}H NMR spectrum of **A1**

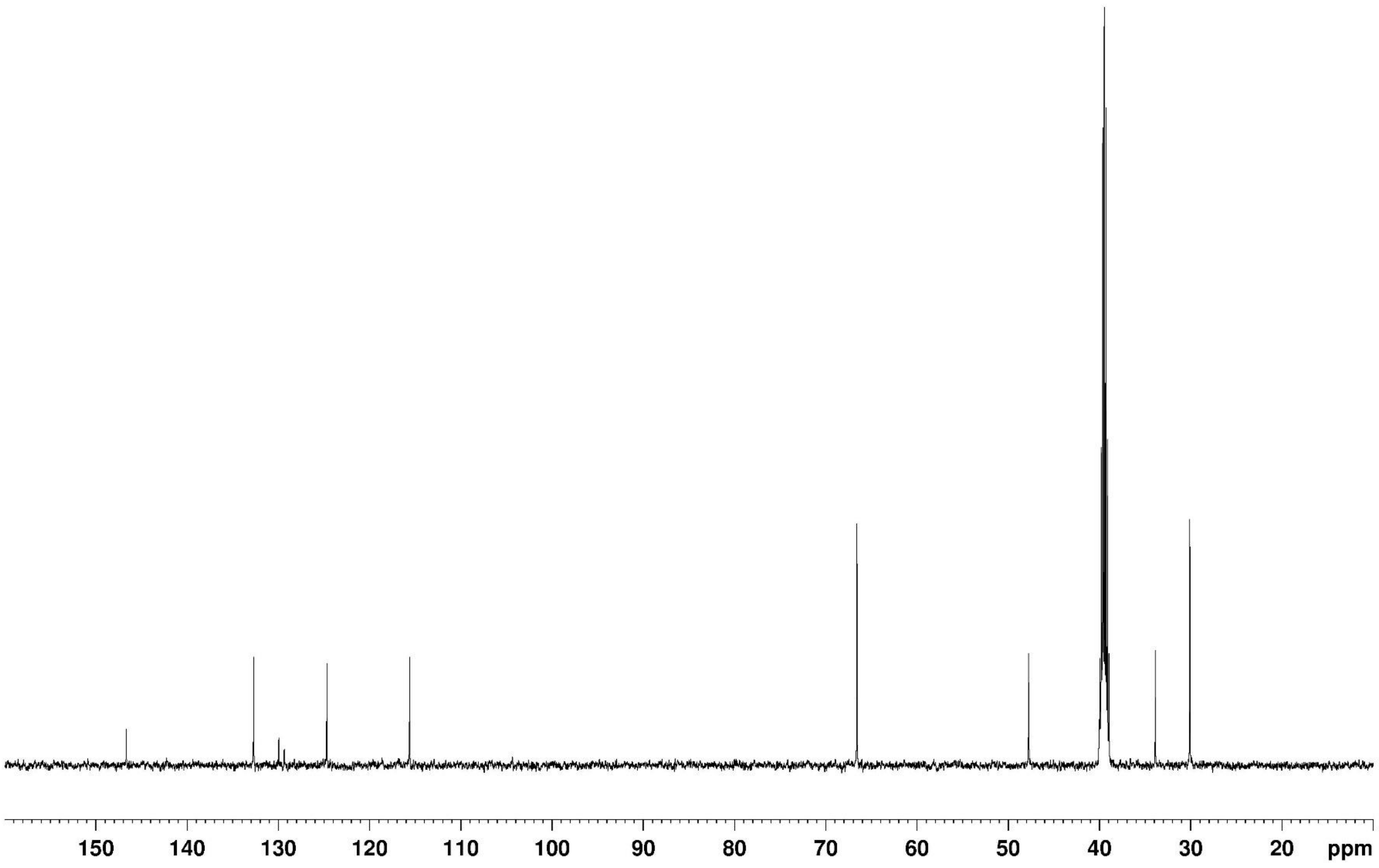

Figure S11 – ^{13}C NMR spectrum of **A1**

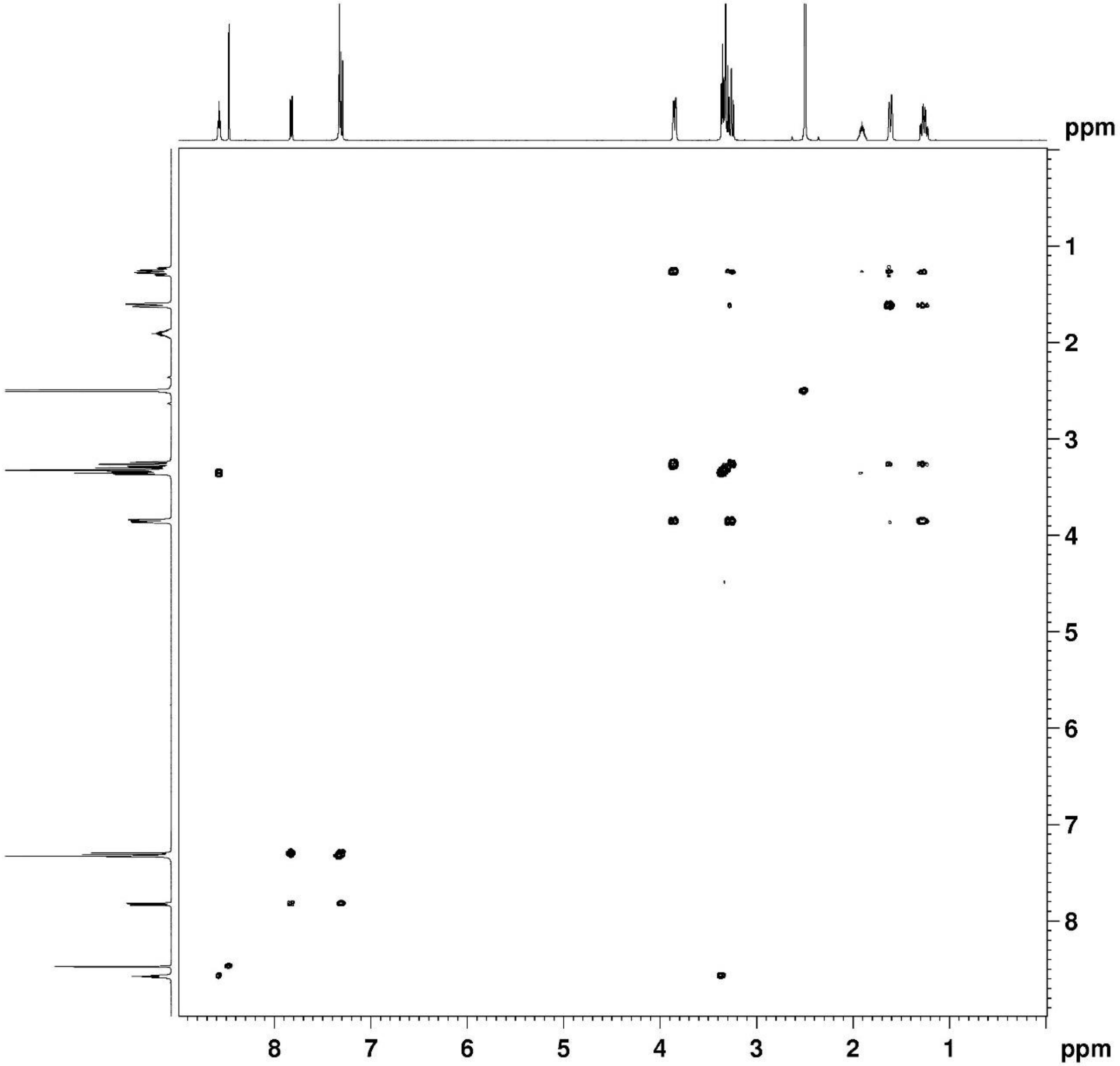

Figure S12 – (^{1}H, ^{1}H)-COSY spectrum of **A1**

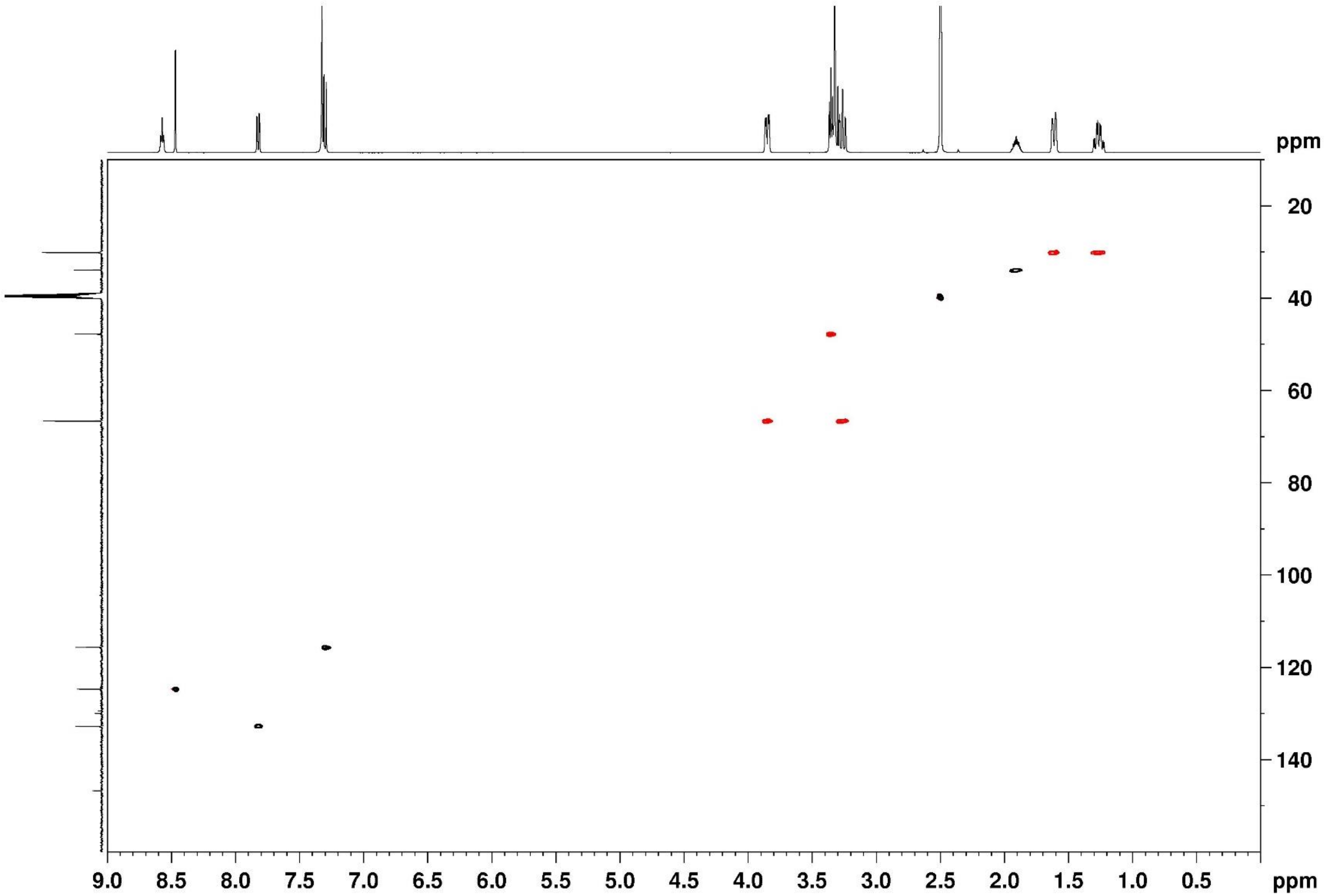

Figure S13 – (^{1}H, ^{13}C)-HSQC spectrum of **A1**

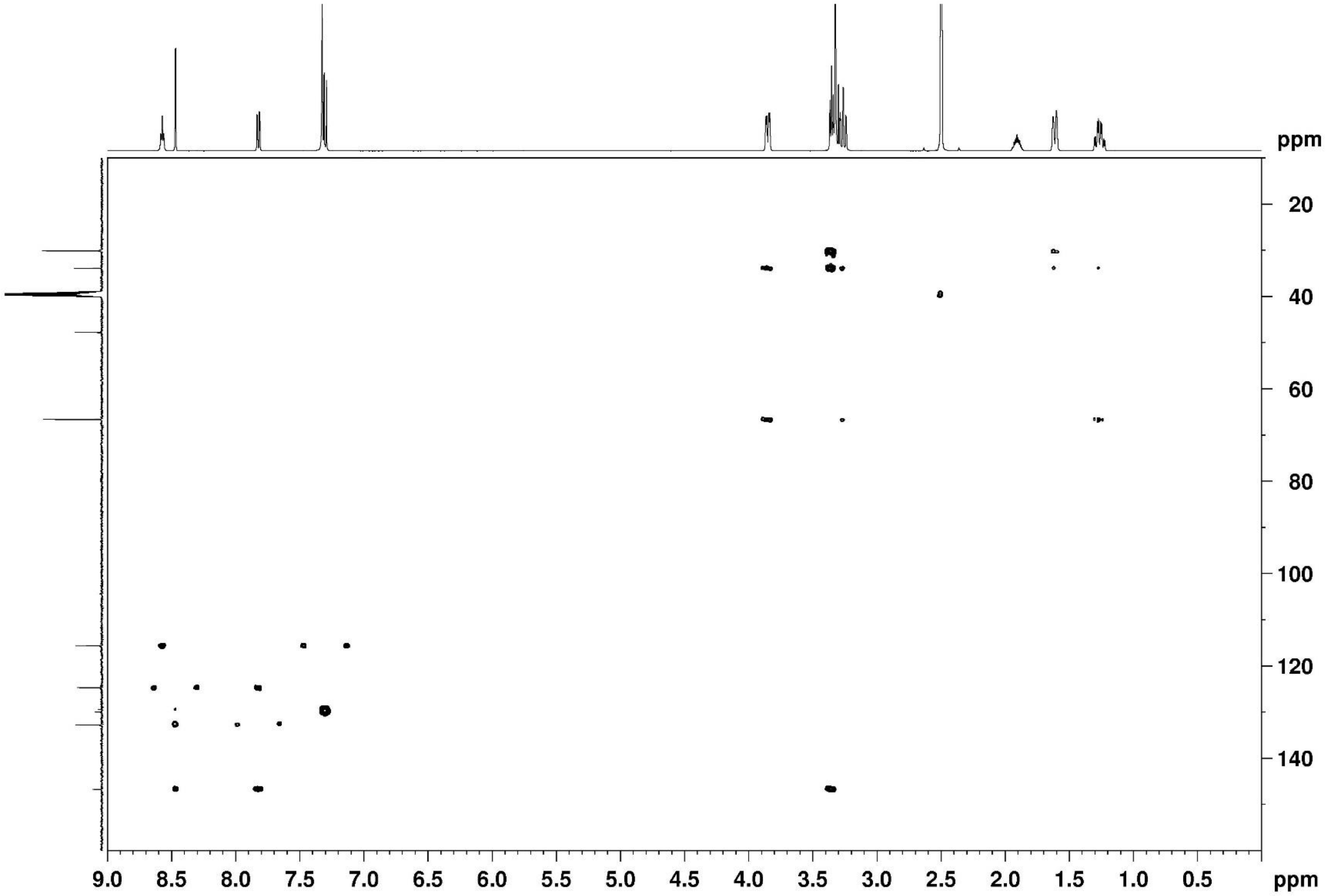

Figure S14 – (^{1}H, ^{13}C)-HMBC spectrum of **A1**

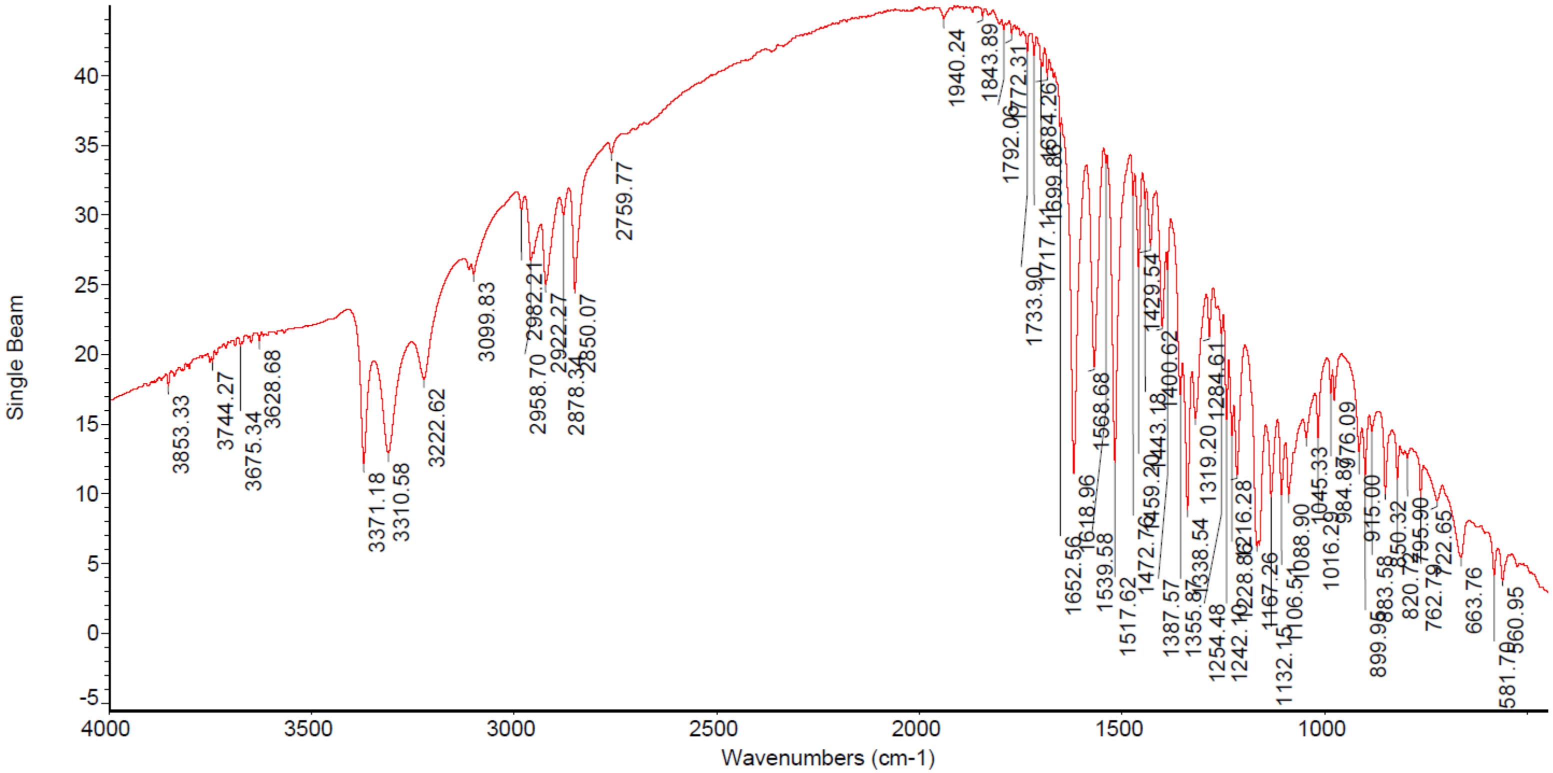

Figure S15 – IR spectrum of **A1**

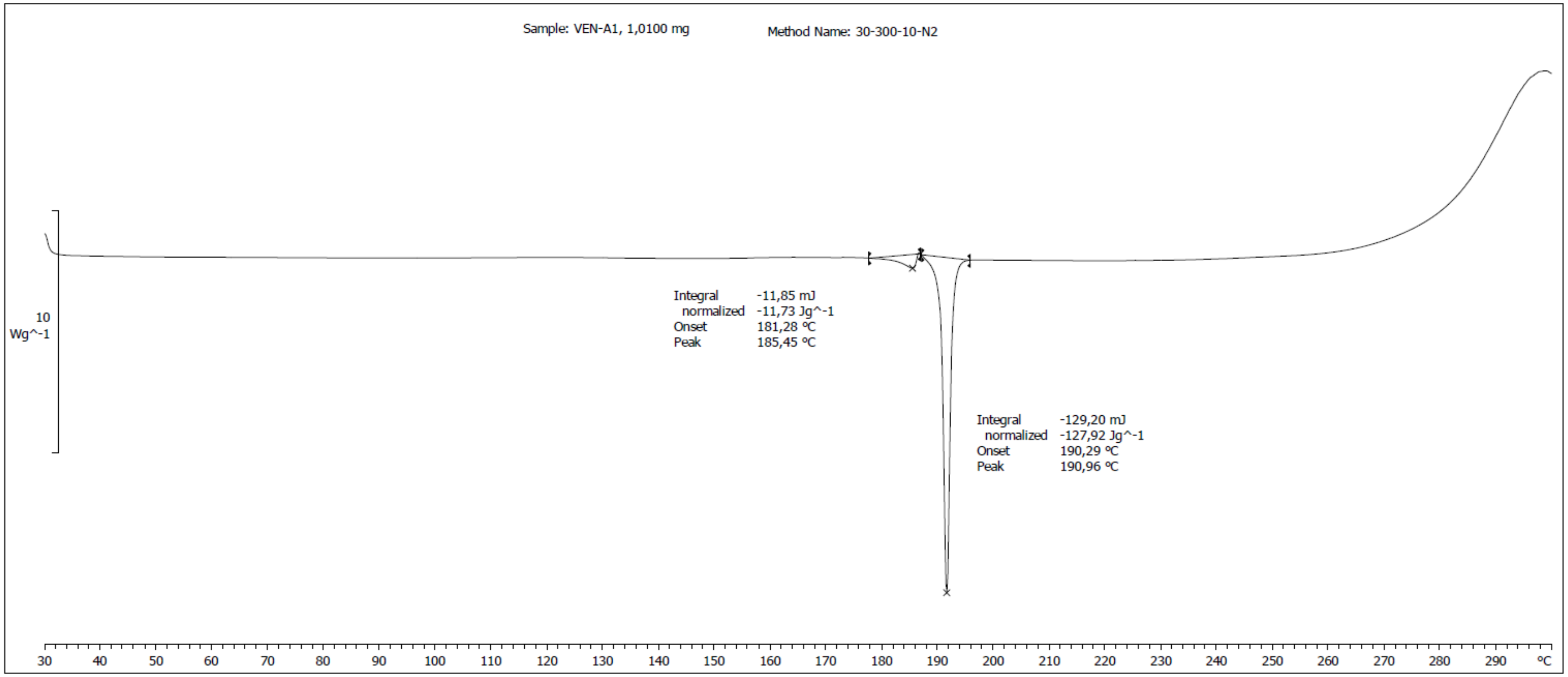

Figure S16 – DSC curve for **A1**

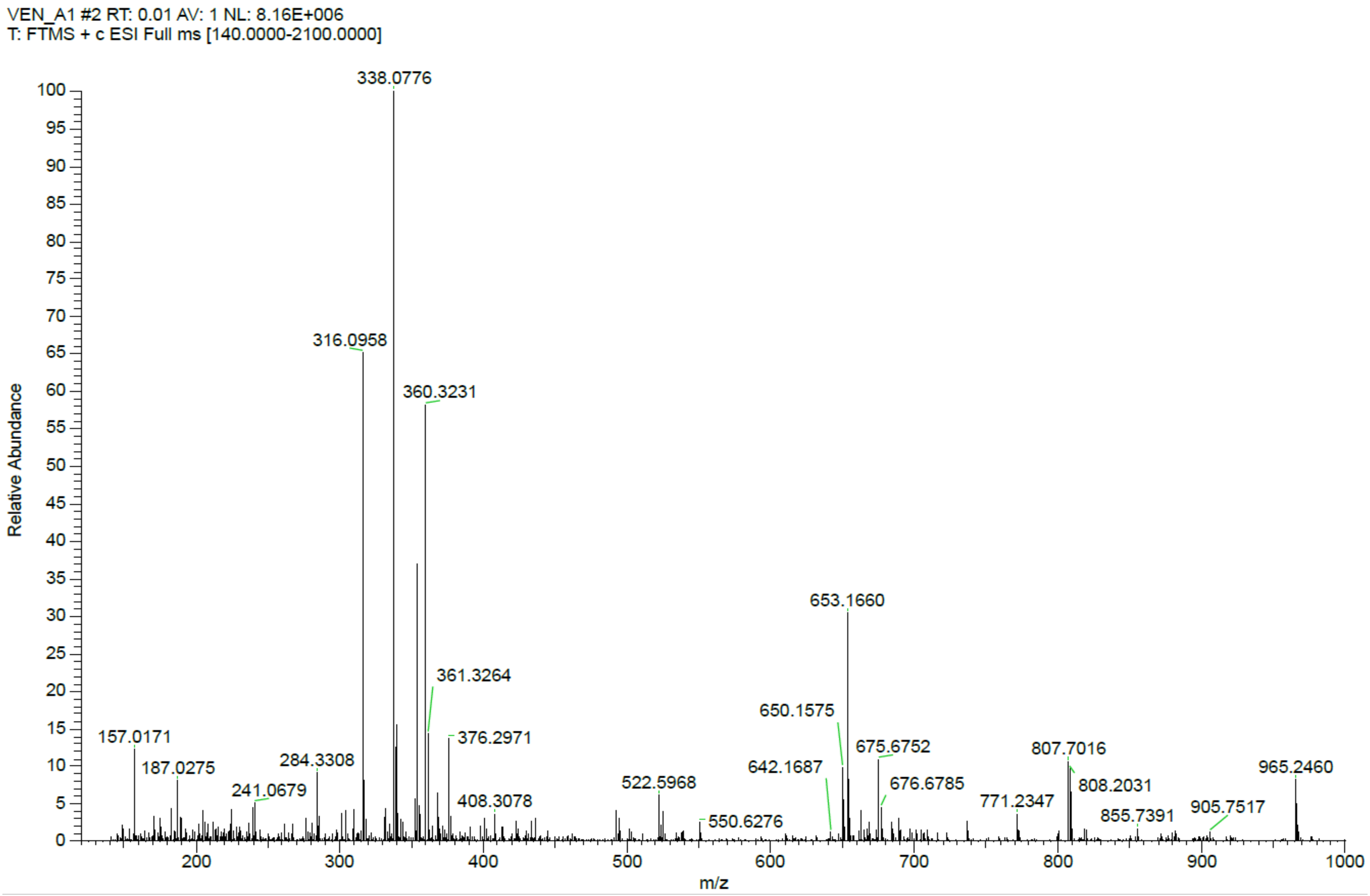

Figure S17 – HRMS spectrum of **A1**

Degradation product A2 information

2-((1*H*-pyrrolo[2,3-b]pyridin-5-yl)oxy)-4-(4-((4'-chloro-5,5-dimethyl-3,4,5,6-tetrahydro-[1,1'-biphenyl]-2-yl)methyl)piperazin-1-yl)benzoic acid: amorphous solid; HRMS $[M+H]^+$: calculated 571.2470, found 571.2458.

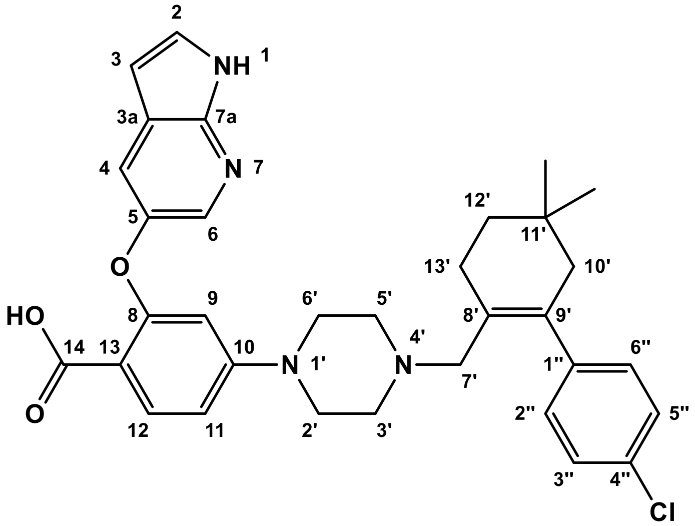

Figure S18 – Degradation product A2 with NMR assignations

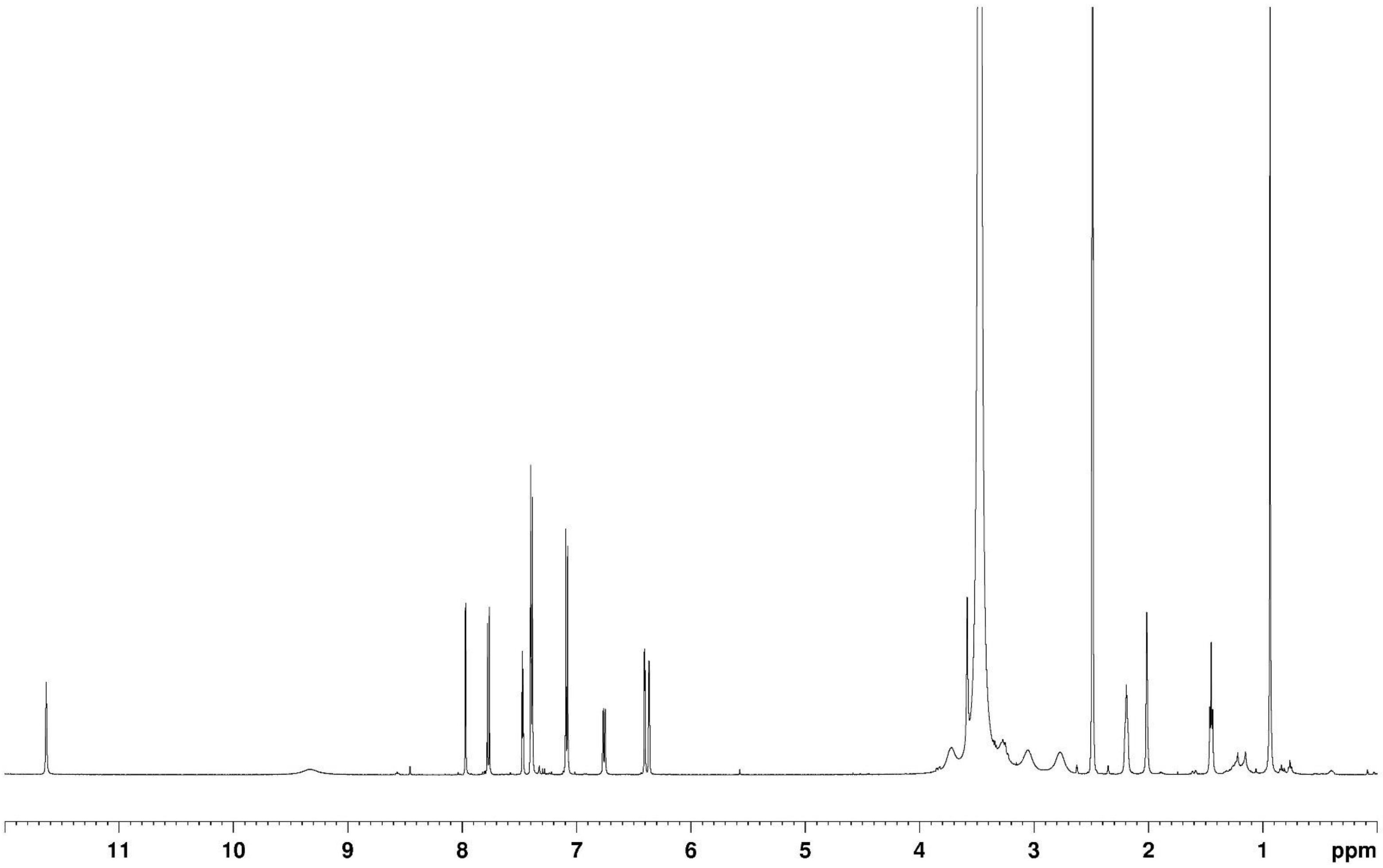

Figure S19 – ^{1}H NMR spectrum of **A2**

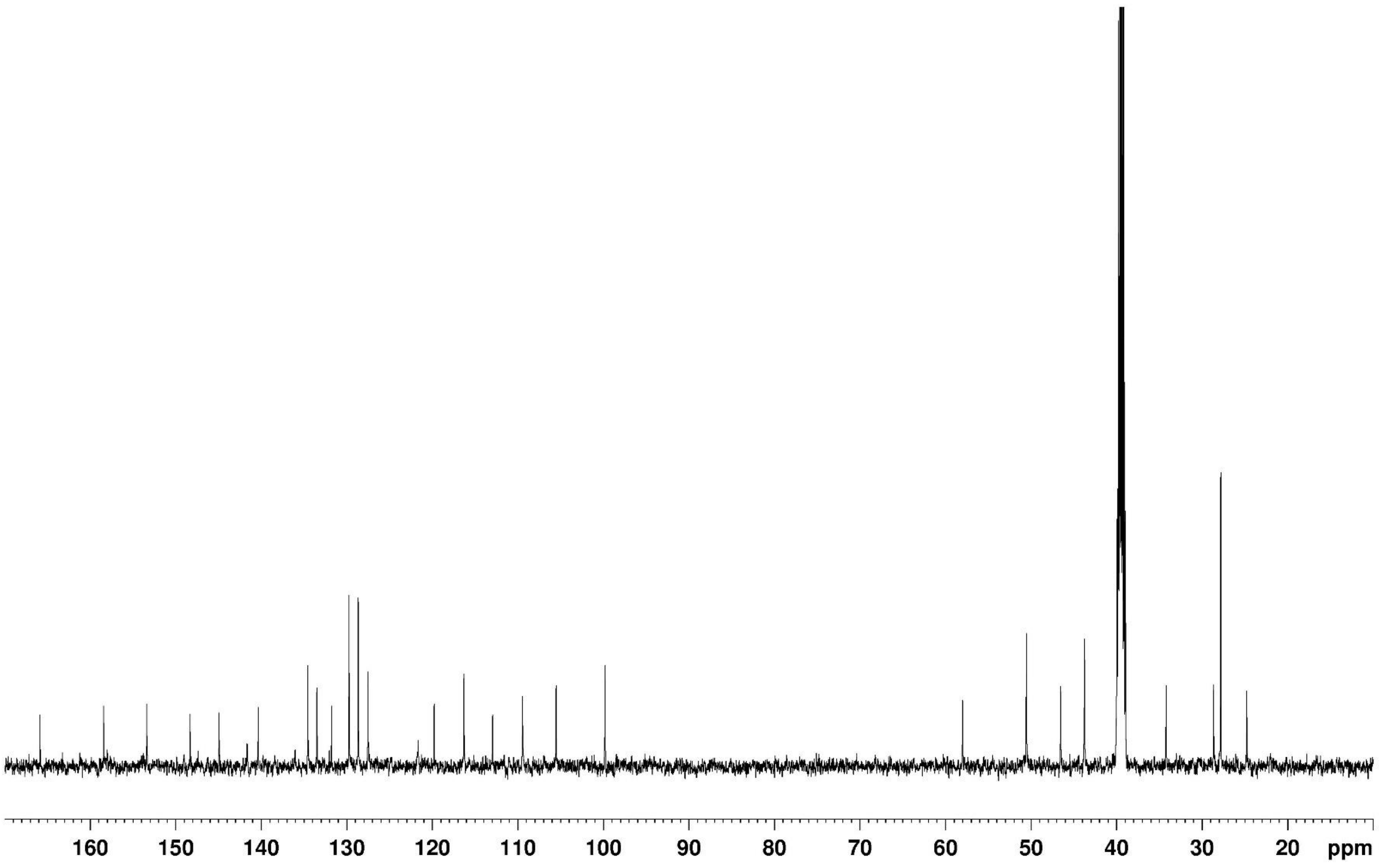

Figure S20 – ^{13}C NMR spectrum of **A2**

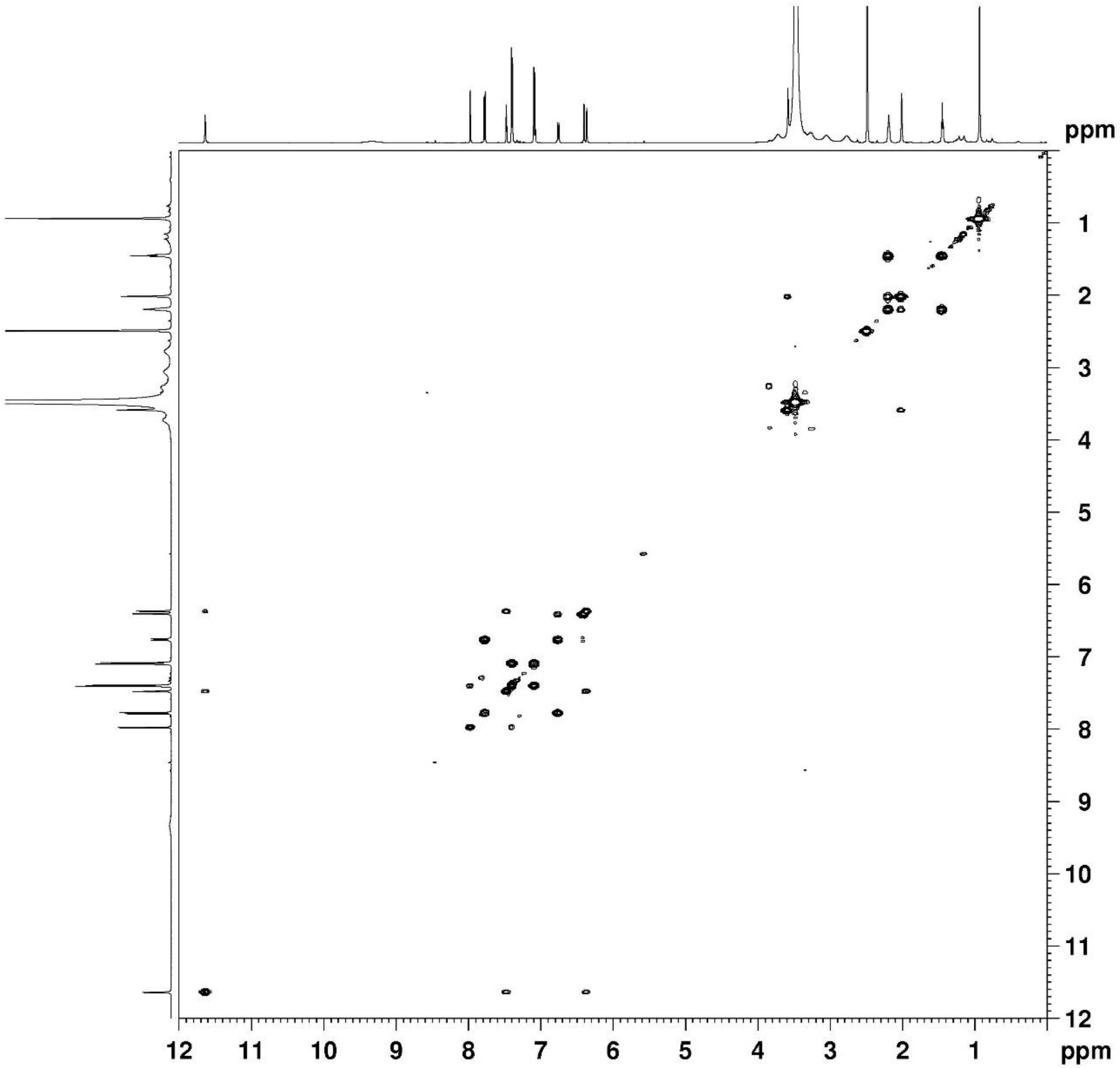

Figure S21 – (^{1}H, ^{1}H)-COSY spectrum of **A2**

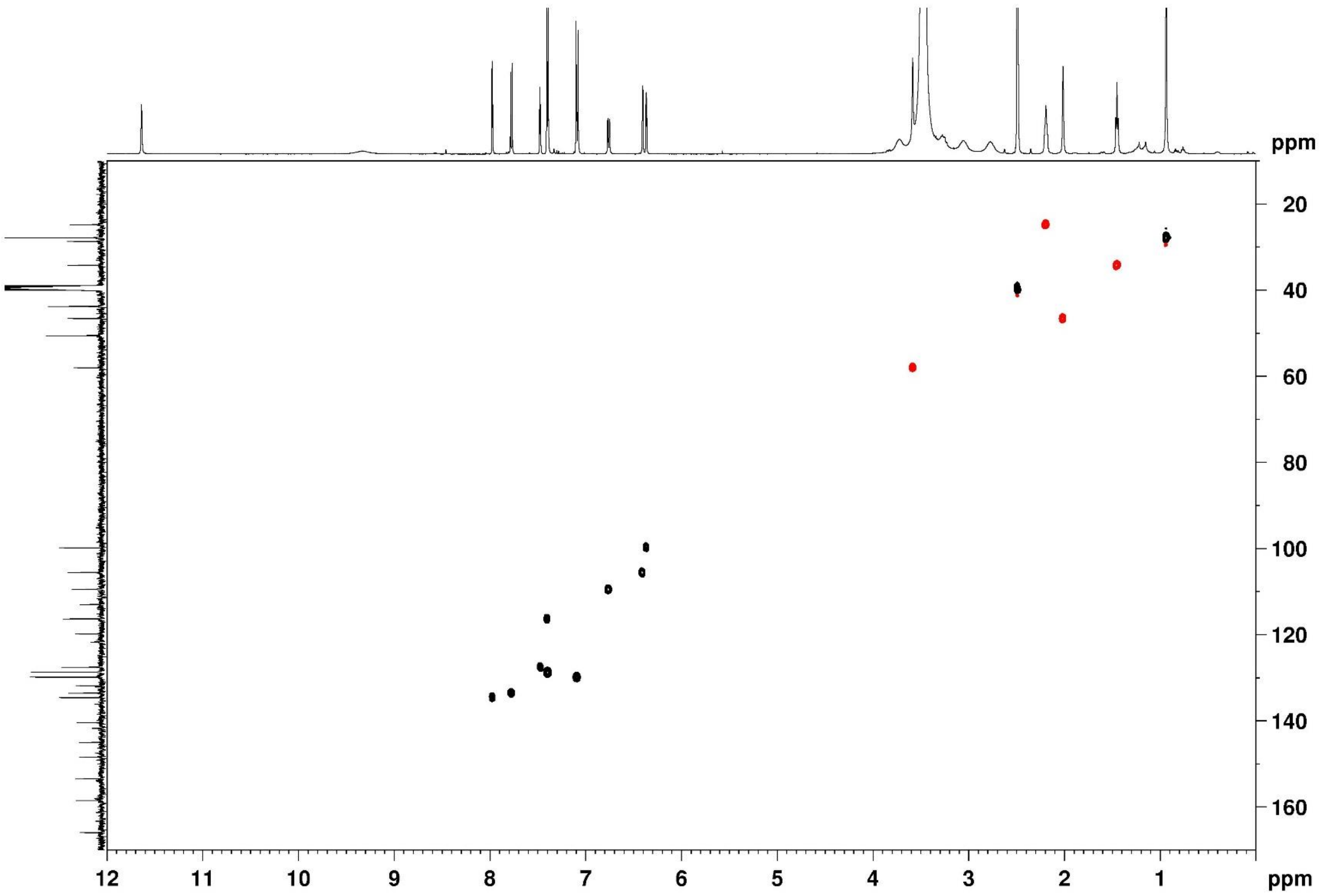

Figure S22 – (^{1}H, ^{13}C)-HSQC spectrum of **A2**

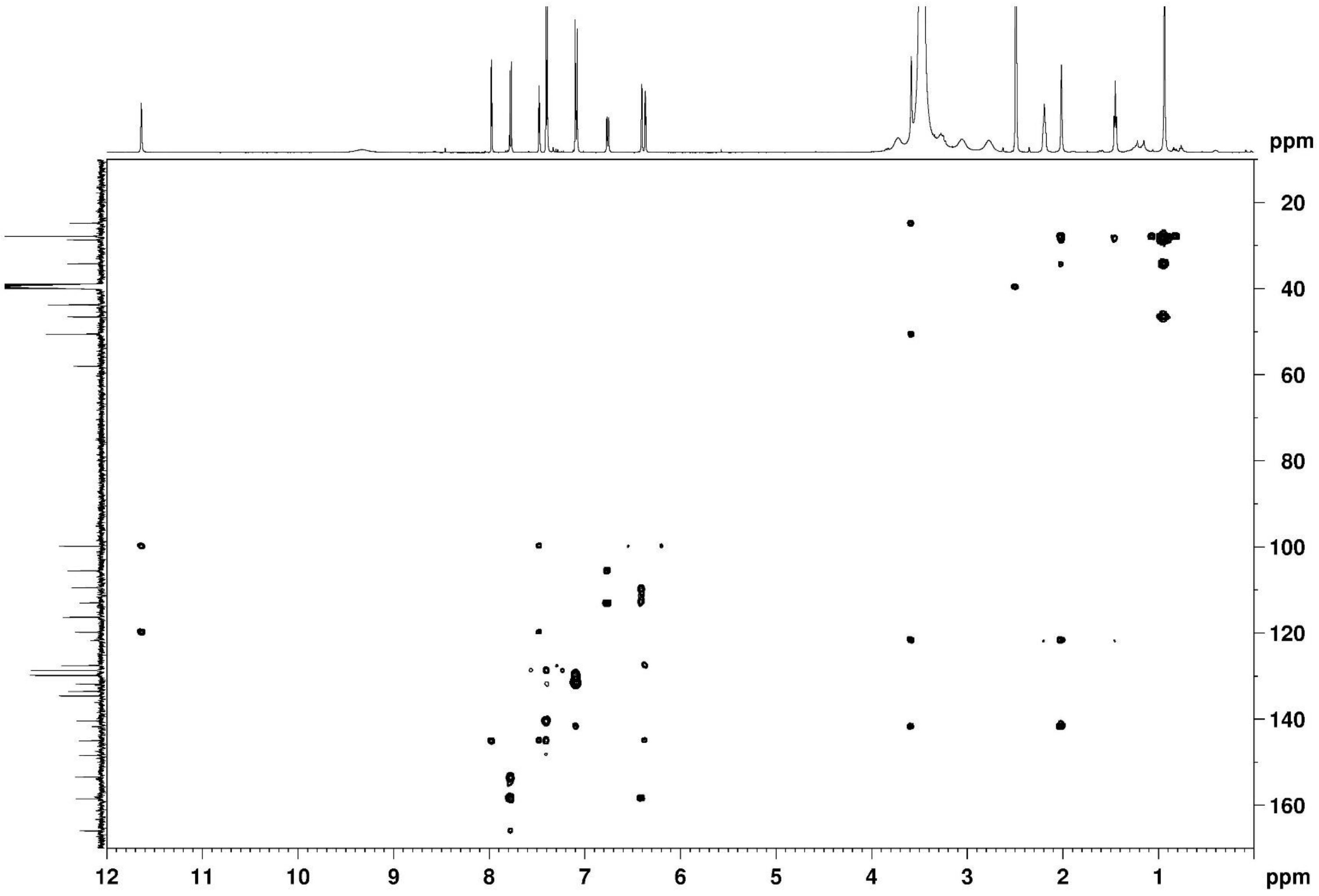

Figure S23 – (^{1}H, ^{13}C)-HMBC spectrum of **A2**

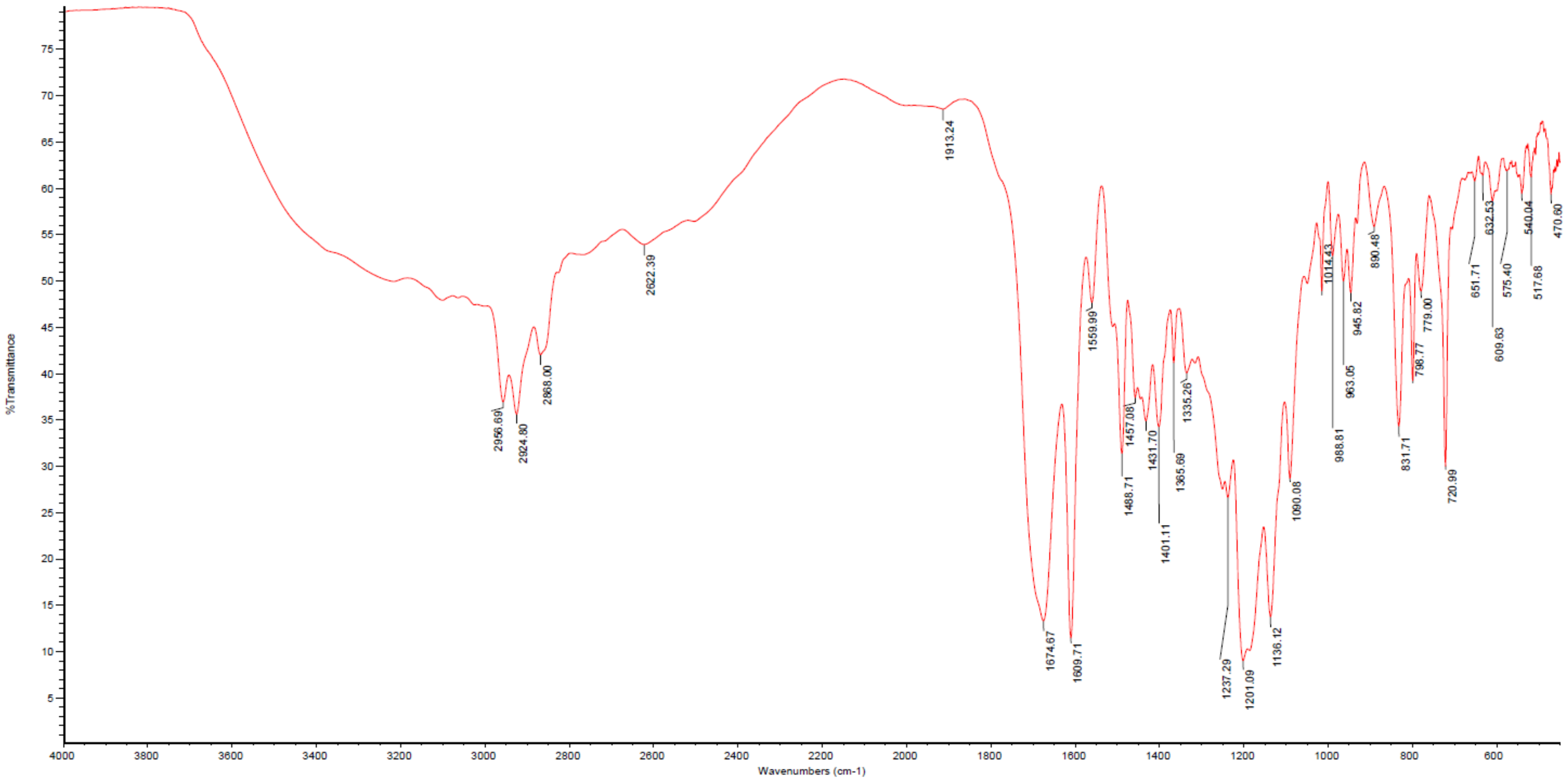

Figure S24 – IR spectrum of **A2**

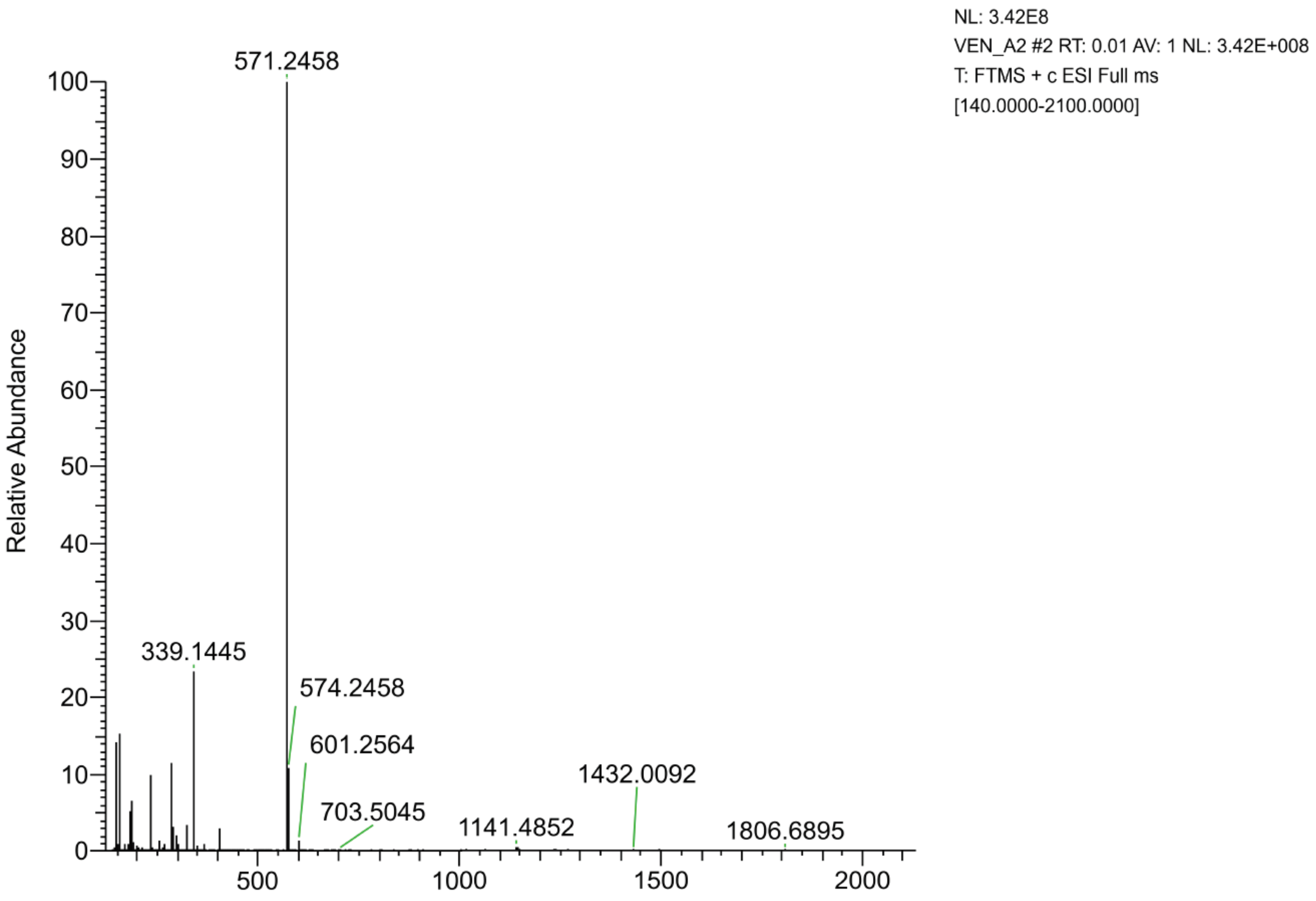

Figure S25 – HRMS spectrum of **A2**

Degradation product A3/B3 information

2,2'-((methylenebis(1*H*-pyrrolo[2,3-b]pyridine-1,5-diyl))bis(oxy))bis(4-(4-((4'-chloro-5,5-dimethyl-3,4,5,6-tetrahydro-[1,1'-biphenyl]-2-yl)methyl)piperazin-1-yl)-*N*-((3-nitro-4-(((tetrahydro-2*H*-pyran-4-yl)methyl)amino)phenyl)sulfonyl)benzamide): amorphous solid; HRMS $[M+H]^+$: calculated 1747.6435, found 1747.6434.

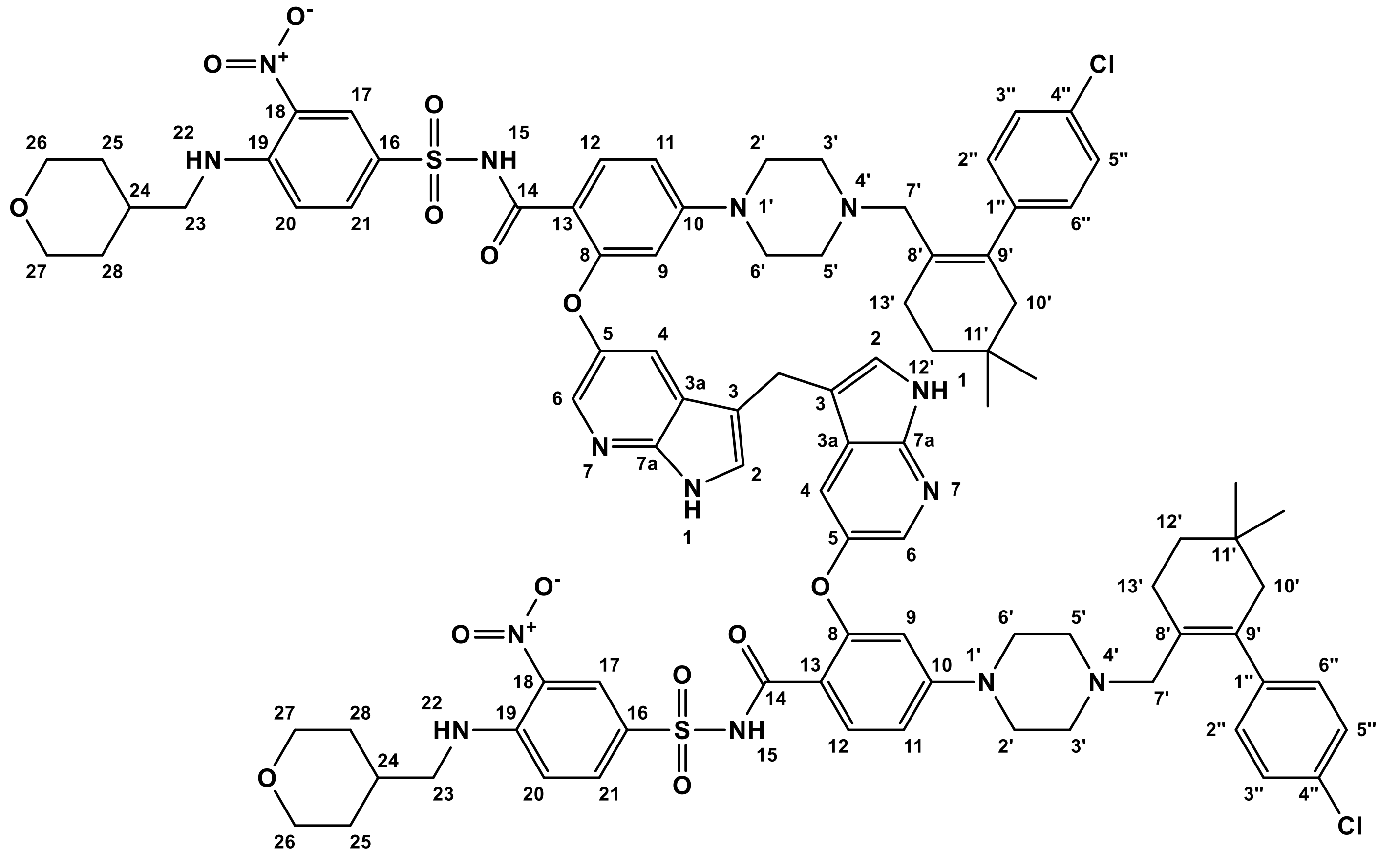

Figure S26 – Degradation product A3 with NMR assignations

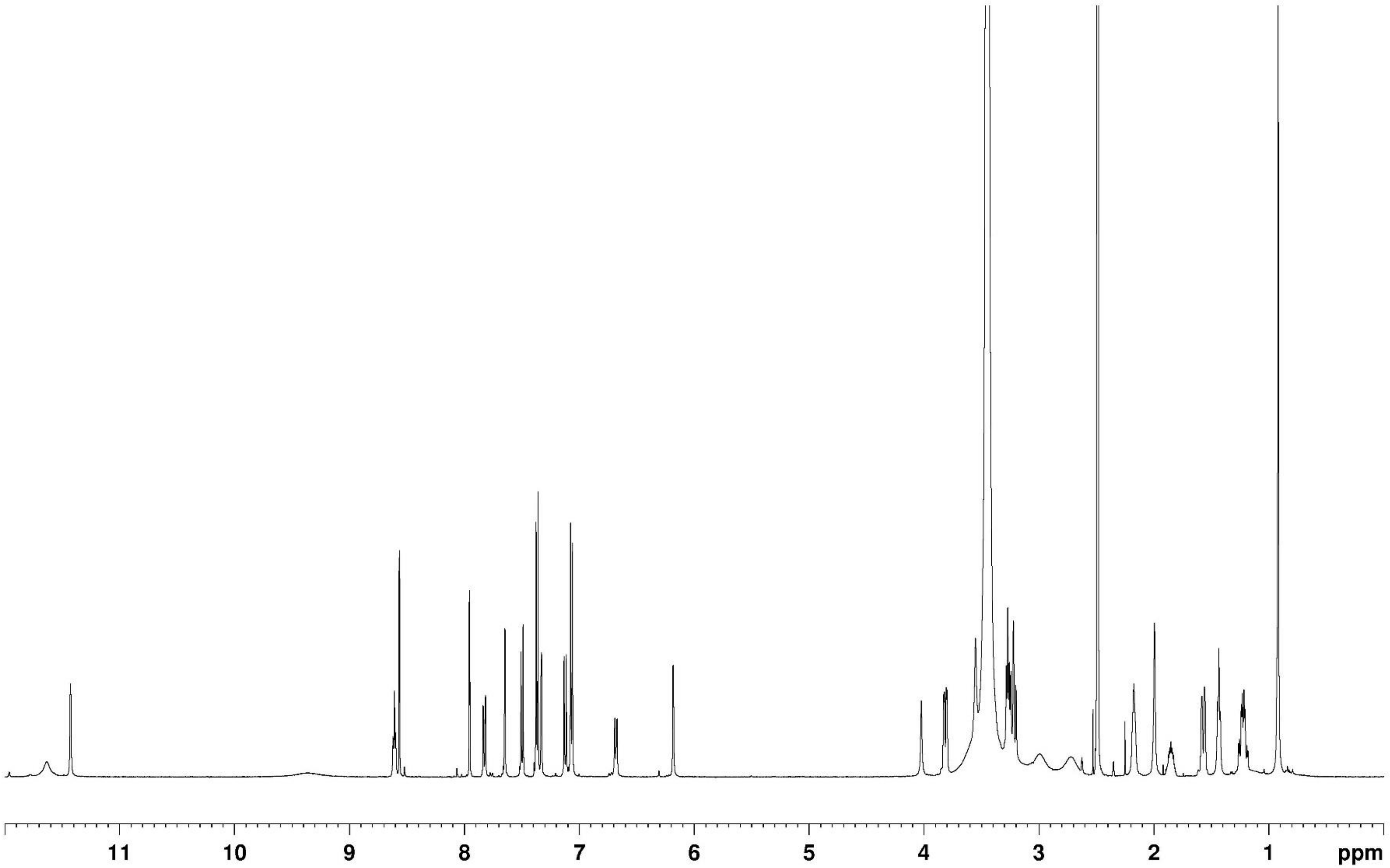

Figure S27 – ^{1}H NMR spectrum of **A3**

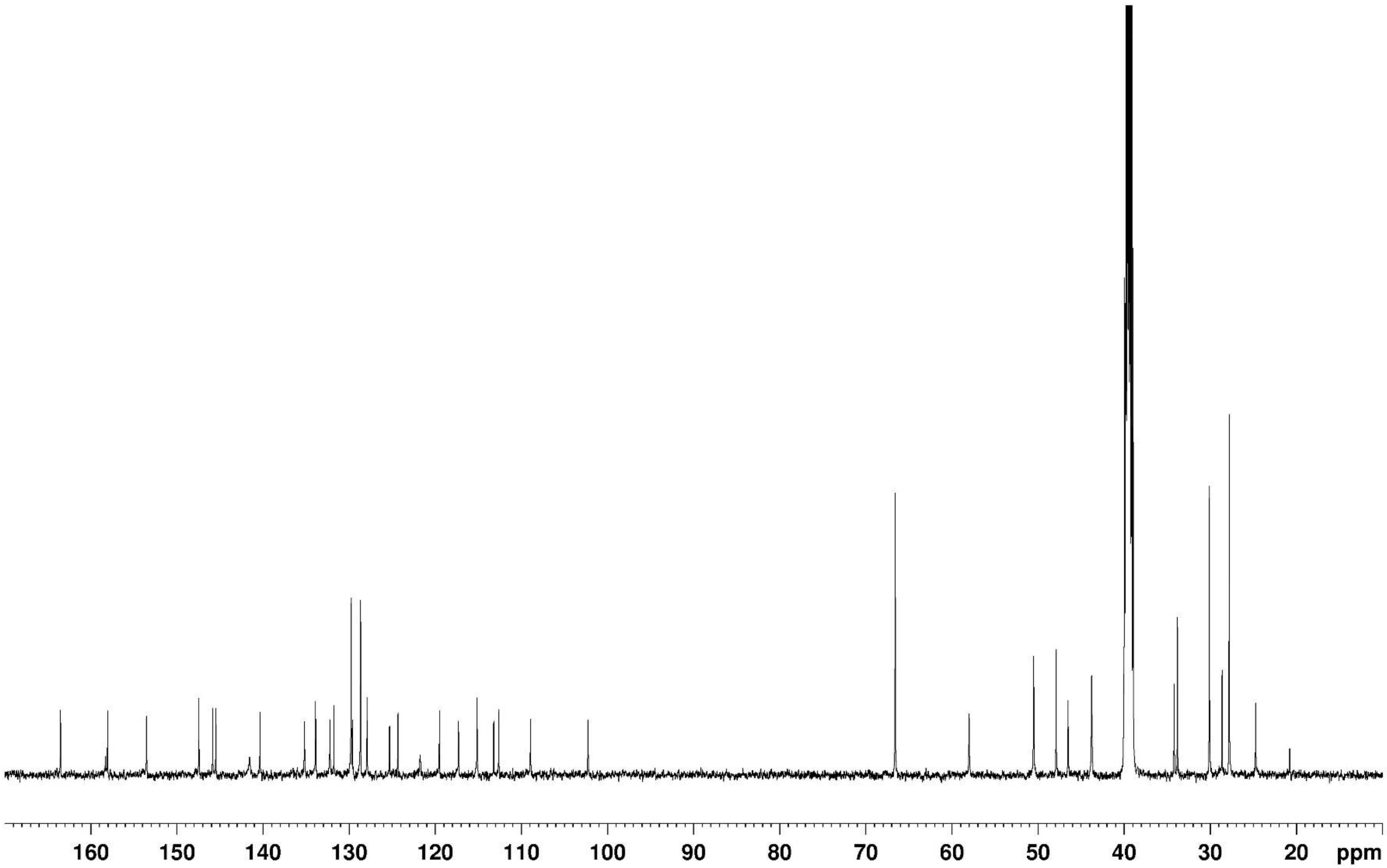

Figure S28 – ^{13}C NMR spectrum of **A3**

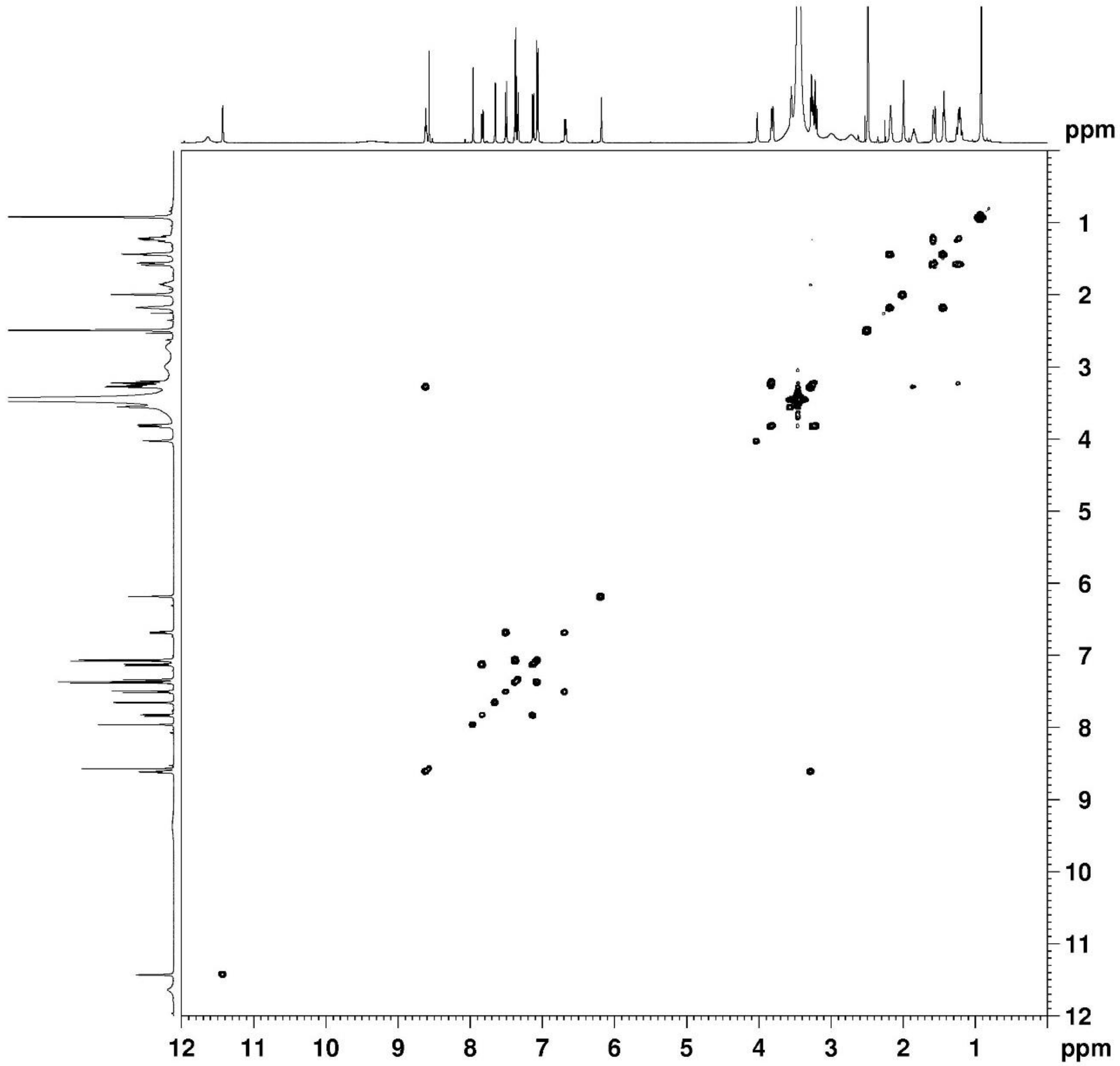

Figure S29 – (^{1}H, ^{1}H)-COSY spectrum of **A3**

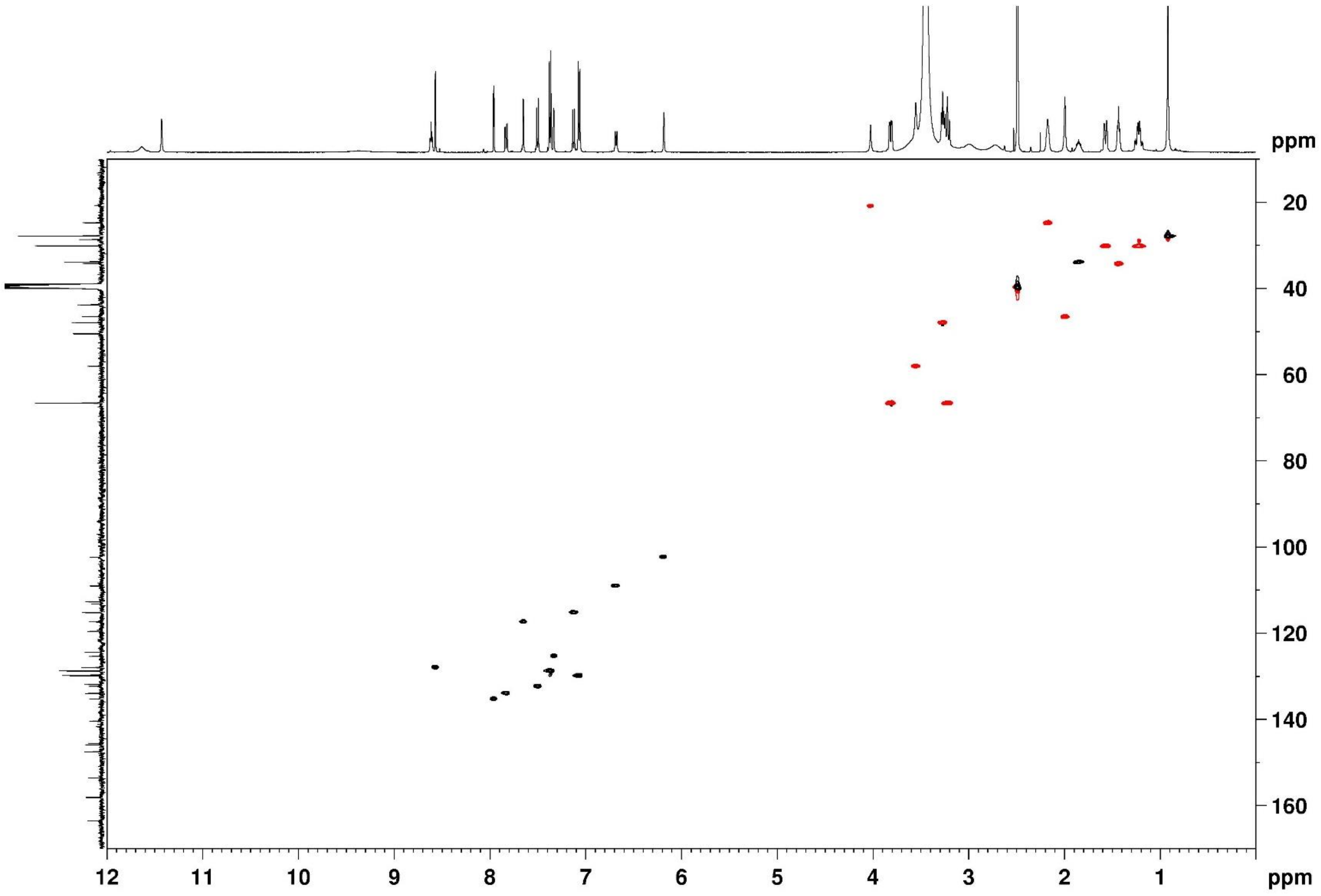

Figure S30 – (^{1}H, ^{13}C)-HSQC spectrum of **A3**

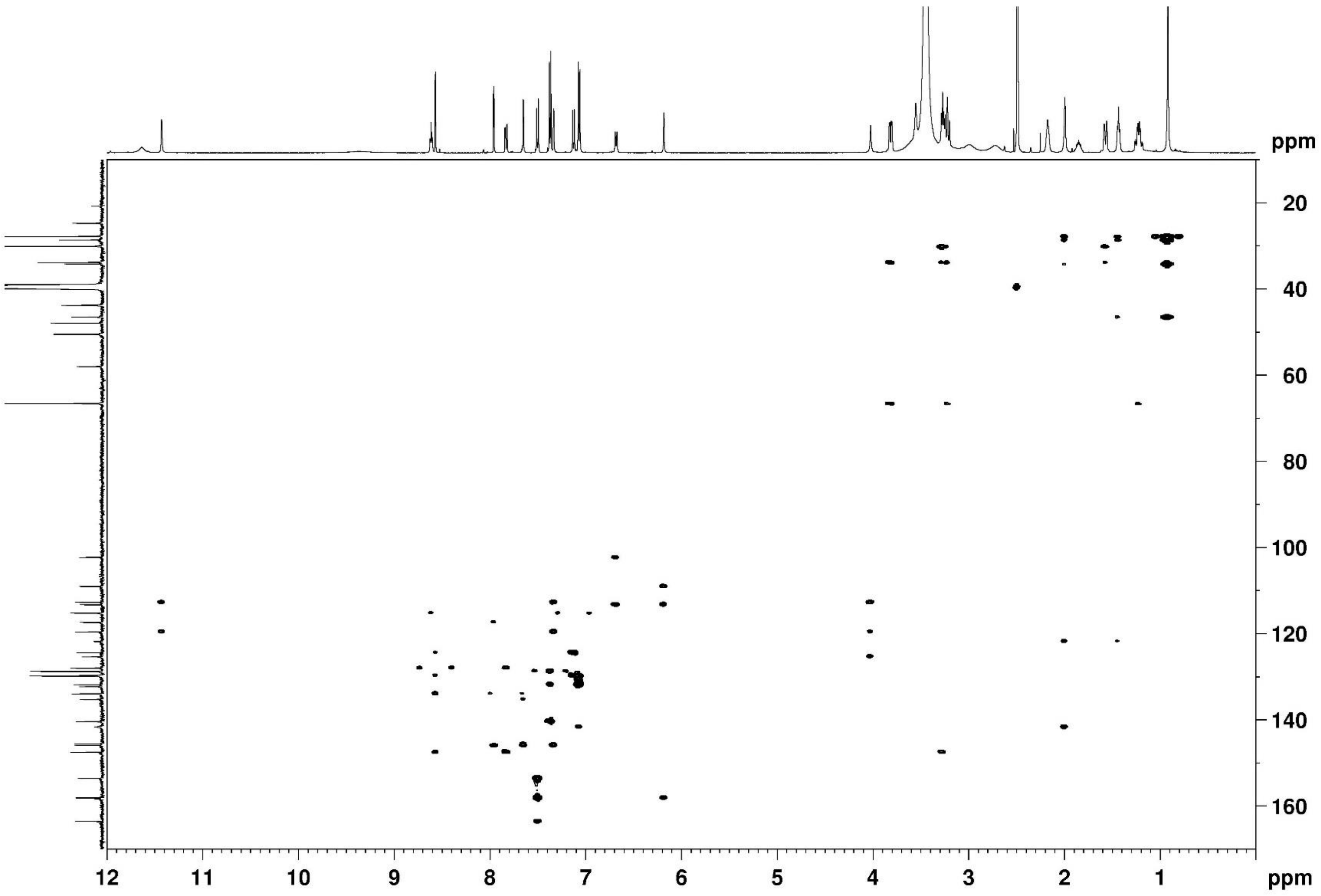

Figure S31 – (^{1}H, ^{13}C)-HMBC spectrum of **A3**

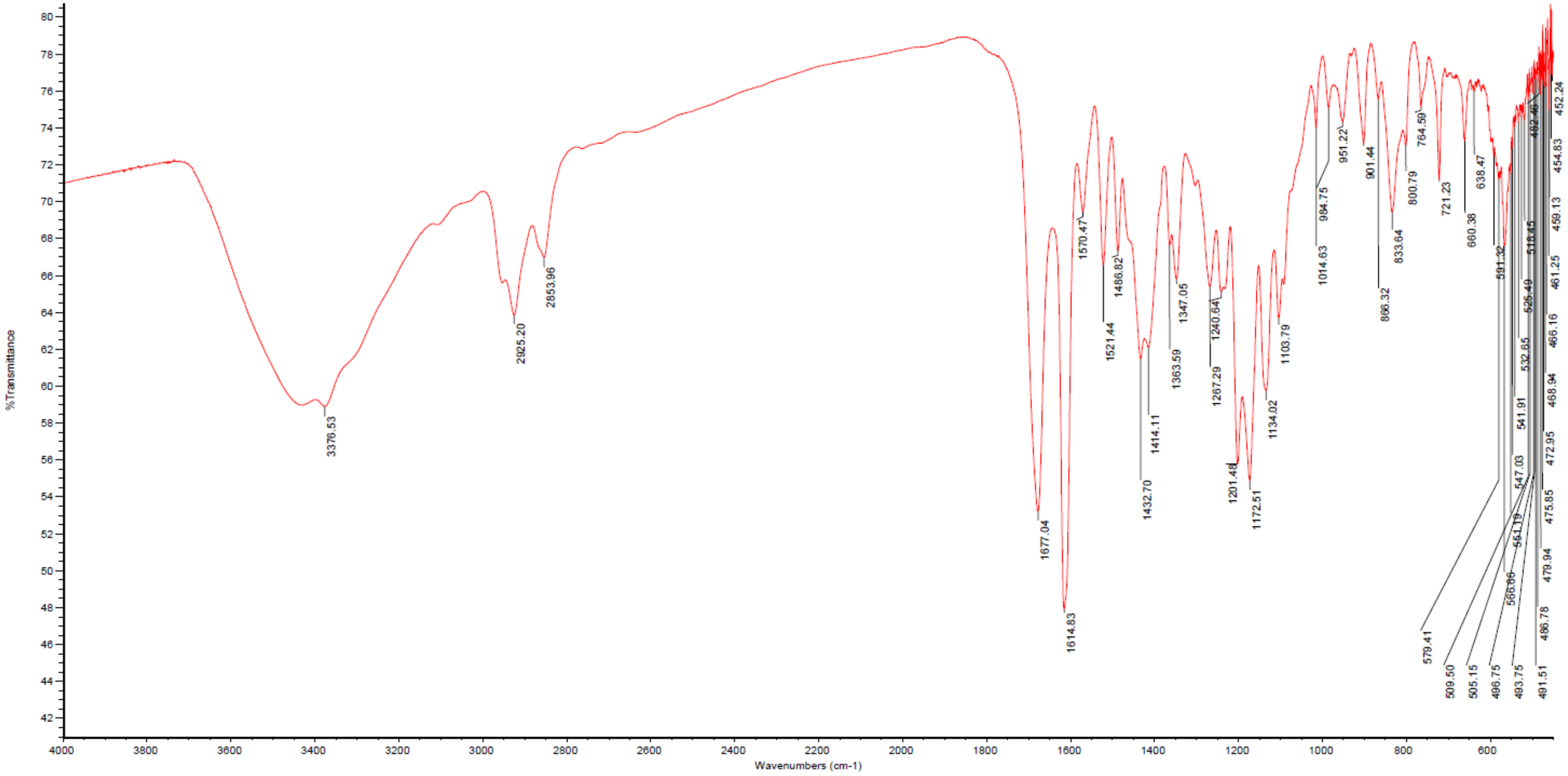

Figure S32 – IR spectrum of **A3**

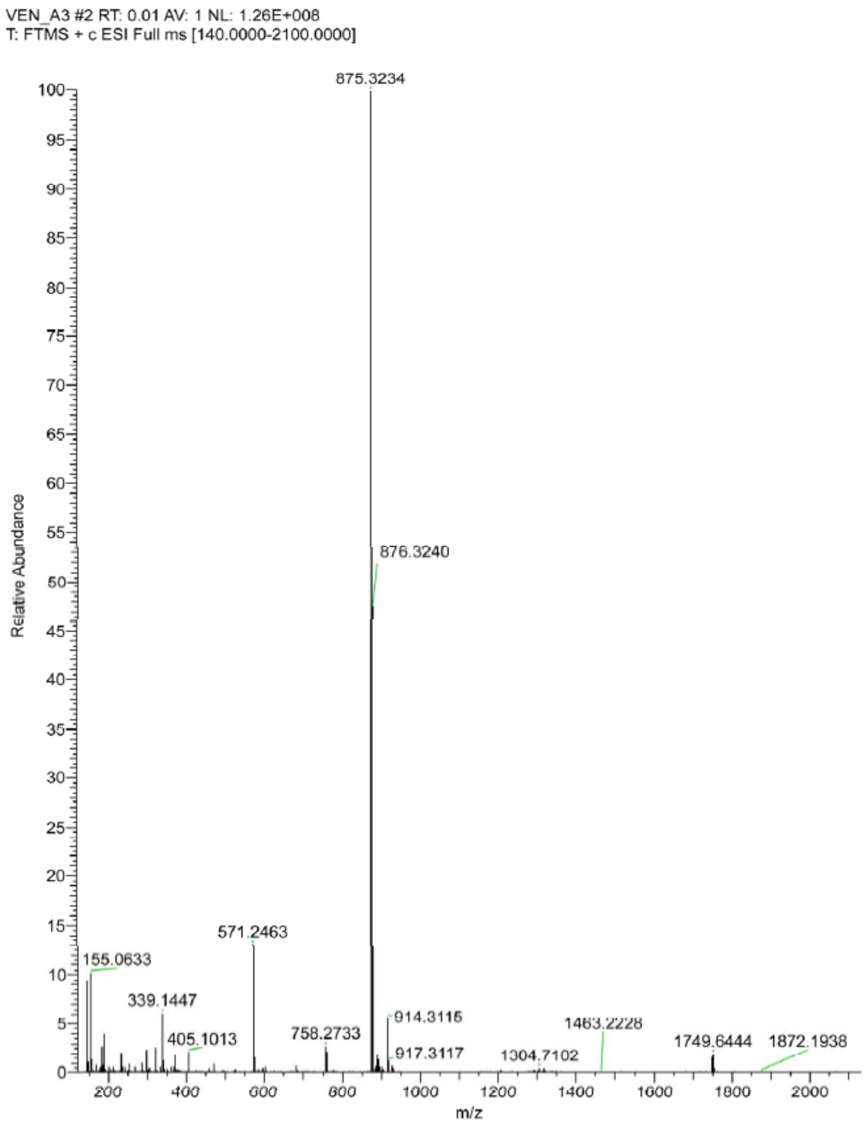

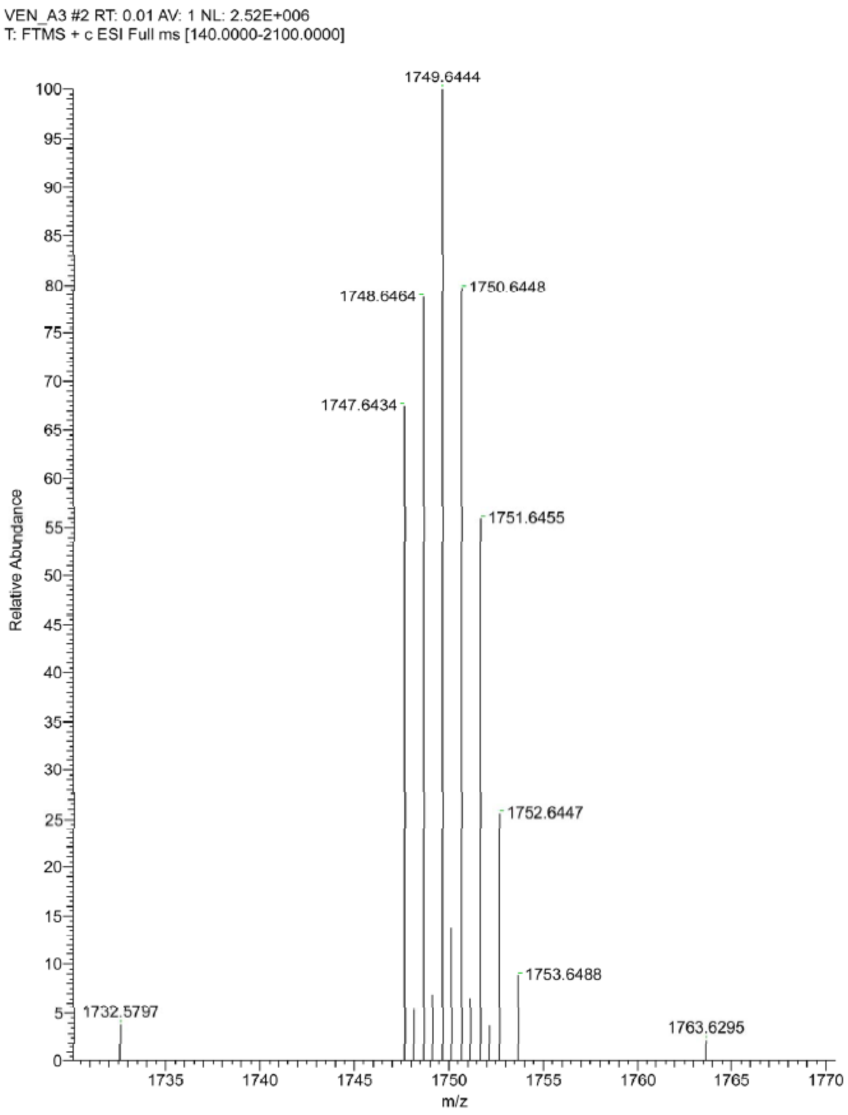

Figure S33 – HRMS spectrum of **A3**. Full spectrum (left) and spectrum of higher m/z (right)

Degradation product A4 information

5-(3-(4-((4'-chloro-5,5-dimethyl-3,4,5,6-tetrahydro-[1,1'-biphenyl]-2-yl)methyl)piperazin-1-yl)phenoxy)-1*H*-pyrrolo[2,3-b]pyridine: HRMS [M+H]+: calculated 527.2572, found 527.2543.

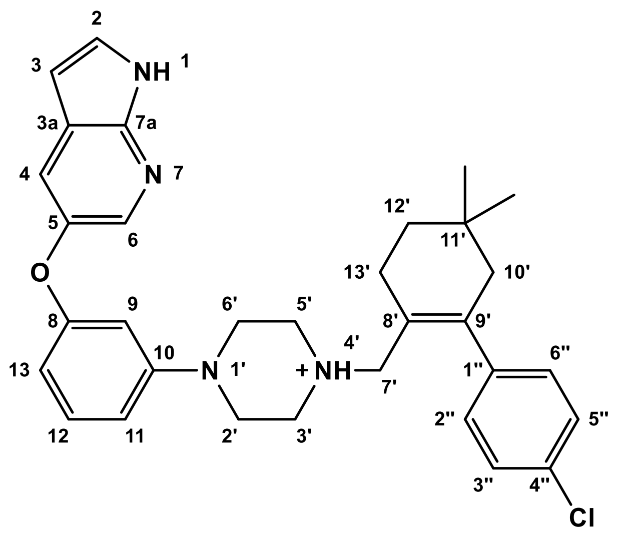

Figure S34 – Degradation product A4 with NMR assignations

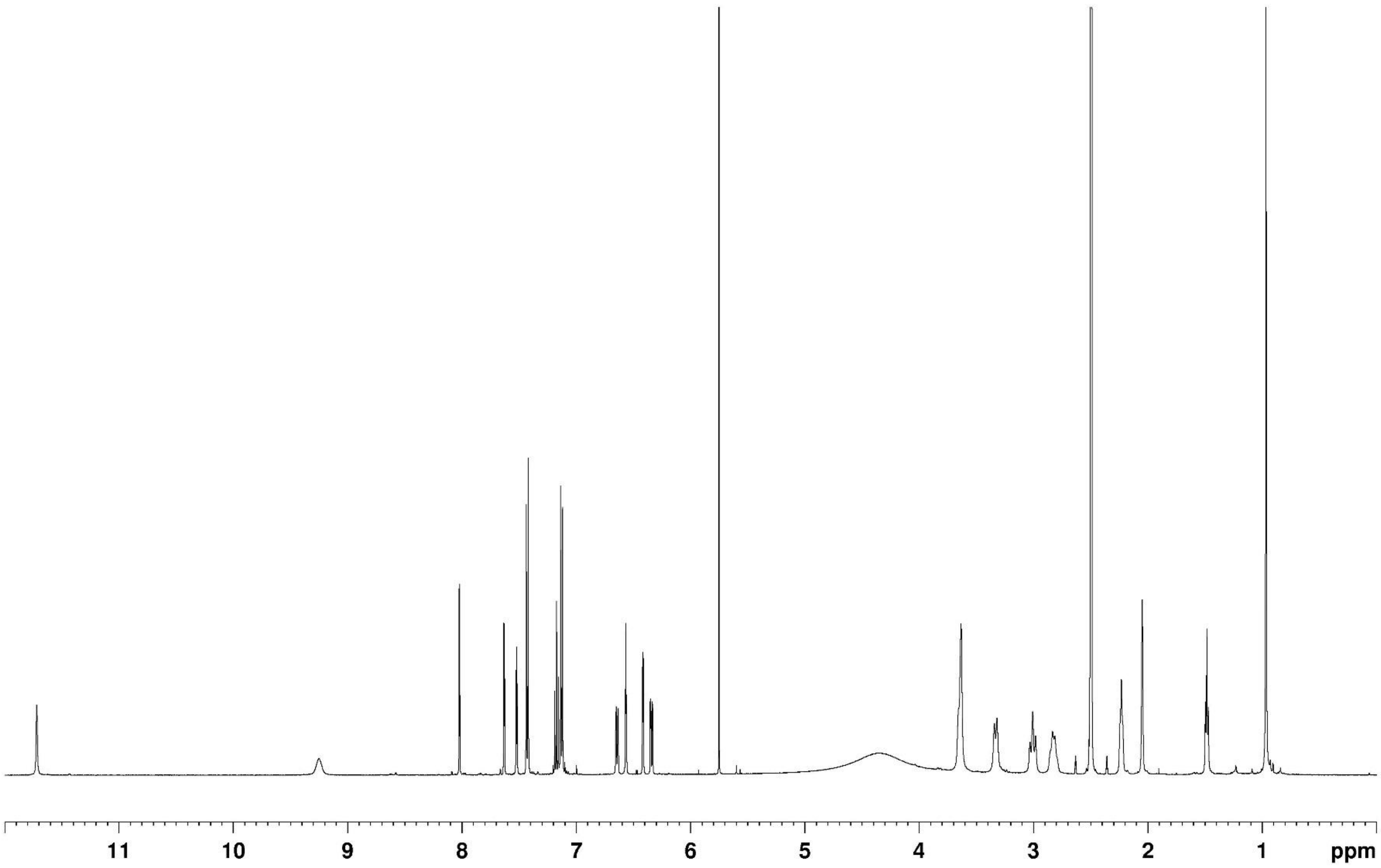

Figure S35 – ^{1}H NMR spectrum of **A4**

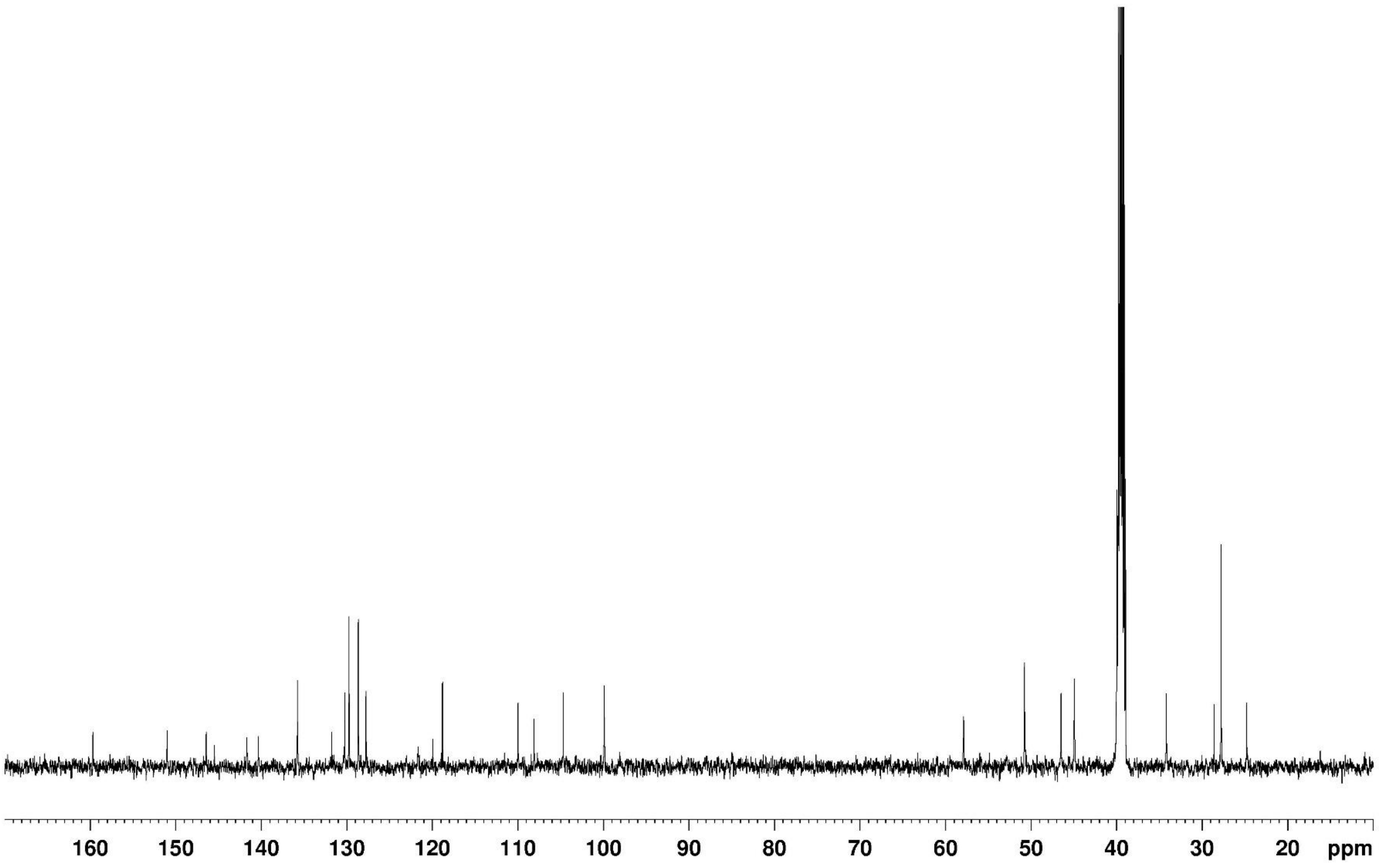

Figure S36 – ^{13}C NMR spectrum of **A4**

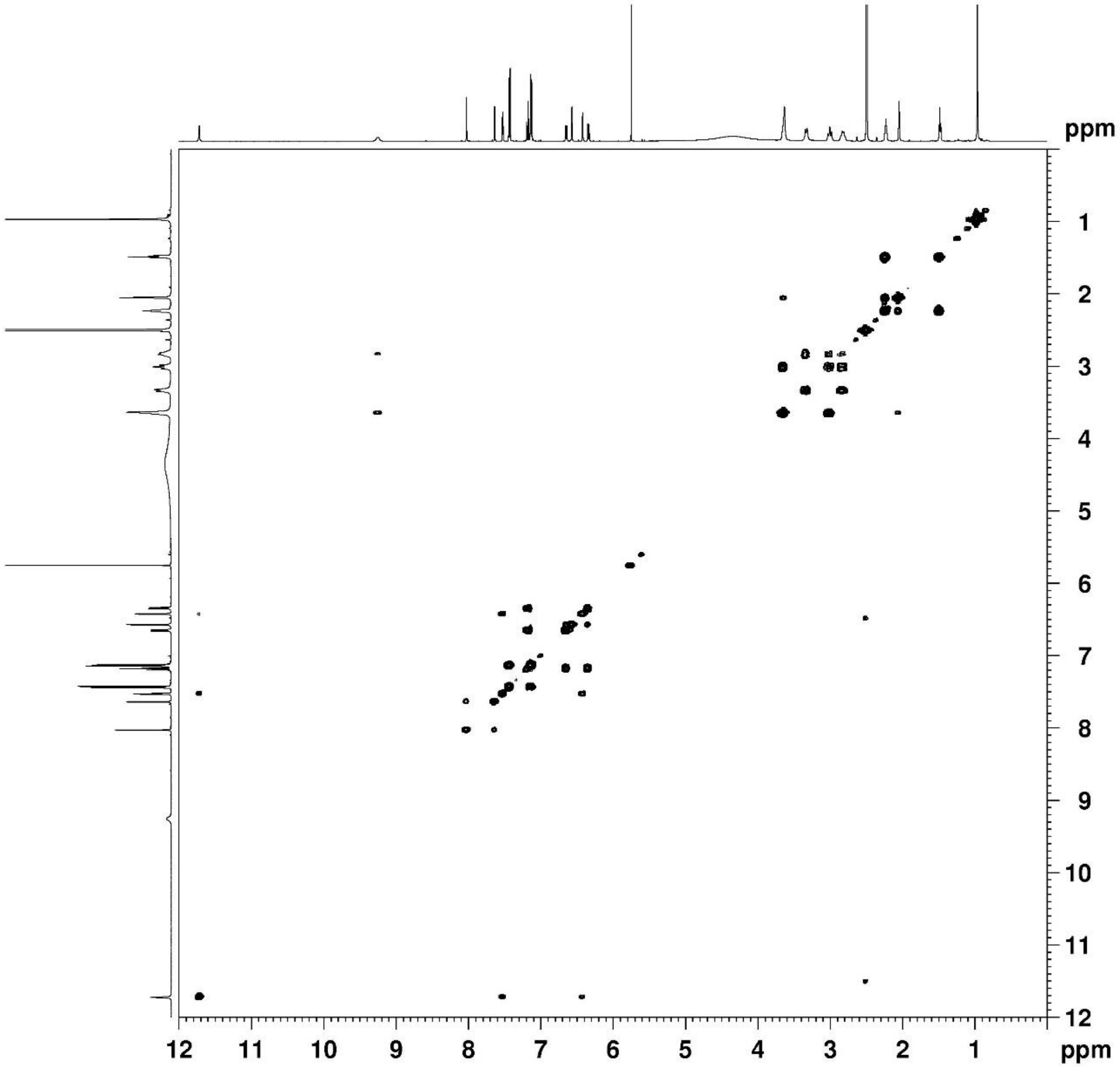

Figure S37 – (^{1}H, ^{1}H)-COSY spectrum of **A4**

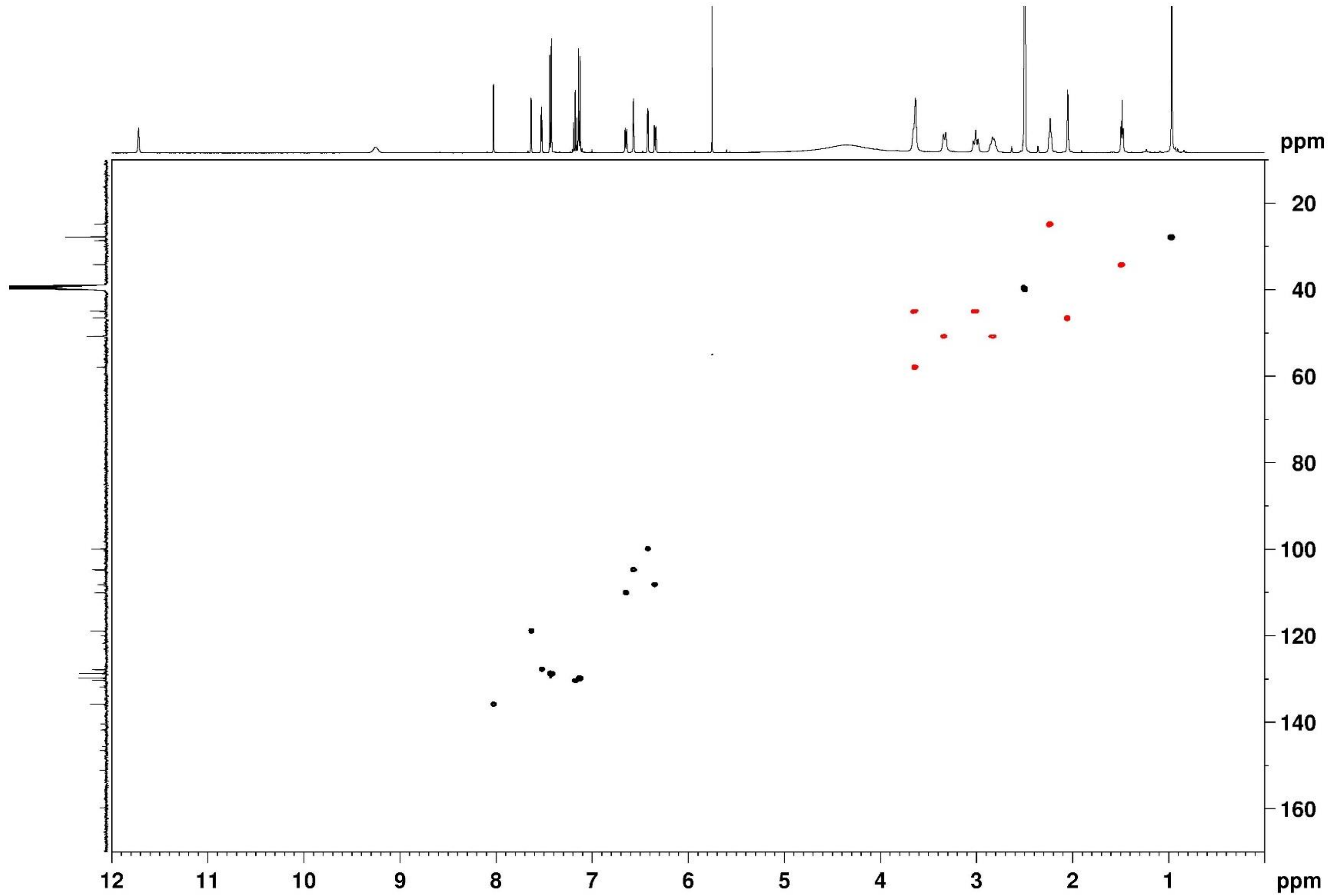

Figure S38 – (^{1}H, ^{13}C)-HSQC spectrum of **A4**

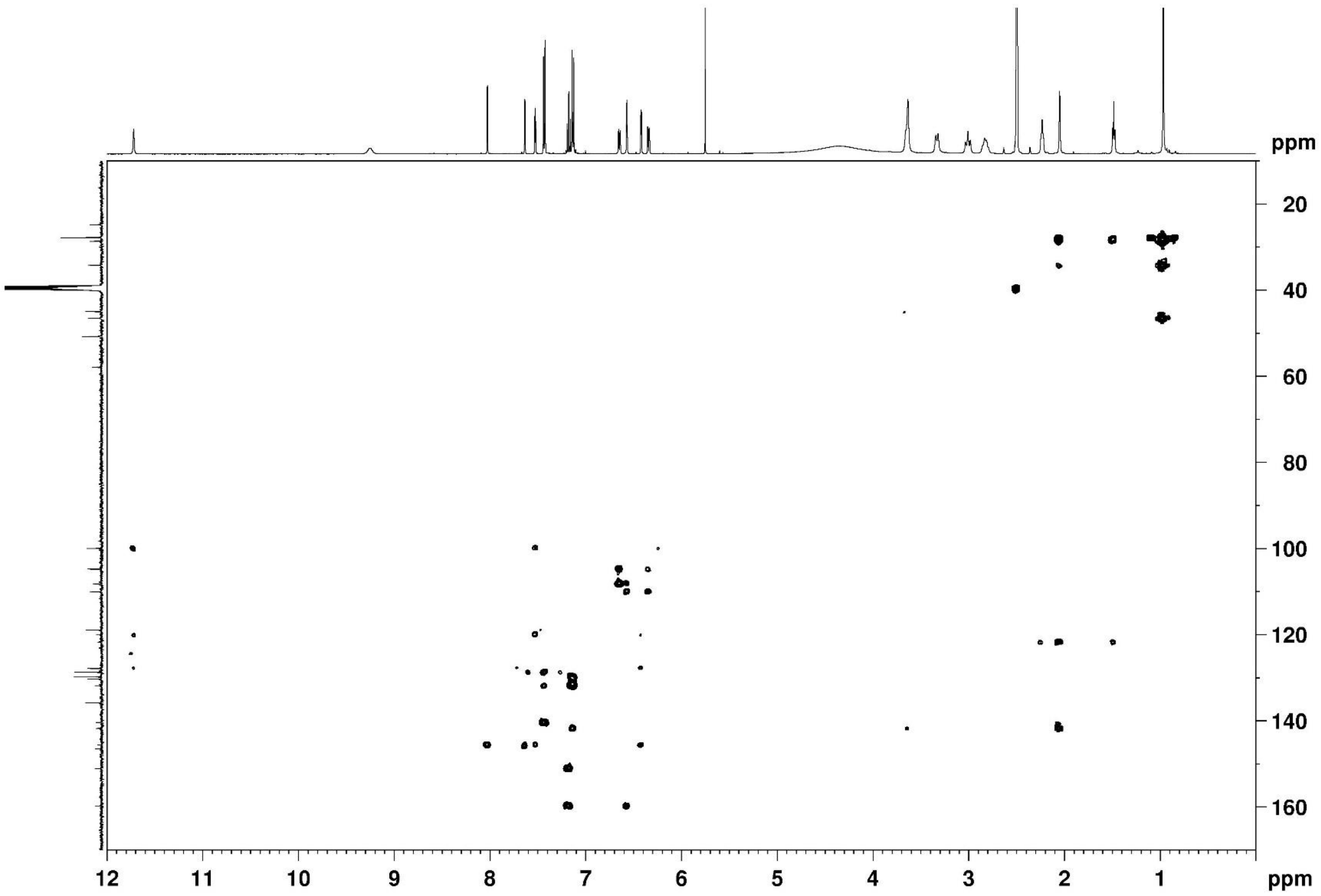

Figure S39 – (^{1}H, ^{13}C)-HMBC spectrum of **A4**

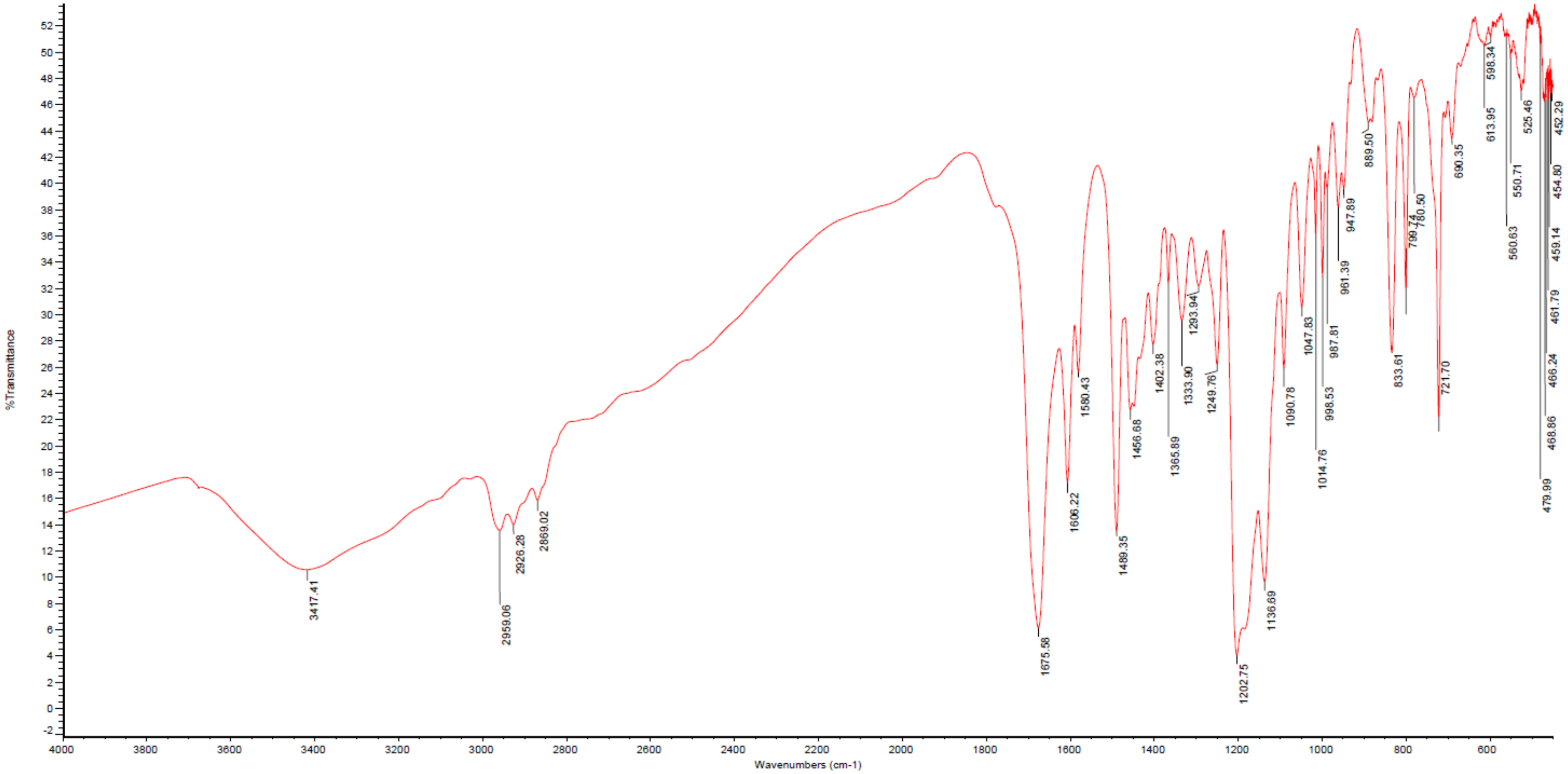

Figure S40 – IR spectrum of **A4**

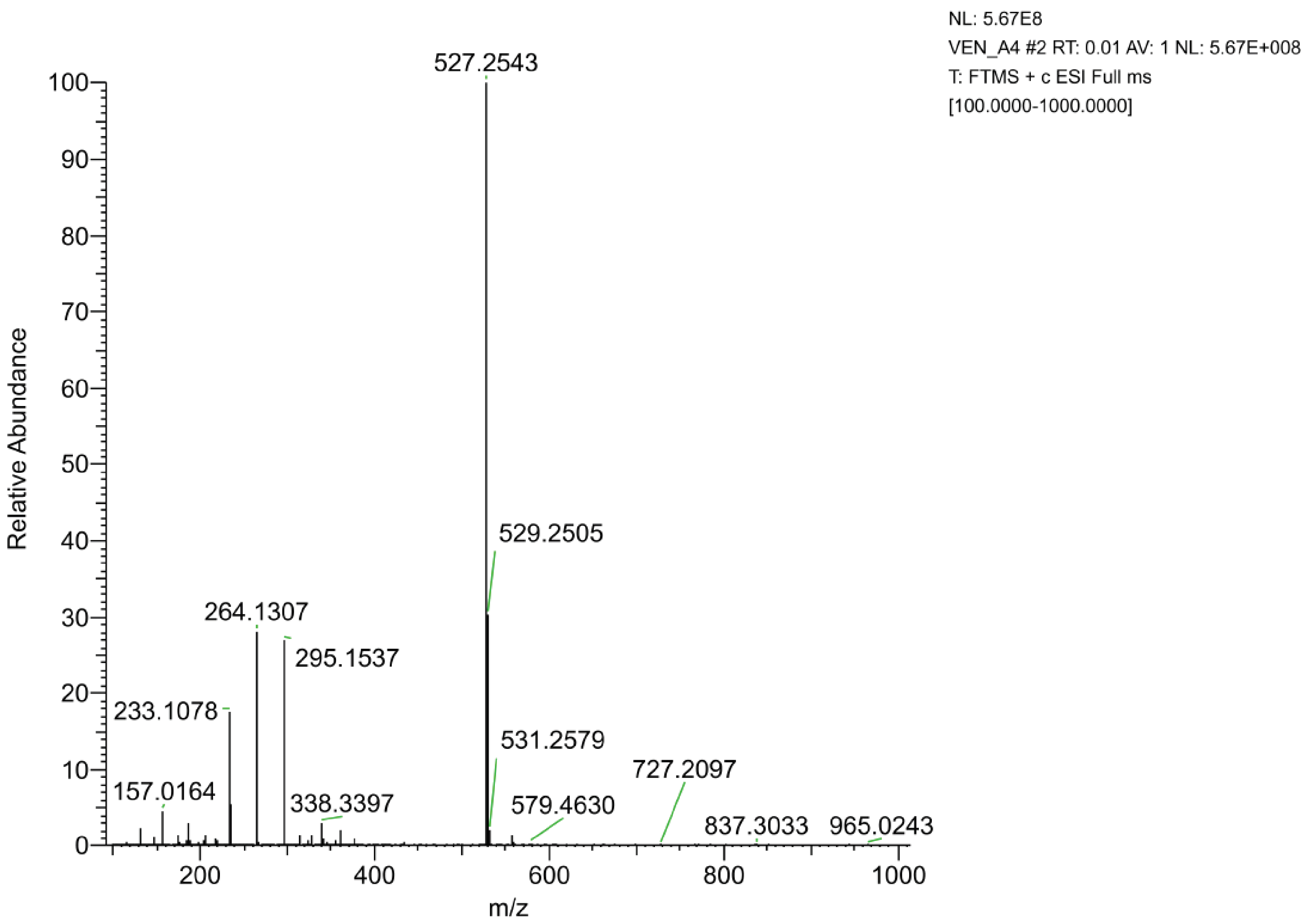

Figure S41 – HRMS spectrum of **A4**

Degradation product B1 information

5-(N-(2-((1*H*-pyrrolo[2,3-b]pyridin-5-yl)oxy)-4-(4-((4'-chloro-5,5-dimethyl-3,4,5,6-tetrahydro-[1,1'-biphenyl]-2-yl)methyl)piperazin-1-yl)benzoyl)sulfamoyl)-2-(tetrahydro-2*H*-pyran-4-yl)-1*H*-benzo[d]imidazole 3-oxide

Tautomer:
2-((1*H*-pyrrolo[2,3-b]pyridin-5-yl)oxy)-4-(4-((4'-chloro-5,5-dimethyl-3,4,5,6-tetrahydro-[1,1'-biphenyl]-2-yl)methyl)piperazin-1-yl)-*N*-((1-hydroxy-2-(tetrahydro-2*H*-pyran-4-yl)-1*H*-benzo[d]imidazol-6-yl)sulfonyl)benzamide: amorphous solid; HRMS $[M+H]^+$: calculated 850.3148, found 850.3132.

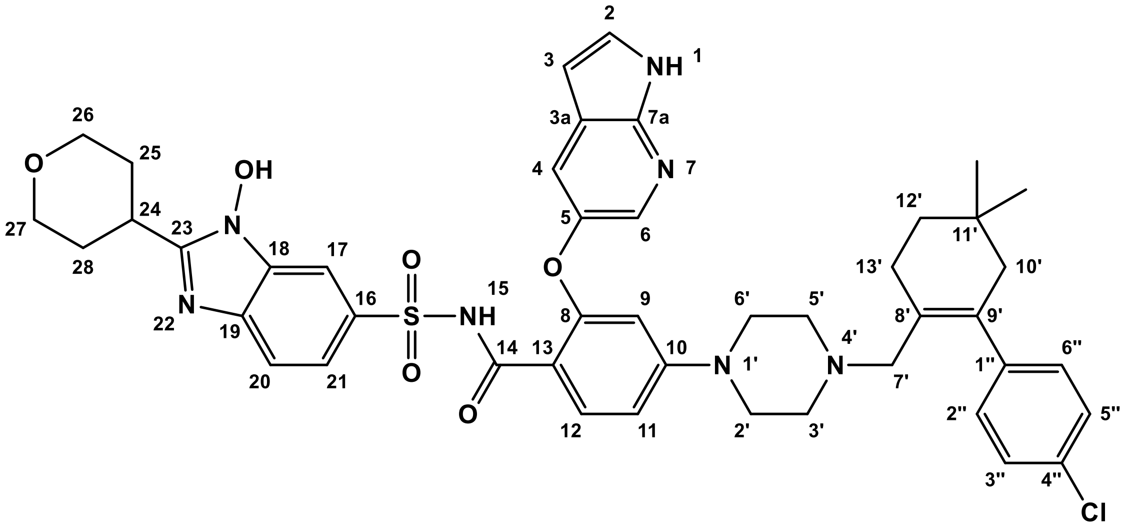

Figure S42 – Degradation product B1 with NMR assignations

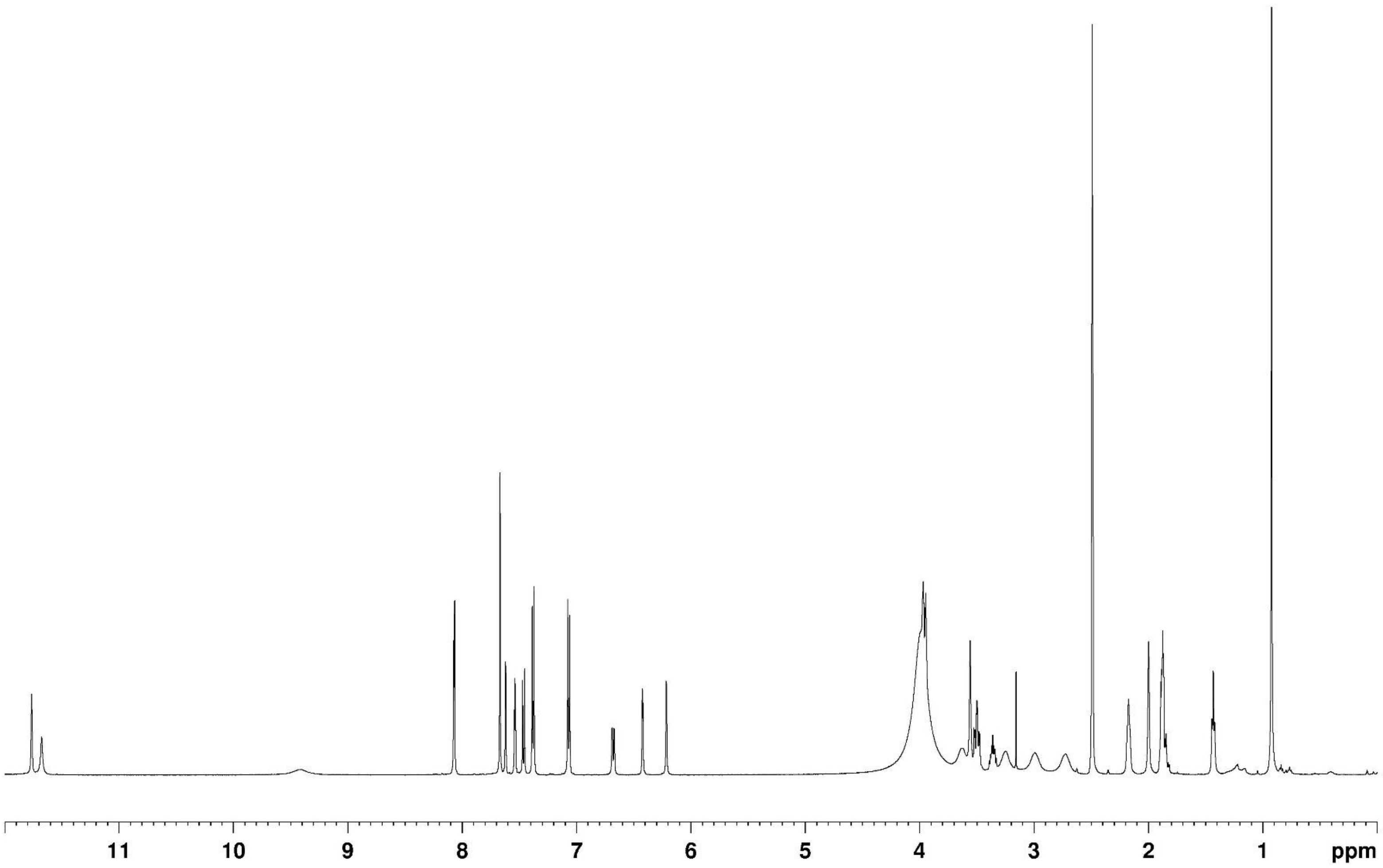

Figure S43 – ^{1}H NMR spectrum of **B1**

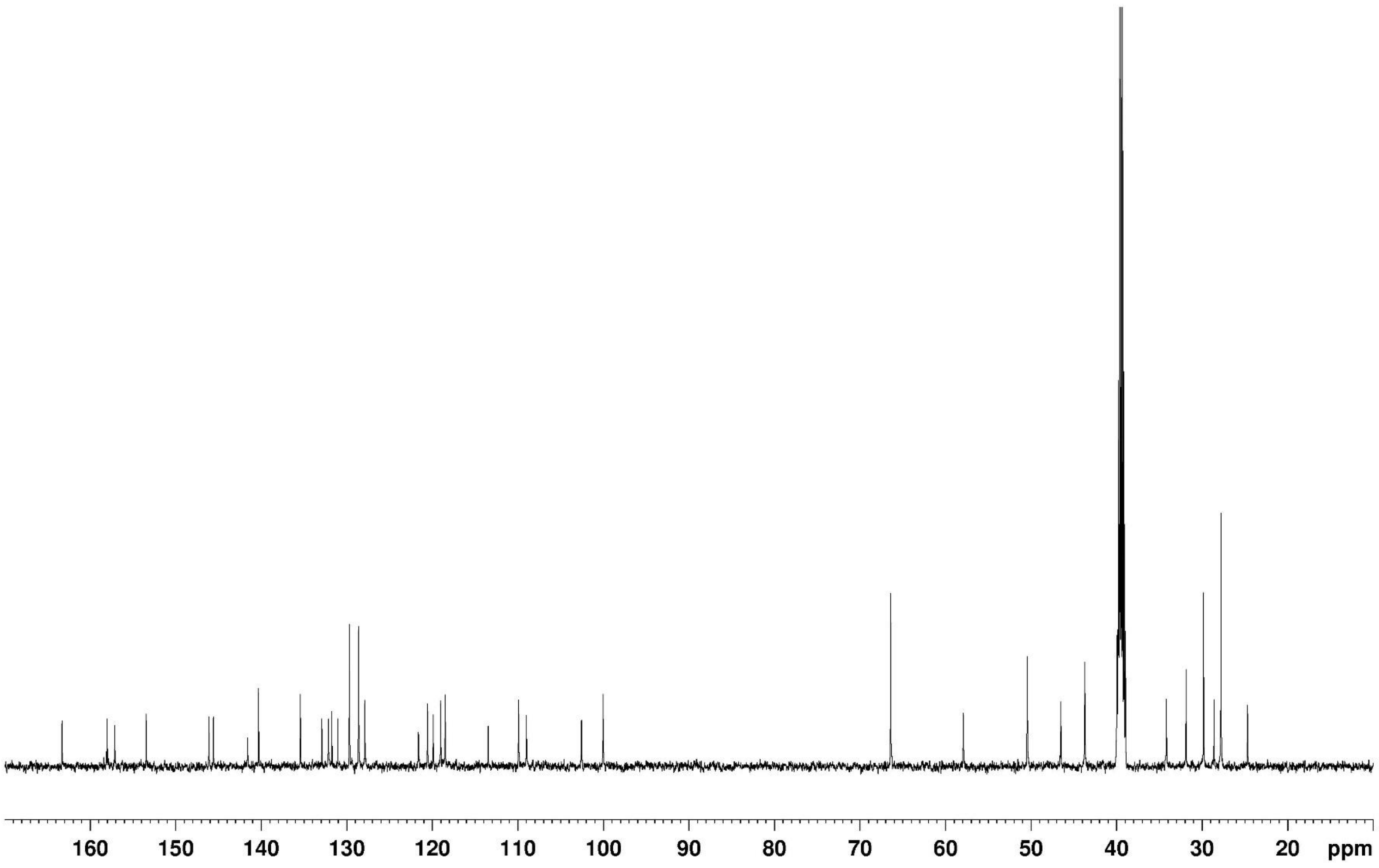

Figure S44 – ^{13}C NMR spectrum of **B1**

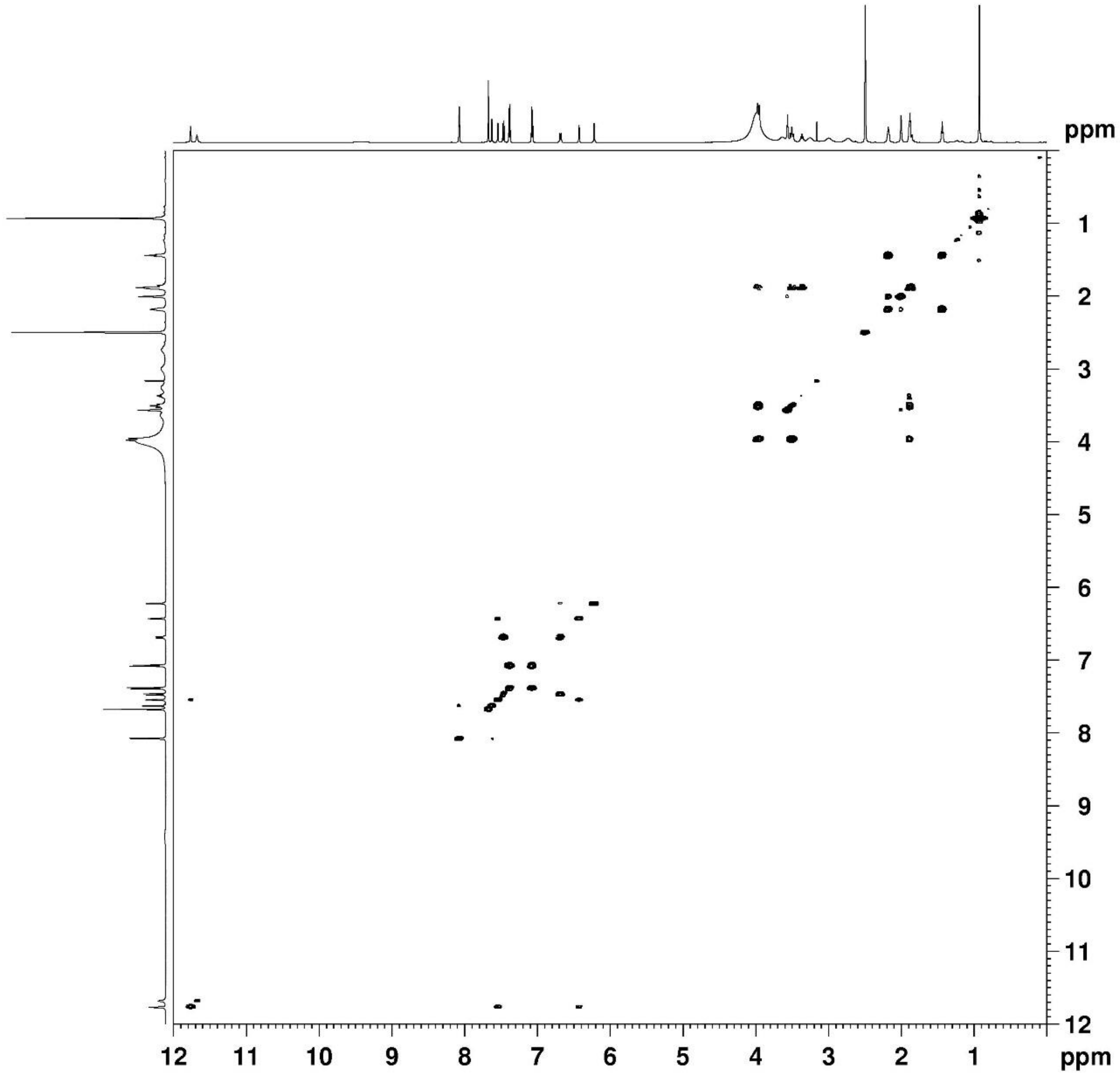

Figure S45 – (^{1}H, ^{1}H)-COSY spectrum of **B1**

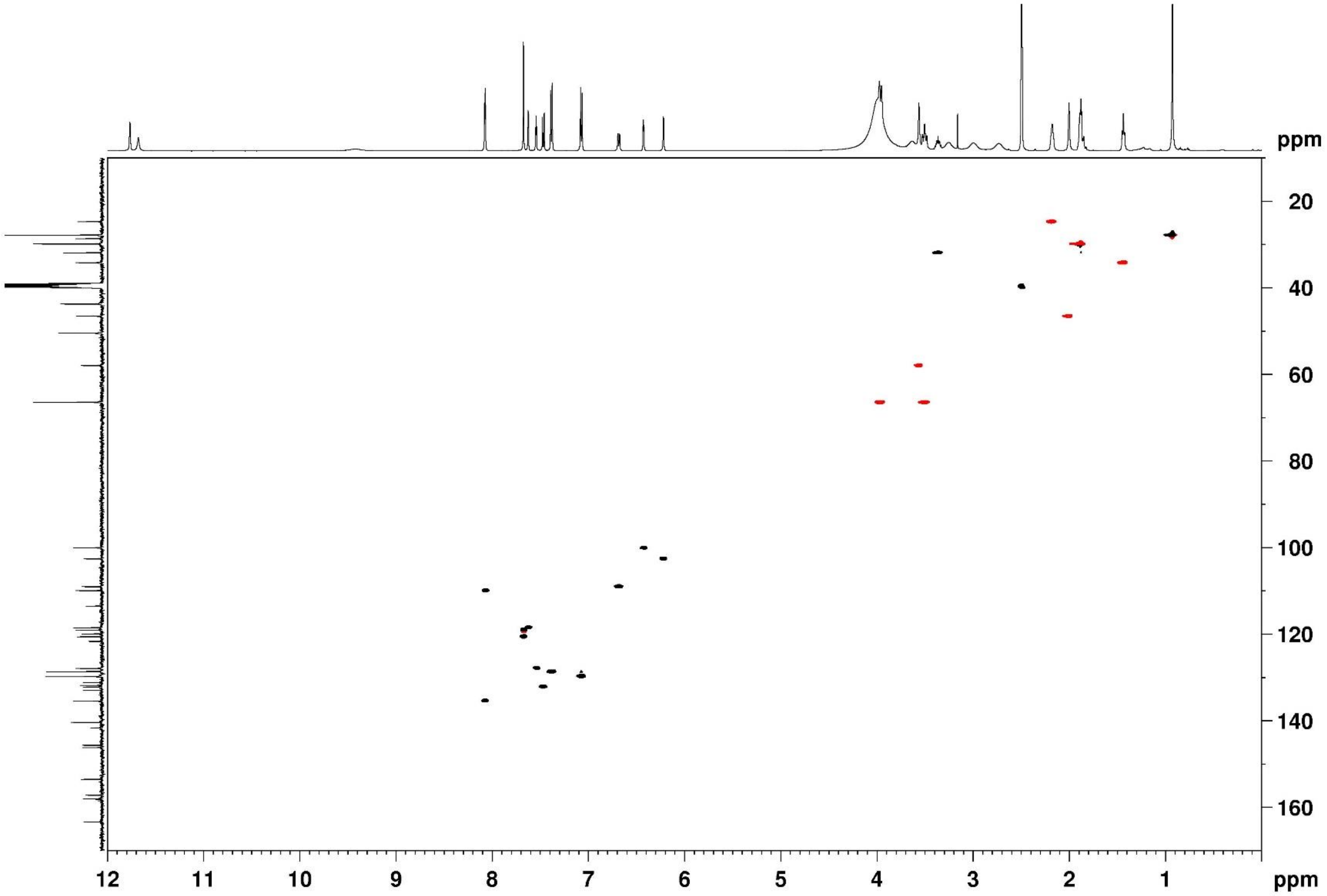

Figure S46 – (^{1}H, ^{13}C)-HSQC spectrum of **B1**

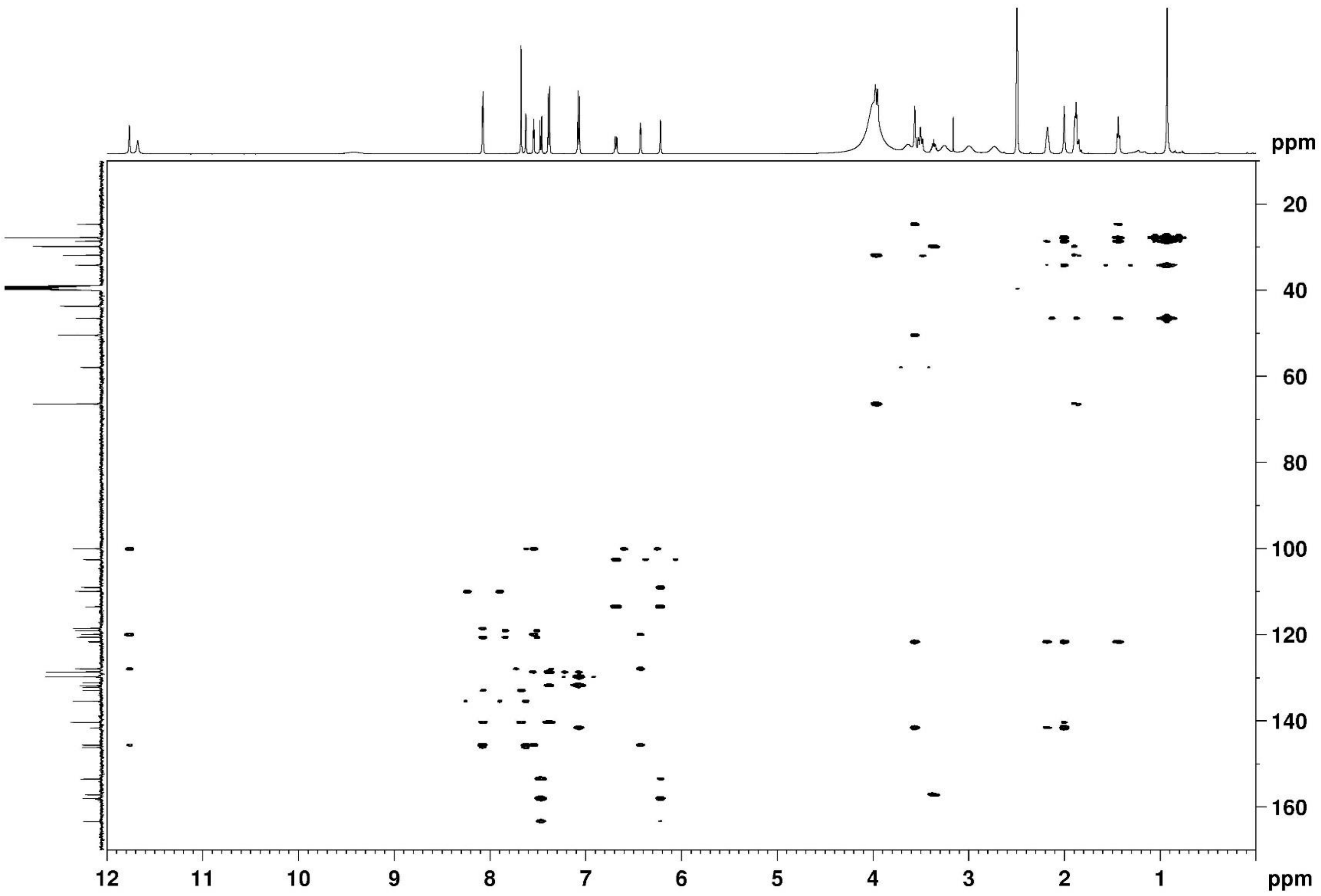

Figure S47 – (^{1}H, ^{13}C)-HMBC spectrum of **B1**

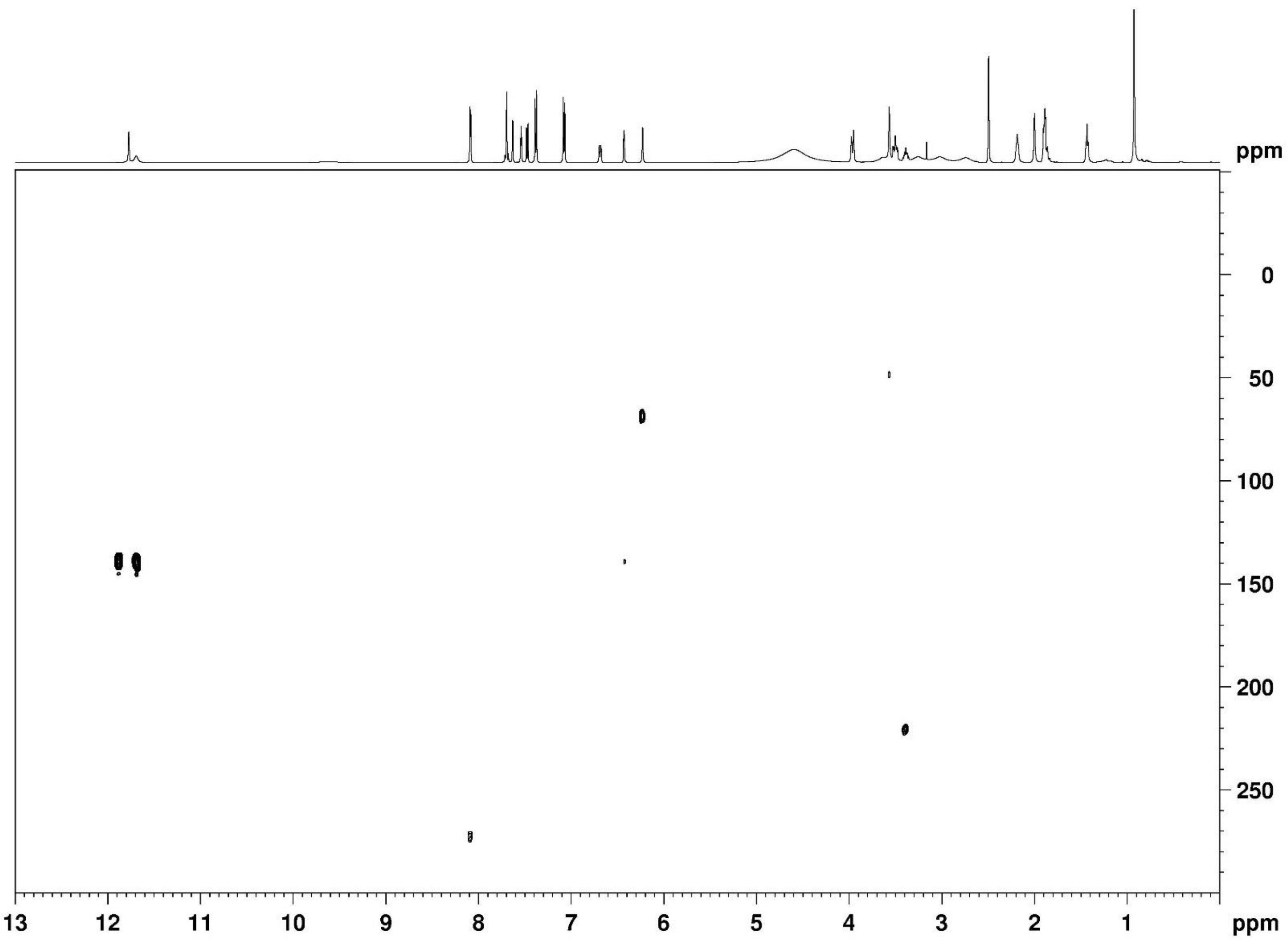

Figure S48 – (^{1}H, ^{15}N)-HMBC spectrum of **B1**

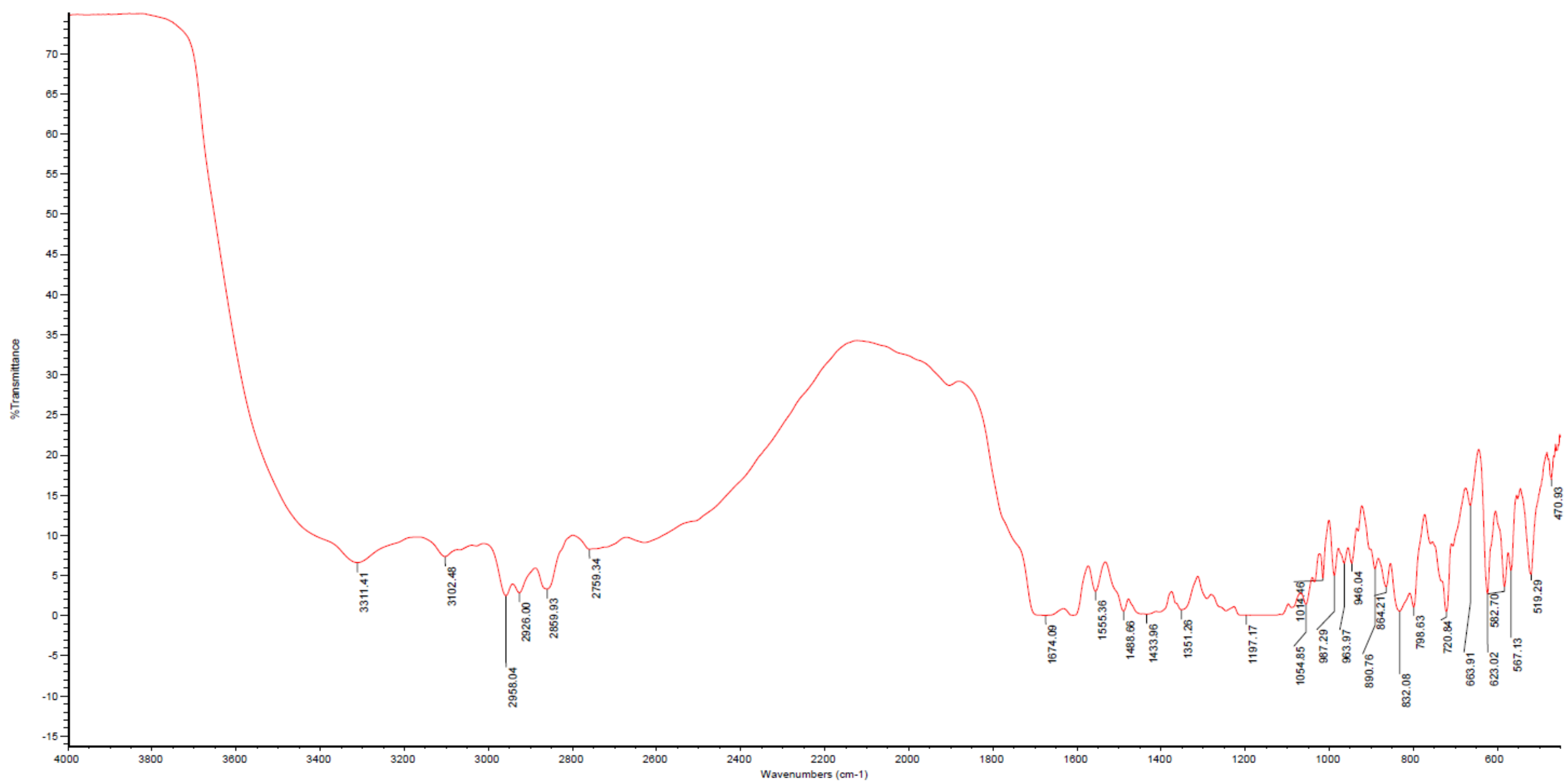

Figure S49 – IR spectrum of **B1**

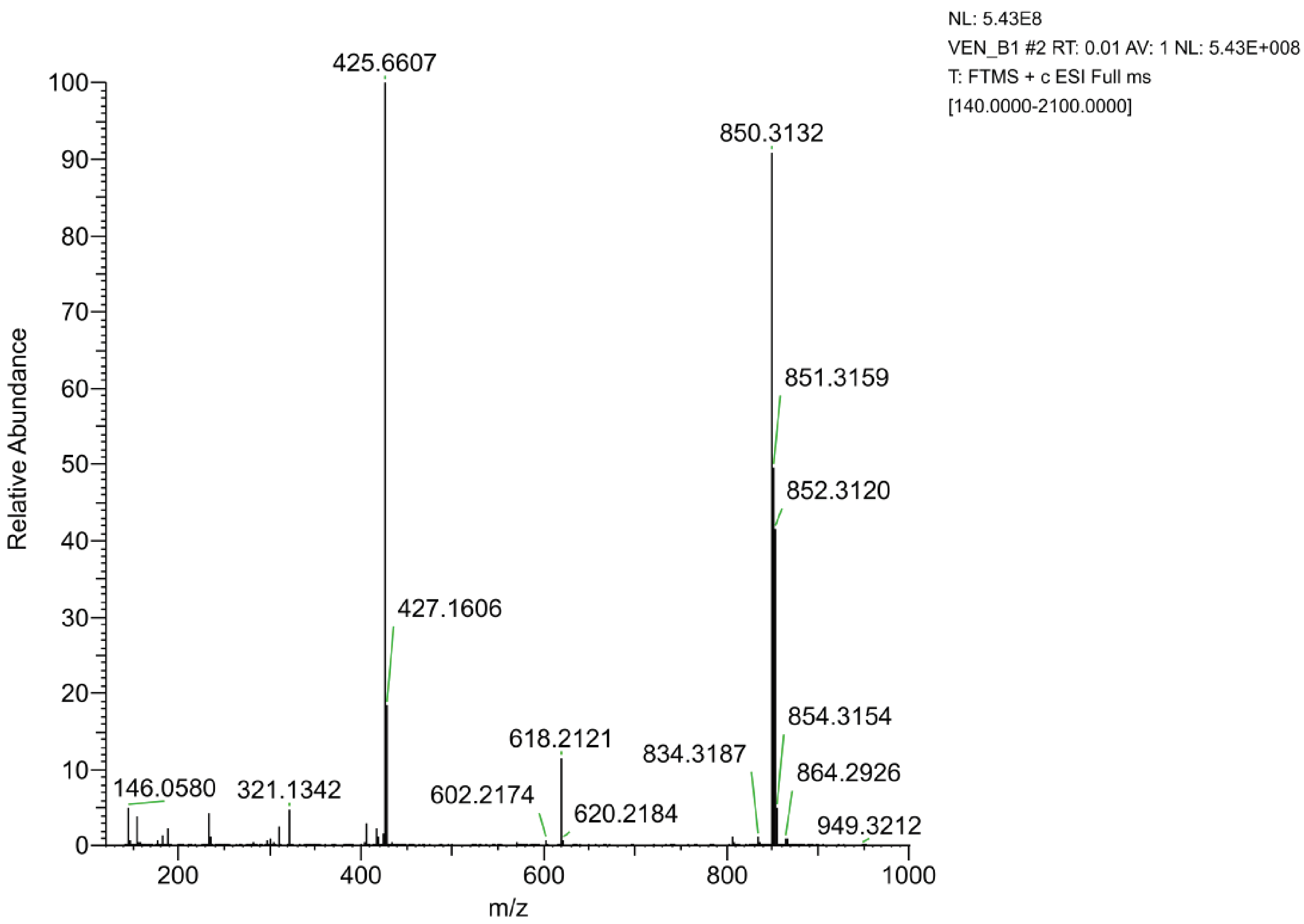

Figure S50 – HRMS spectrum of **B1**

Degradation product B2 information

2-((1*H*-pyrrolo[2,3-b]pyridin-5-yl)oxy)-4-(4-((4'-chloro-5,5-dimethyl-3,4,5,6-tetrahydro-[1,1'-biphenyl]-2-yl)methyl)piperazin-1-yl)-*N*-((4-hydroxy-3-nitrophenyl)sulfonyl)benzamide: HRMS $[M+H]^+$: calculated 771.2362, found 771.2351.

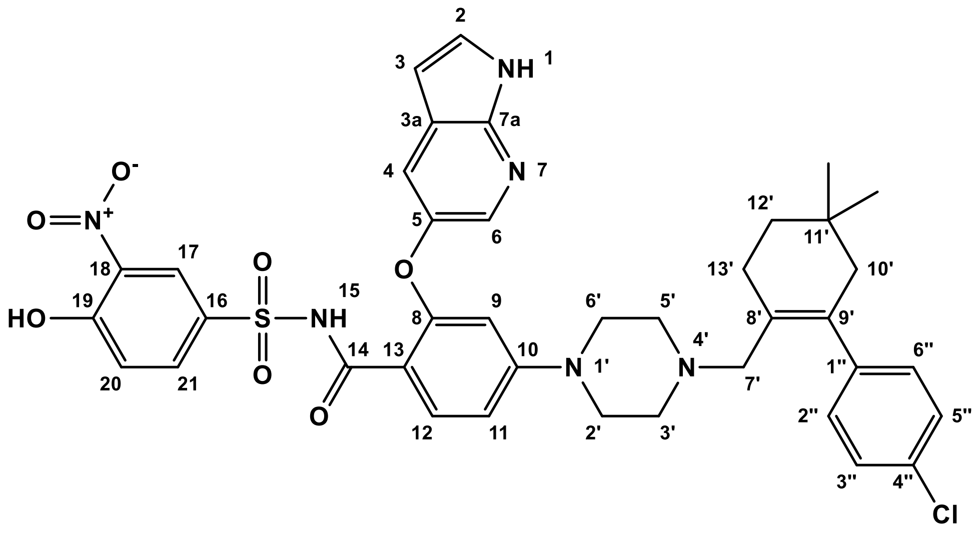

Figure S51 – Degradation product B2 with NMR assignations

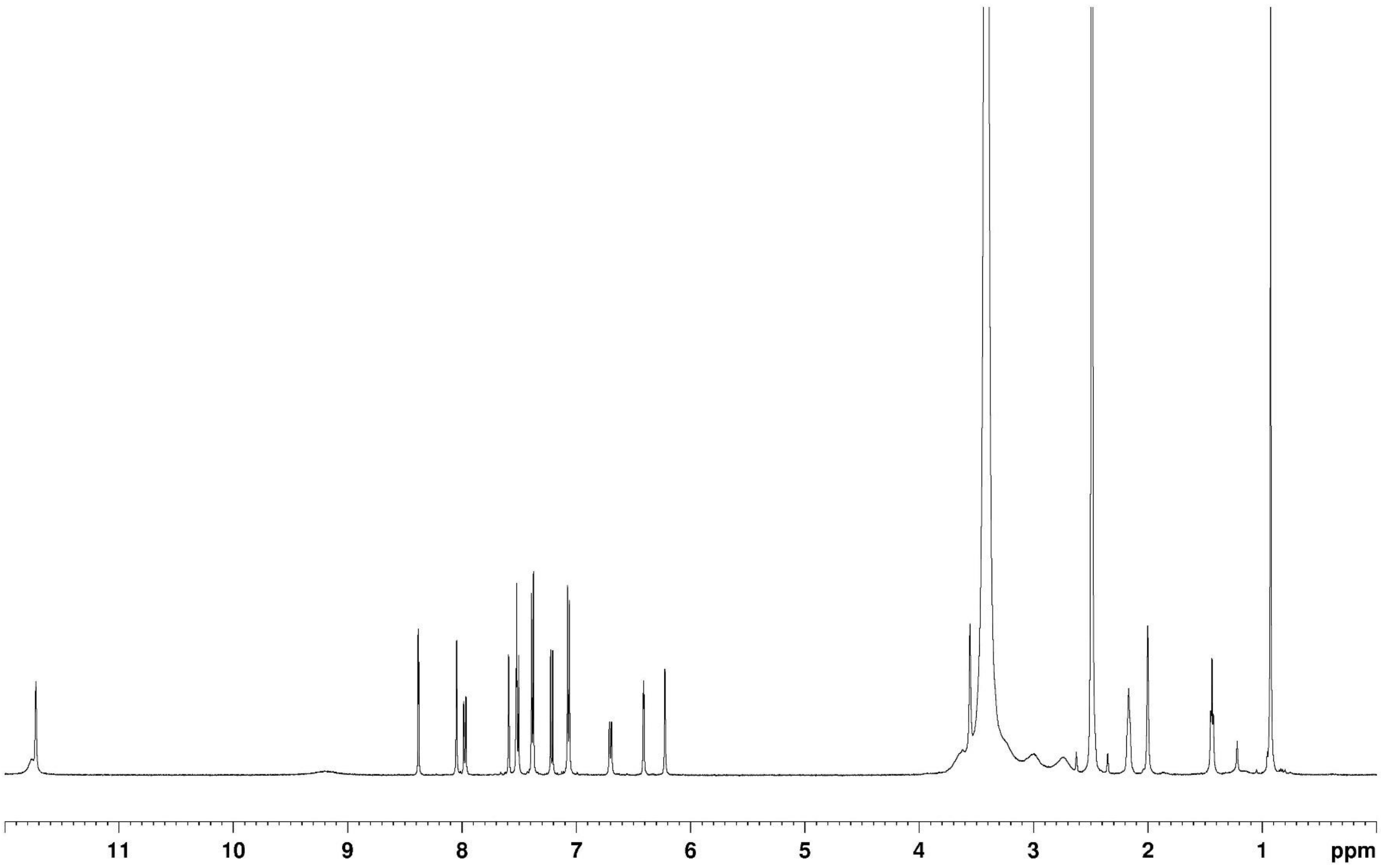

Figure S52 – ^{1}H NMR spectrum of **B2**

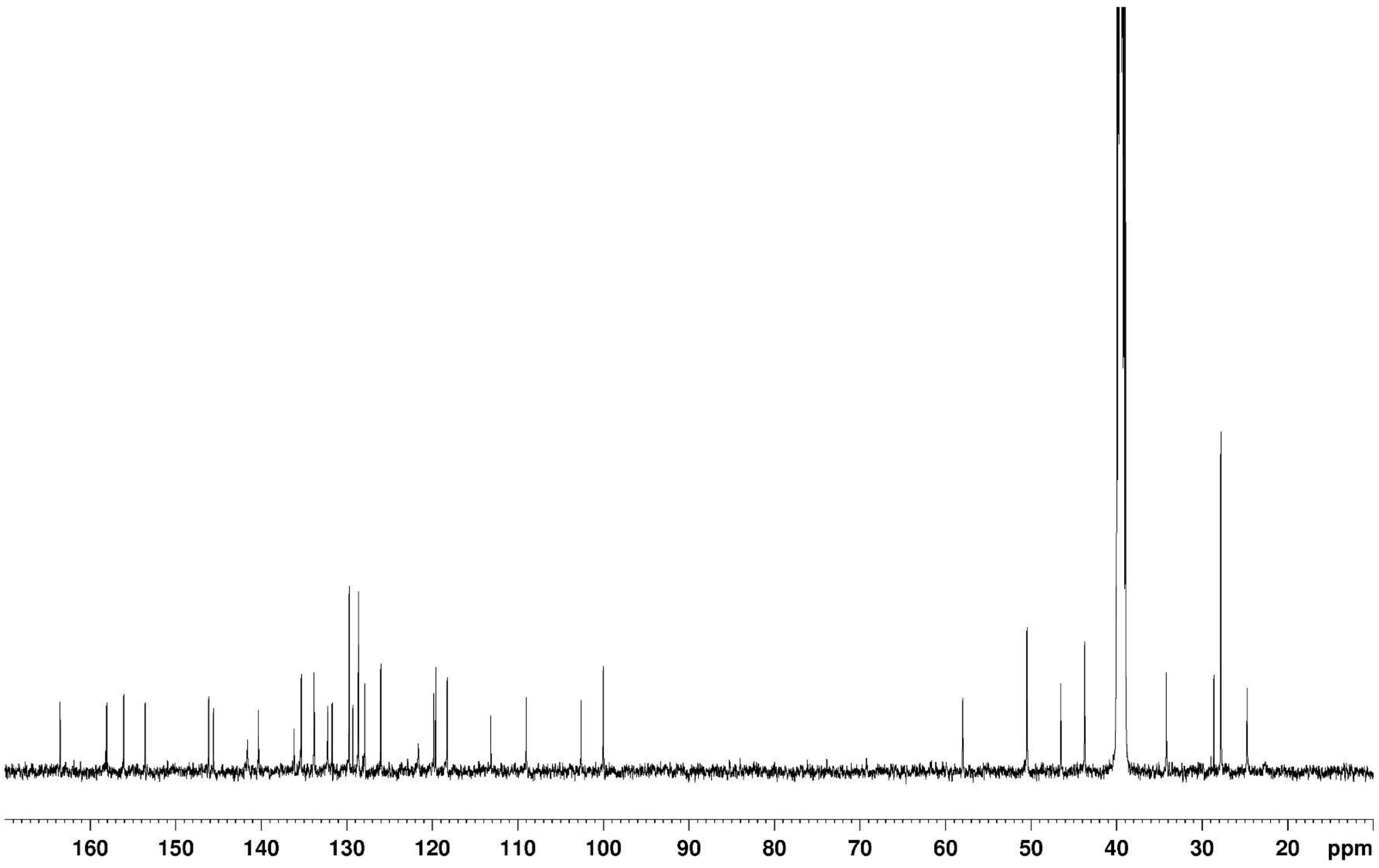

Figure S53 – ^{13}C NMR spectrum of **B2**

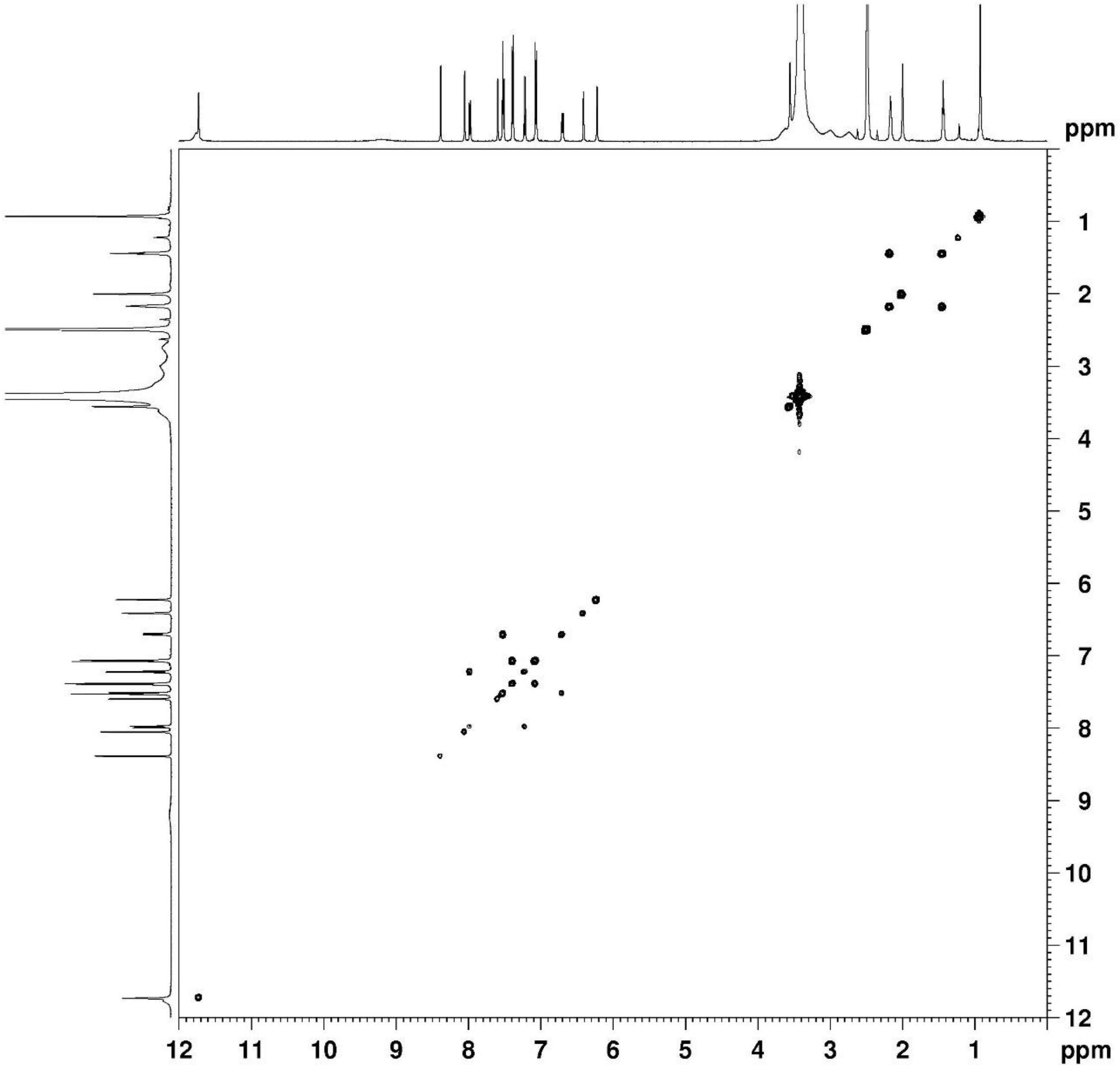

Figure S54 – (^{1}H, ^{1}H)-COSY spectrum of **B2**

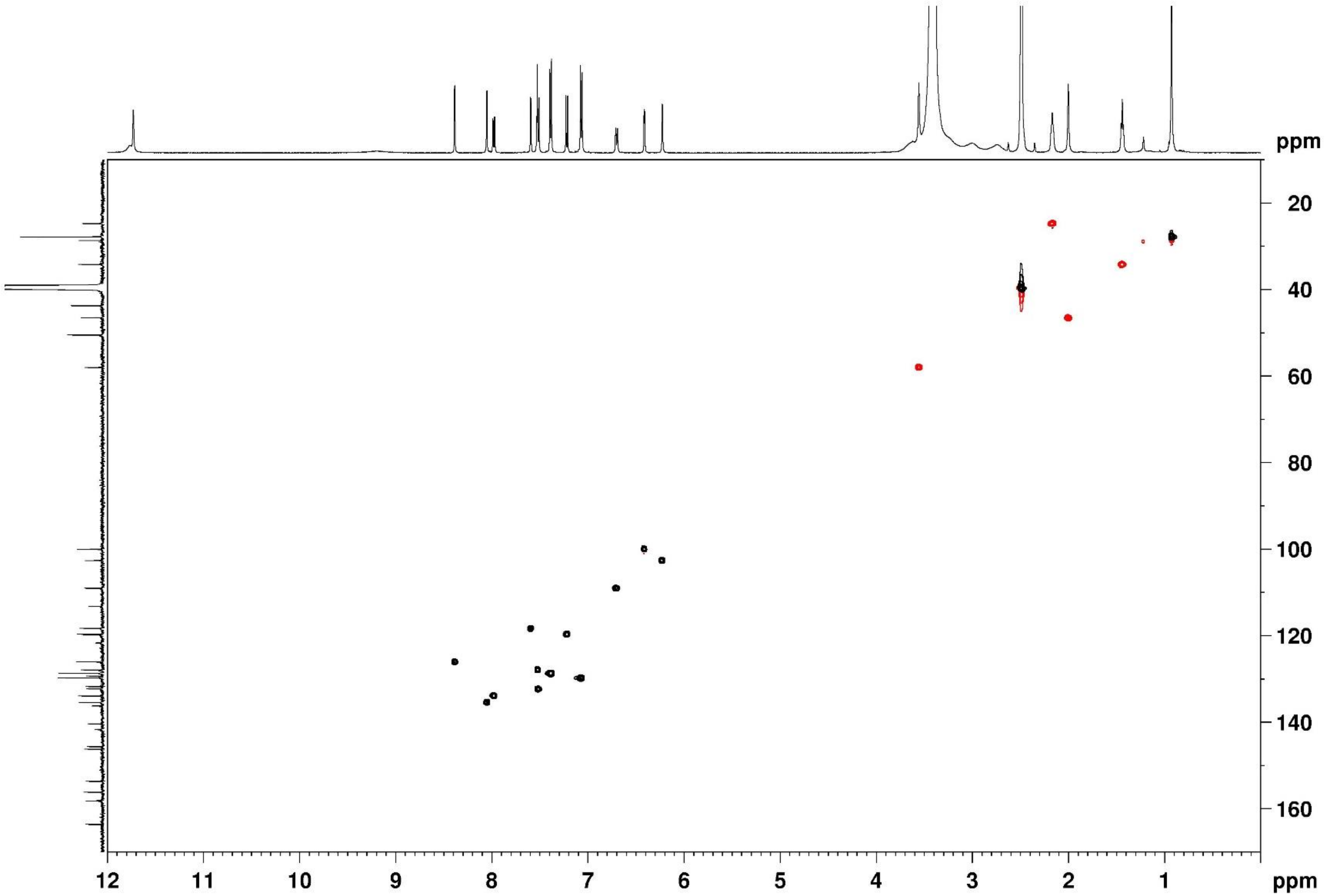

Figure S55 – (^{1}H, ^{13}C)-HSQC spectrum of **B2**

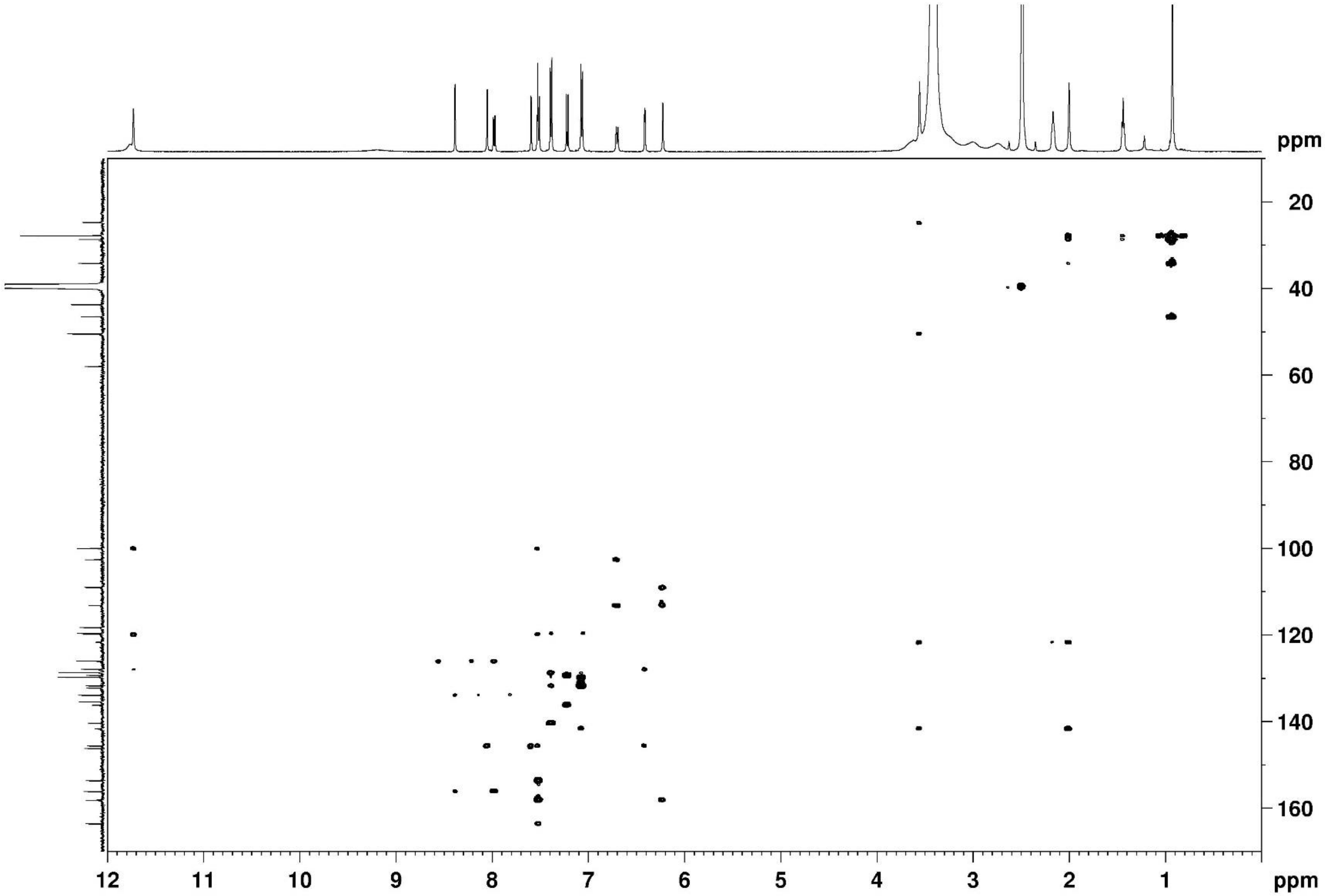

Figure S56 – (^{1}H, ^{13}C)-HMBC spectrum of **B2**

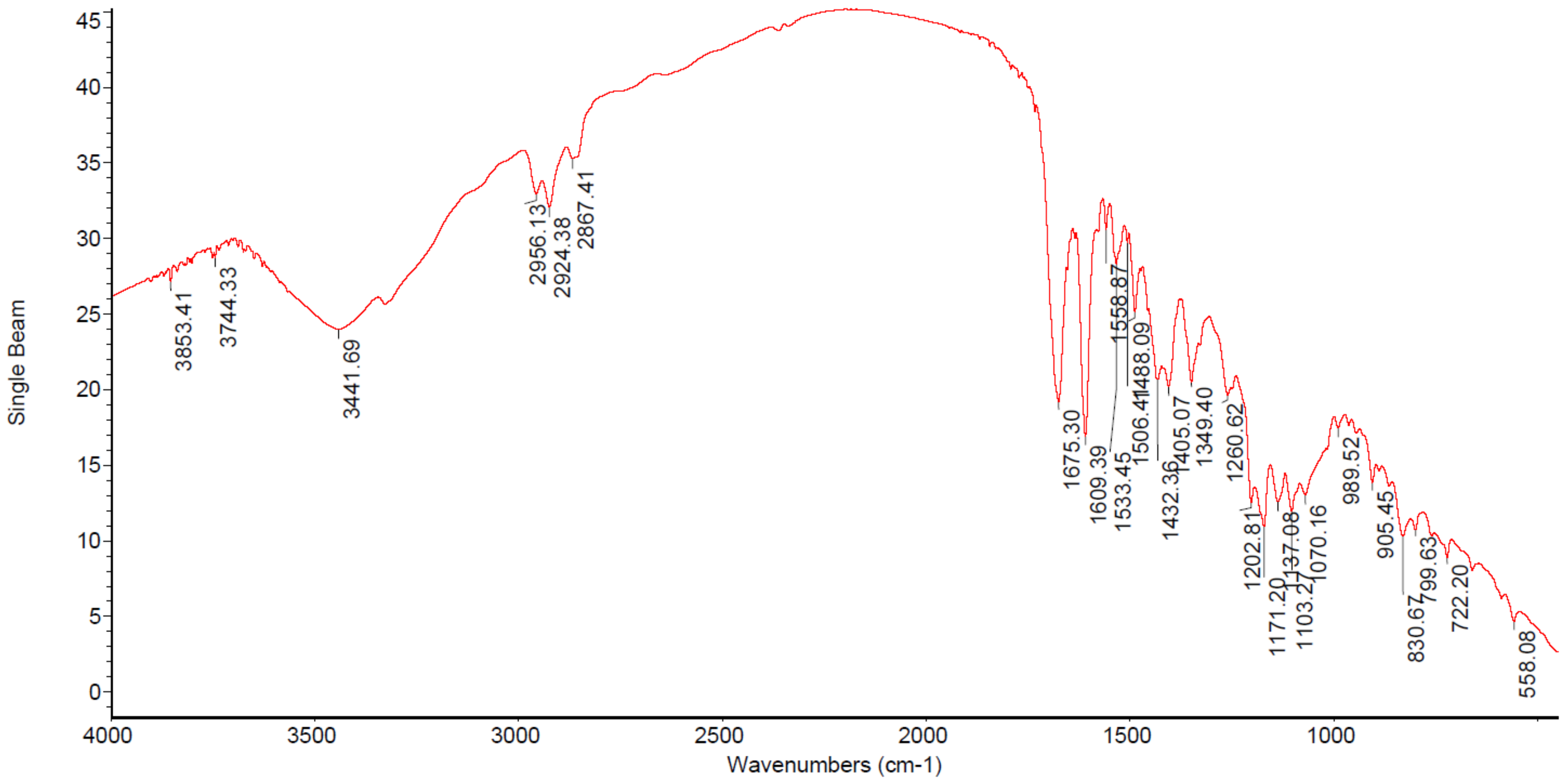

Figure S57 – IR spectrum of **B2**

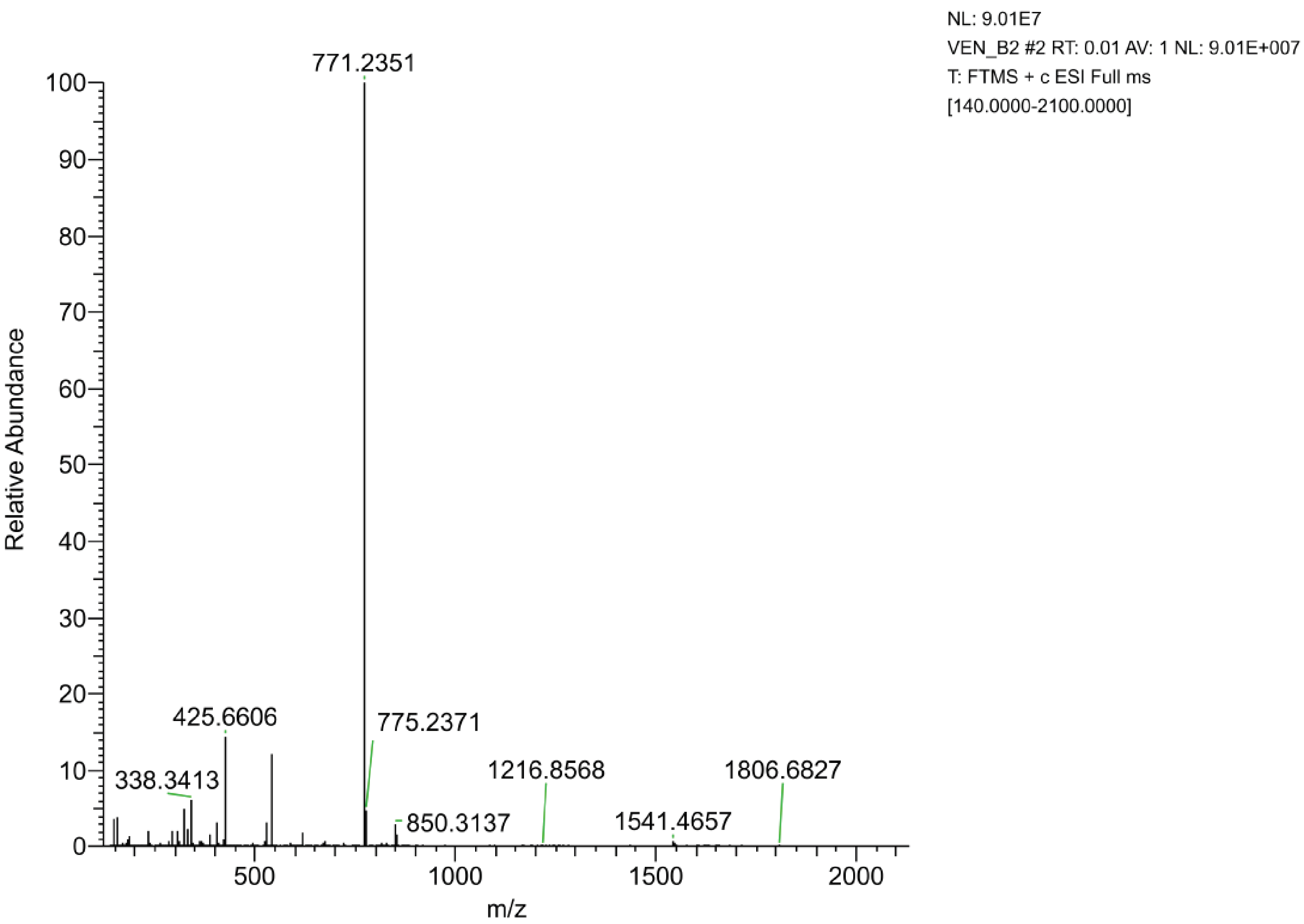

Figure S58 – HRMS spectrum of **B2**

Degradation product B3 information (see Figure S25 – Degradation product A3)

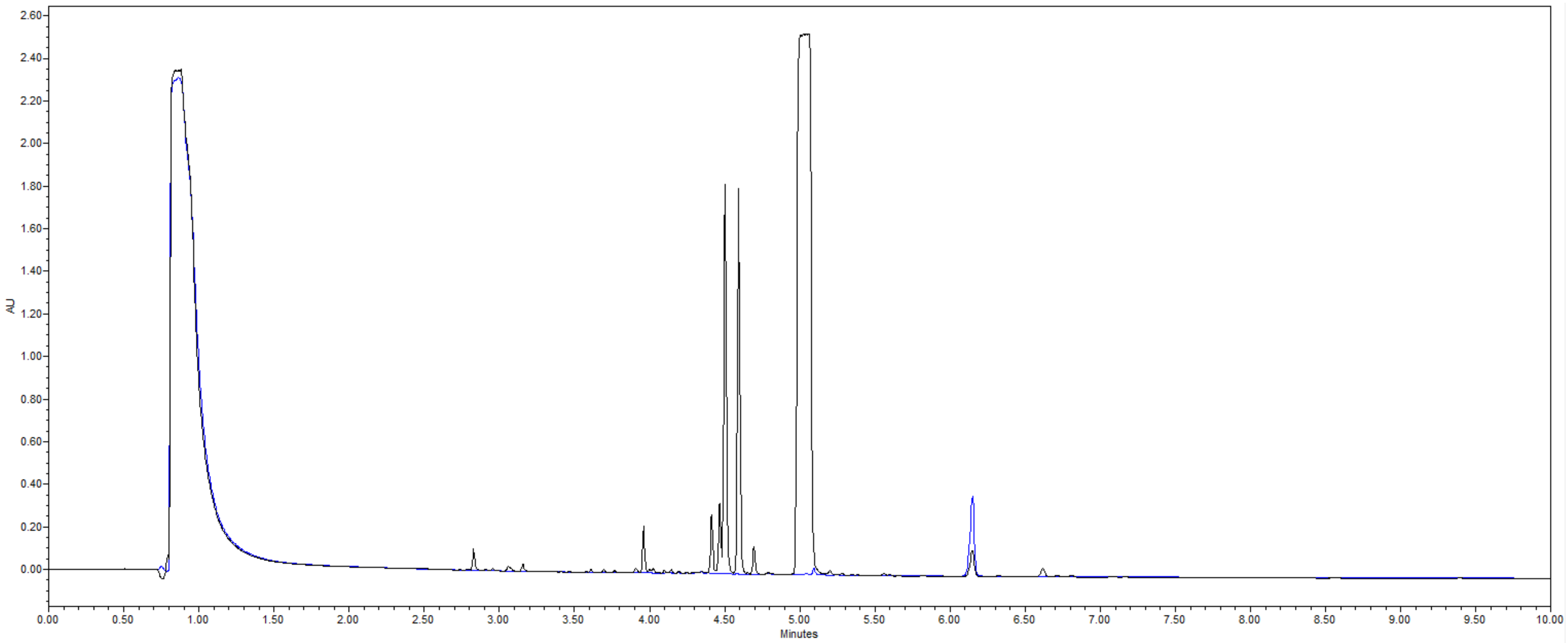

Figure S59 – Overlay chromatogram of a stress sample of venetoclax with added 1M NaOH after 1 day at 50 °C (black) and degradation product A3 (blue).

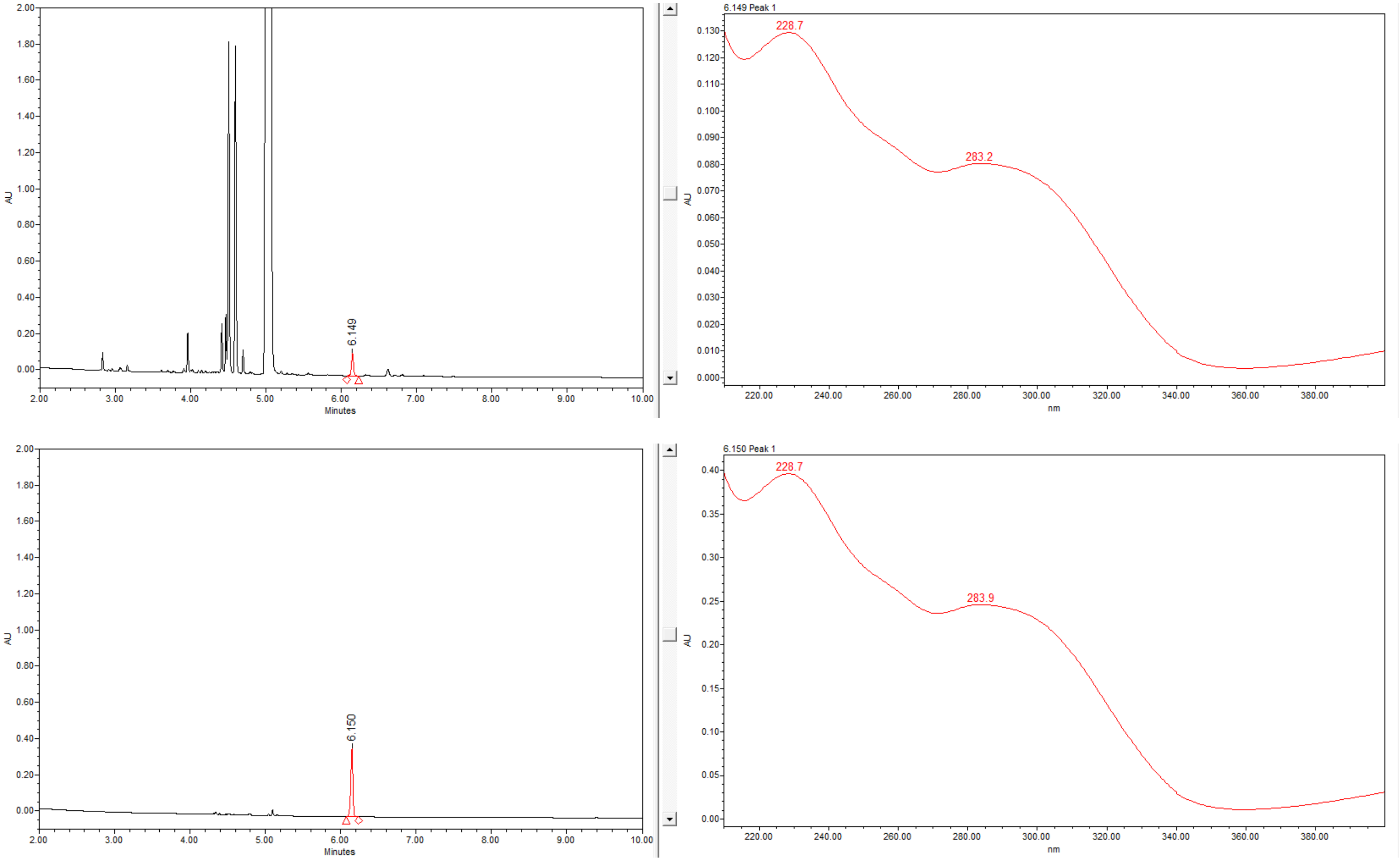

Figure S60 – Chromatograms (left) and UV spectra (right) of **degradation product B3** in stress sample of venetoclax with added 1M NaOH after 1 day at 50 °C (top) and **degradation product A3** (bottom)

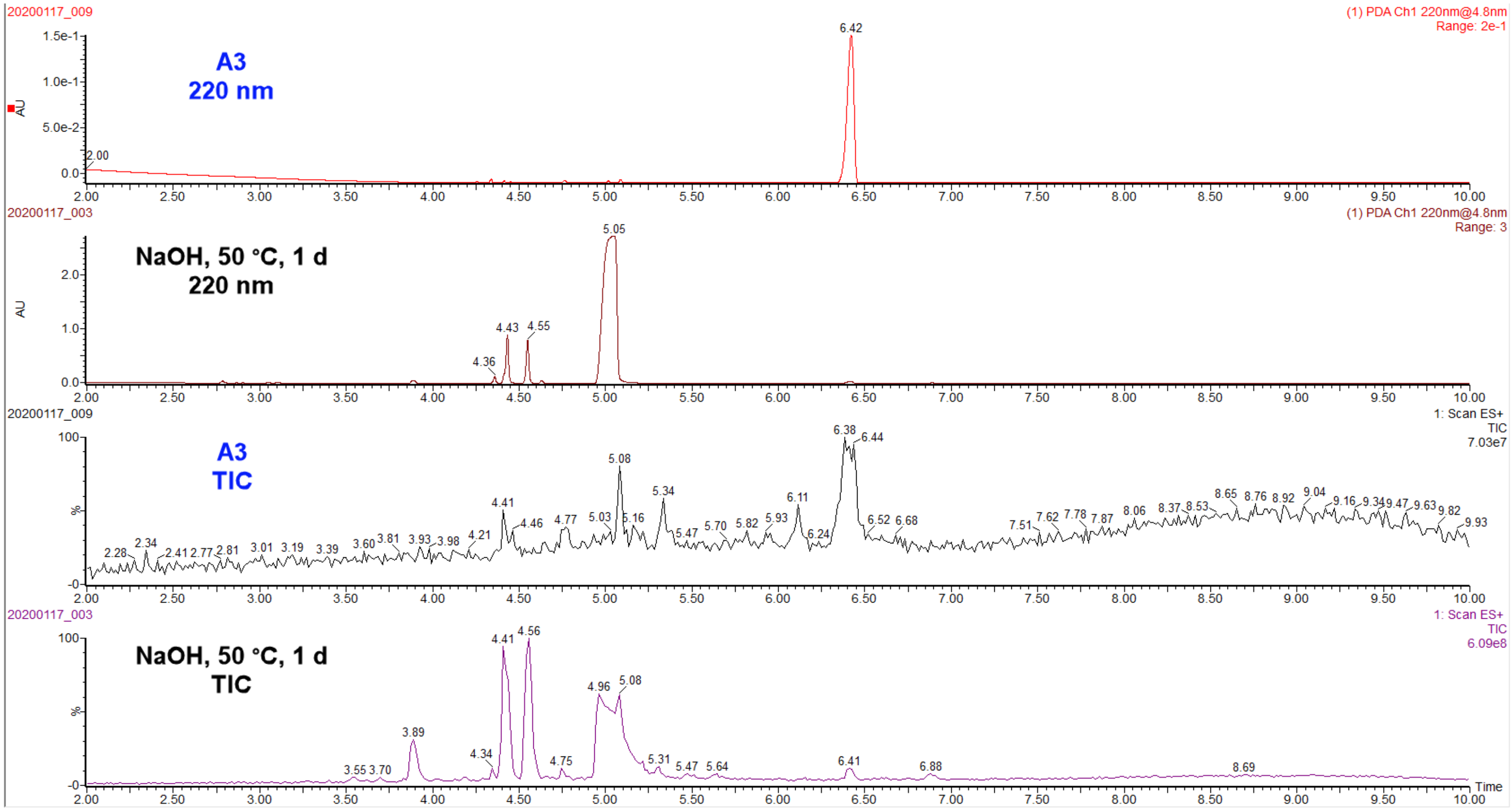

Figure S61 – Chromatograms of a stress sample of venetoclax with added 1M NaOH after 1 day on 50 °C and degradation product A3 obtained with UV and MS detector

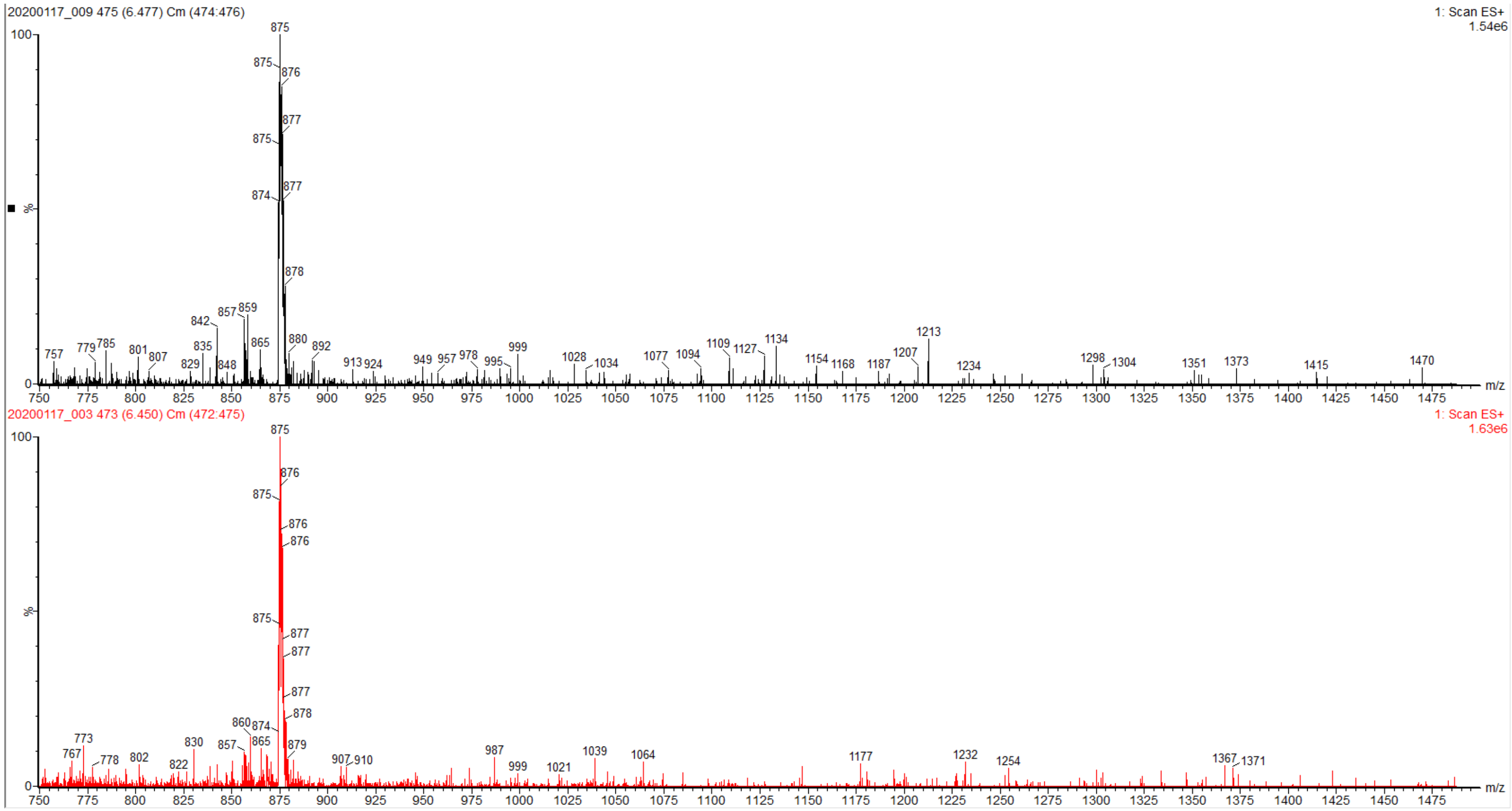

Figure S62 – MS spectra of **degradation product A3** (top) and **degradation product B3** (bottom).

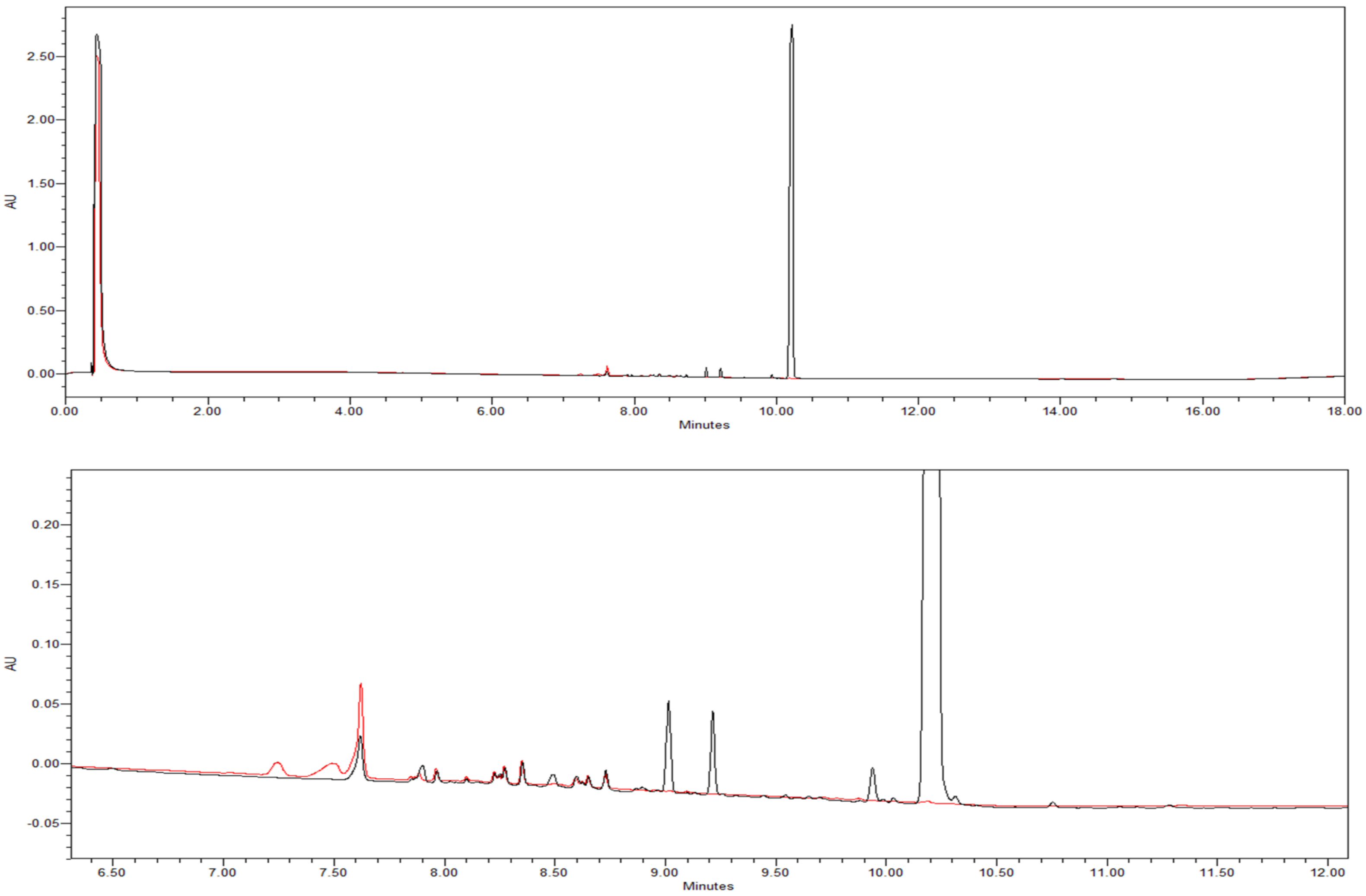

Figure S63 – Overlay chromatogram of a stress sample of venetoclax with added 3% H_2O2 after 7 days at 50 °C (black) and commercially obtained *N*-oxide venetoclax impurity (red) (top) and a close up of the same chromatogram from 6.3 to 12.1 min (bottom). The impurities eluting at approximately 9 min and 9.2 min are process related impurities present in the venetoclax substance.

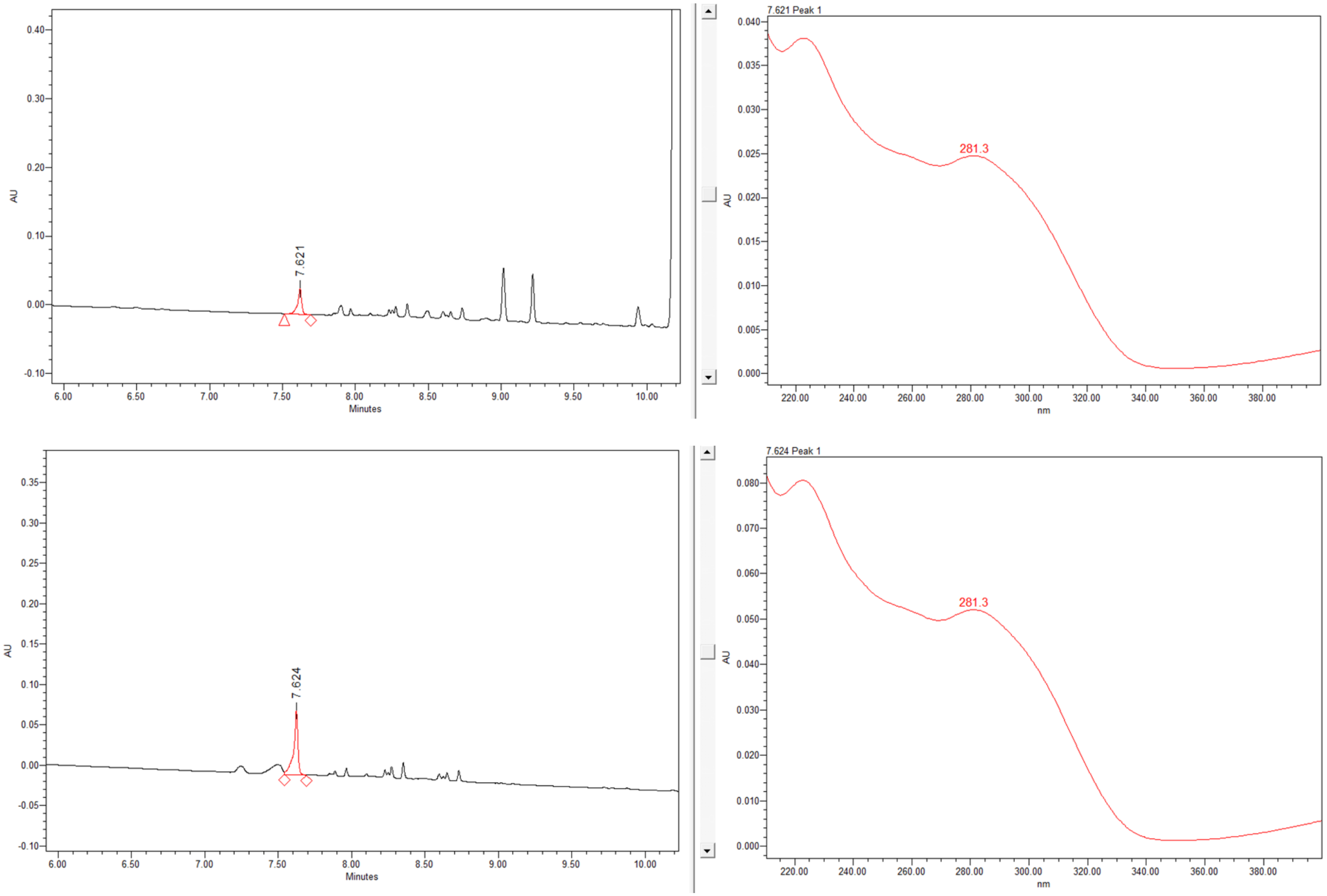

Figure S64 – Chromatograms (left) and UV spectra (right) of **degradation product *N*-oxide** in stress sample of venetoclax with added 3% H_2O_2 after 5 days at room temperature (top) and commercially obtained ***N*-oxide venetoclax** (bottom).

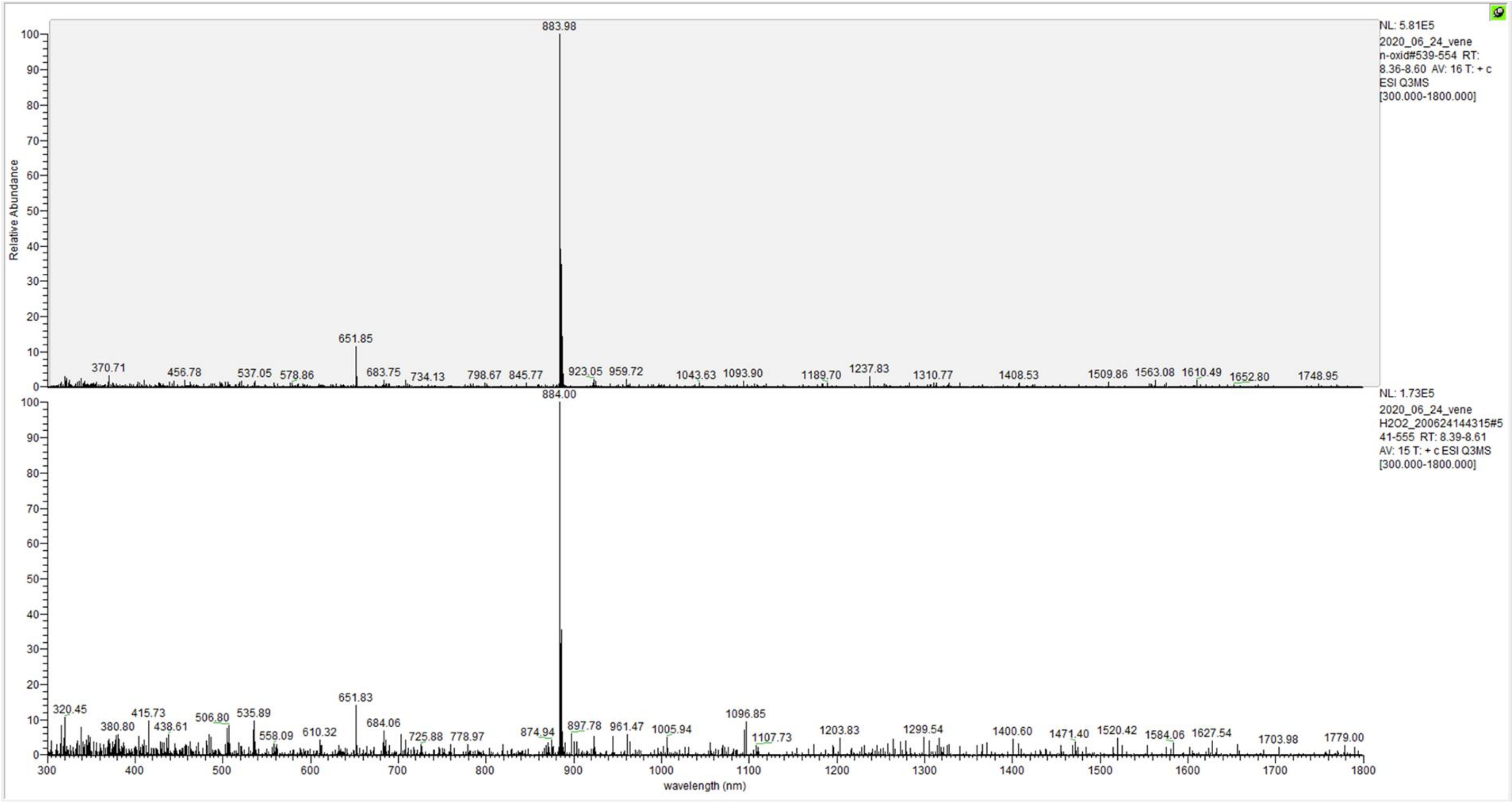

Figure S65 – MS spectra of a commercially obtained ***N*-oxide venetoclax** (top) and **degradation product *N*-oxide** in stress sample of venetoclax with added H_2O_2 (bottom). MS spectra was obtained with a triple quad mass spectrometer.
